# Structural basis of T-loop-independent recognition and activation of CDKs by the CDK-activating kinase

**DOI:** 10.1126/science.adw0053

**Published:** 2025-10-16

**Authors:** Victoria I. Cushing, Amy J.S. McGeoch, Sophie L. Williams, Theodoros I. Roumeliotis, Junjie Feng, Lucy M. Dan, Jyoti S. Choudhary, Norman E. Davey, Basil J. Greber

**Affiliations:** 1 Division of Structural Biology, The Institute of Cancer Research; 237 Fulham Road, London, SW3 6JB, UK; 2 Division of Cell and Molecular Biology, The Institute of Cancer Research; 237 Fulham Road, London, SW3 6JB, UK; 3 Functional Proteomics Group, The Institute of Cancer Research; 237 Fulham Road, London, SW3 6JB, UK; 4 CRUK Convergence Science Centre at The Institute of Cancer Research and Imperial College; London, UK

## Abstract

Cyclin-dependent kinases (CDKs) are prototypical regulators of the cell cycle. The CDK-activating kinase (CAK) acts as a master regulator of CDK activity by catalysing the activating phosphorylation of CDKs on a conserved threonine residue within the regulatory T-loop. However, structural data illuminating the mechanism by which the CAK recognises and activates CDKs have remained elusive. Here, we determine high-resolution structures of the CAK in complex with CDK2 and CDK2-cyclin A2 by cryogenic electron microscopy. Our structures reveal a T-loop-independent kinase-kinase interface with contributions from both kinase lobes. Computational analysis and structures of CAK in complex with CDK1-cyclin B1 and CDK11 indicate that these structures represent the general architecture of CAK-CDK complexes. These results advance our mechanistic understanding of cell cycle regulation and kinase signalling cascades.

The cyclin-dependent kinases (CDKs) are a family of serine/threonine kinases with important functions in the regulation of the cell cycle and transcription (1). In humans, CDKs 1, 2, 4, and 6 are the major mediators of cell cycle control. Aberrant CDK activity is associated with uncontrolled cell proliferation and tumorigenesis, and CDKs have emerged as attractive drug targets for cancer treatment (2, 3). Control of CDK activity in the cell is mediated by both inhibitory and activating phosphorylation and by binding to regulatory proteins, including CDK inhibitor proteins and positive regulatory subunits known as cyclins (4).

CDKs require cyclin binding for basal activation, but for maximal activity they must also be phosphorylated on a conserved threonine residue (Thr160 in human CDK2) (5, 6) within the activation segment or T-loop of the kinase. In metazoans, this activating phosphorylation is catalysed by the CDK-activating kinase (CAK), itself a CDK-cyclin complex consisting of CDK7, cyclin H, and MAT1 (7–10). The CAK integrates regulation of both CDK activity and transcription, as it also phosphorylates the C-terminal domain (CTD) of the largest subunit of RNA polymerase II (Pol II) as a component of the general transcription factor IIH (TFIIH) (11). Inhibition of CDK7 by small molecules leads to apoptosis or cell cycle arrest (12–15), and its genetic ablation causes embryonic lethality in mice (16), underscoring the importance of the CAK for cellular physiology.

Crystallographic studies of monomeric and cyclin-bound CDK2 have revealed the structural rearrangements that occur upon cyclin binding and T-loop phosphorylation during CDK activation (17–19). However, we currently lack mechanistic insight into how the CAK recognises and activates its CDK substrates. Biochemical evidence indicates that recognition of CDKs by the CAK is independent of the T-loop sequence and is instead driven by protein-protein interactions away from the phosphorylation site (20, 21). Nonetheless, the nature of the interactions and, thus, the basis of T-loop-independent recognition and activation of CDKs by the CAK have remained unclear.

Here, we use cryogenic electron microscopy (cryo-EM) for structure determination of the CAK in complex with CDK2 in both cyclin-bound and monomeric states. We present structures of CAK-CDK2-cyclin A2 bound to AMP-PNP and in the nucleotide-free (apo) state and of CAK-CDK2 in the presence of ADP-nitrate or ADP-AlF_x_ at resolutions of 2.4-3.2 Å. These structures reveal the structural basis of T-loop-independent recognition of CDK2 by the CAK, thereby adding key insights to our mechanistic understanding of CDK activation. Computational analysis and structure determination of CAK-CDK1-cyclin B1 and CAK-CDK11 complexes suggest that these structures represent the general architecture of CAK-CDK complexes.

## Results

### Structures of the CAK in complex with CDK2 and CDK2-cyclin A2 reveal a pseudo-symmetric kinase-kinase interface

The CAK can phosphorylate both monomeric and cyclin-bound CDK2 in vitro. However, the kinetic parameters of the phosphorylation reaction are different: Both k_cat, CDK2_ and K_M, CDK2_ are approximately one order of magnitude higher than k_cat, CDK2-cyclin A_ and K_M, CDK2-cyclin A_. Although this results in a similar catalytic efficiency (k_cat_/K_M_) overall, it indicates that CAK exhibits higher turnover on CDK2 and forms a more stable enzyme-substrate complex with CDK2-cyclin A (22). Biochemical and cell biological experiments using analogue-sensitive CDK7 established that CDK2 is likely to be the predominant physiological substrate (22). To understand the mechanism by which CAK binds and recognises its CDK substrates and to gain insight into why monomeric CDK2 is preferentially phosphorylated by the CAK, we aimed to structurally characterise complexes of the CAK bound to both monomeric and cyclin-bound CDK2.

We expressed and purified the human CAK from insect cells and expressed and purified monomeric CDK2 and CDK2-cyclin A2 from bacteria, thereby ensuring that the CDK2 T-loop was not phosphorylated. Using pull-down assays, we confirmed that we could obtain complexes of the CAK bound to either form of CDK2 (fig. S1). We observed that CDK2 was sub-stoichiometric in the CAK-CDK2 complex, in contrast to the fully stoichiometric CAK-CDK2-cyclin A2 complex (fig. S1, A to D). Along with analysis of cryo-EM 2D classes (fig. S1, E and F), this confirms the lower binding affinity of the CAK for monomeric compared to cyclin-bound CDK2 (22).

**Fig. 1:**
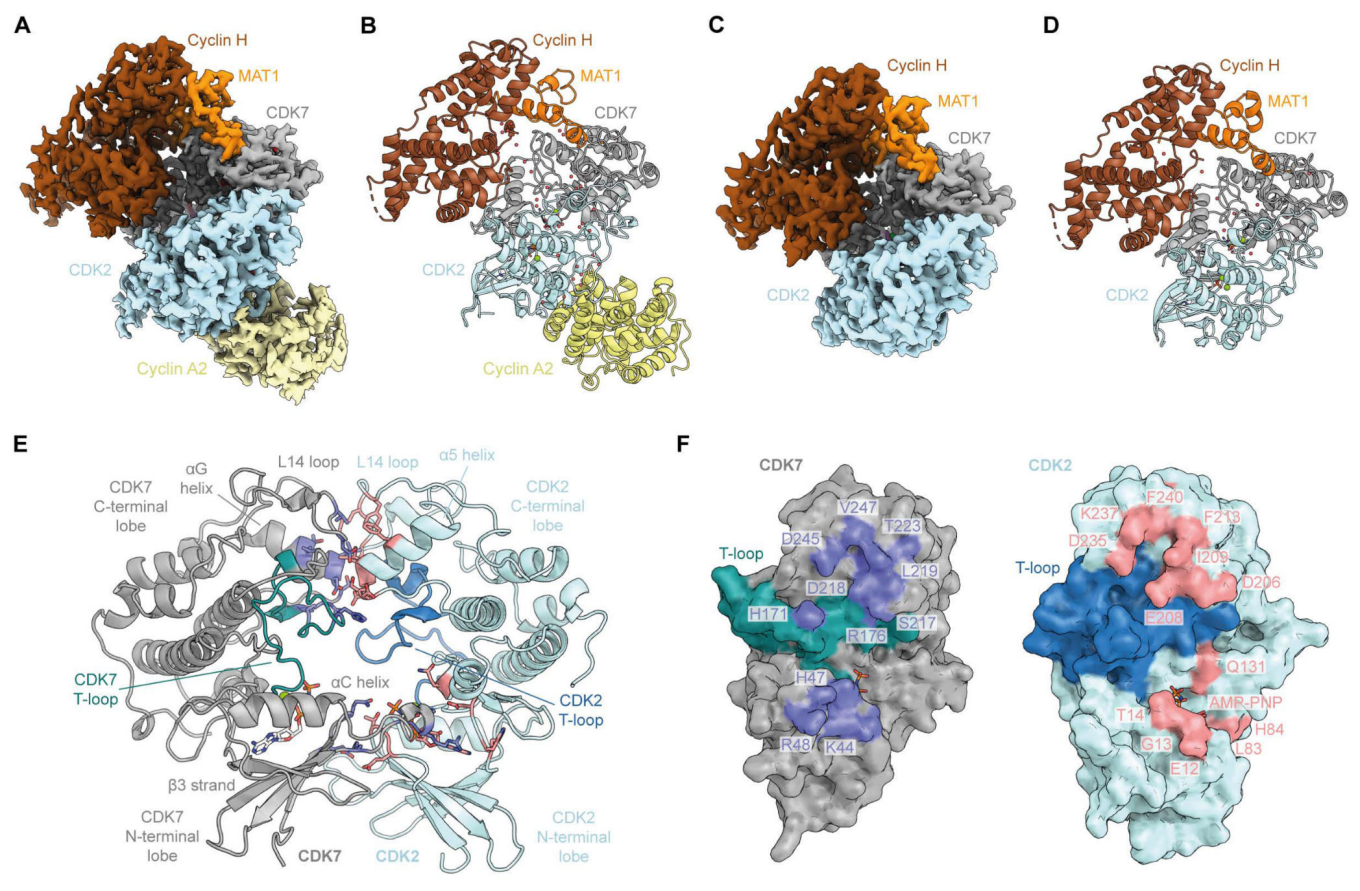
Cryo-EM structures of CAK-CDK2-cyclin A2 and CAK-CDK2 complexes. (**A**) Cryo-EM reconstruction of the CAK-CDK2-cyclin A2-AMP-PNP complex coloured by its constituent subunits. (**B**) Atomic model of the CAK-CDK2-cyclin A2-AMP-PNP complex. (**C**) Cryo-EM reconstruction of the CAK-CDK2 (ADP-AlF_x_) complex. (**D**) Atomic model of the CAK-CDK2 (ADP-AlF_x_) complex. Additional views of maps and models are provided in Fig. S2. (**E**) Close-up view of the CDK7-CDK2 interaction interface in the CAK-CDK2-cyclin A2-AMP-PNP complex (for clarity, only the two kinases are shown). Interacting residues of CDK7 and CDK2 are shown in purple and salmon, respectively. (**F**) Cross-section of the CDK7-CDK2 interaction interface in the CAK-CDK2-cyclin A2-AMP-PNP complex, shown as a surface representation. A full comparison of the cryo-EM structures of all CAK-CDK2-cyclin A2 and CAK-CDK2 complexes in this study and criteria for assigning interacting residues are provided in fig. S2.

We determined cryo-EM structures of the CAK in complex with CDK2 and with CDK2-cyclin A2 (Fig. 1, A to D, and fig. S2, A to H). We obtained cryo-EM reconstructions of CAK-CDK2 complexes assembled in the presence of ADP-AlF_x_ or ADP-nitrate at 2.4-Å and 3.1-Å global resolution and of the nucleotide-bound and apo-CAK-CDK2-cyclin A2 complexes at 2.5-Å and 2.6-Å global resolution, respectively (figs. S2 and S3, and Tables S1 and S2). The direct CDK7-CDK2 interactions observed in these structures are extremely similar across all complexes (Fig. 1E, fig. S2, I to K), explaining how the CAK is able to phosphorylate both monomeric and cyclin-bound CDK2 in vitro (22). The detailed analysis of CDK7-CDK2 interactions presented in the following sections is based on the CAK-CDK2-cyclin A2 structures.

Our data reveal that CDK7 and CDK2 interact via a pseudo-symmetrical head-to-head interface composed of two main interaction clusters in the N- and C-terminal lobes of the two kinases (Fig. 1, E and F). This contradicts an early computational modelling study that suggested a head-to-tail interaction between CDK7 and CDK2 (23) but is compatible with predictions from AlphaFold3 (see below) (24). Because CDK7 is not only the activating kinase for CDK2, but also a CDK2 substrate itself (21), the pseudo-symmetrical nature of the interface explains how both kinases are able to reciprocally activate one another. The CDK2 T-loop does not contribute meaningfully to the interface (Fig. 1E, F), thereby explaining why CDK recognition by the CAK is independent of the substrate’s T-loop sequence (20, 21).

### Molecular dissection of the kinase-kinase interface

**Fig. 2:**
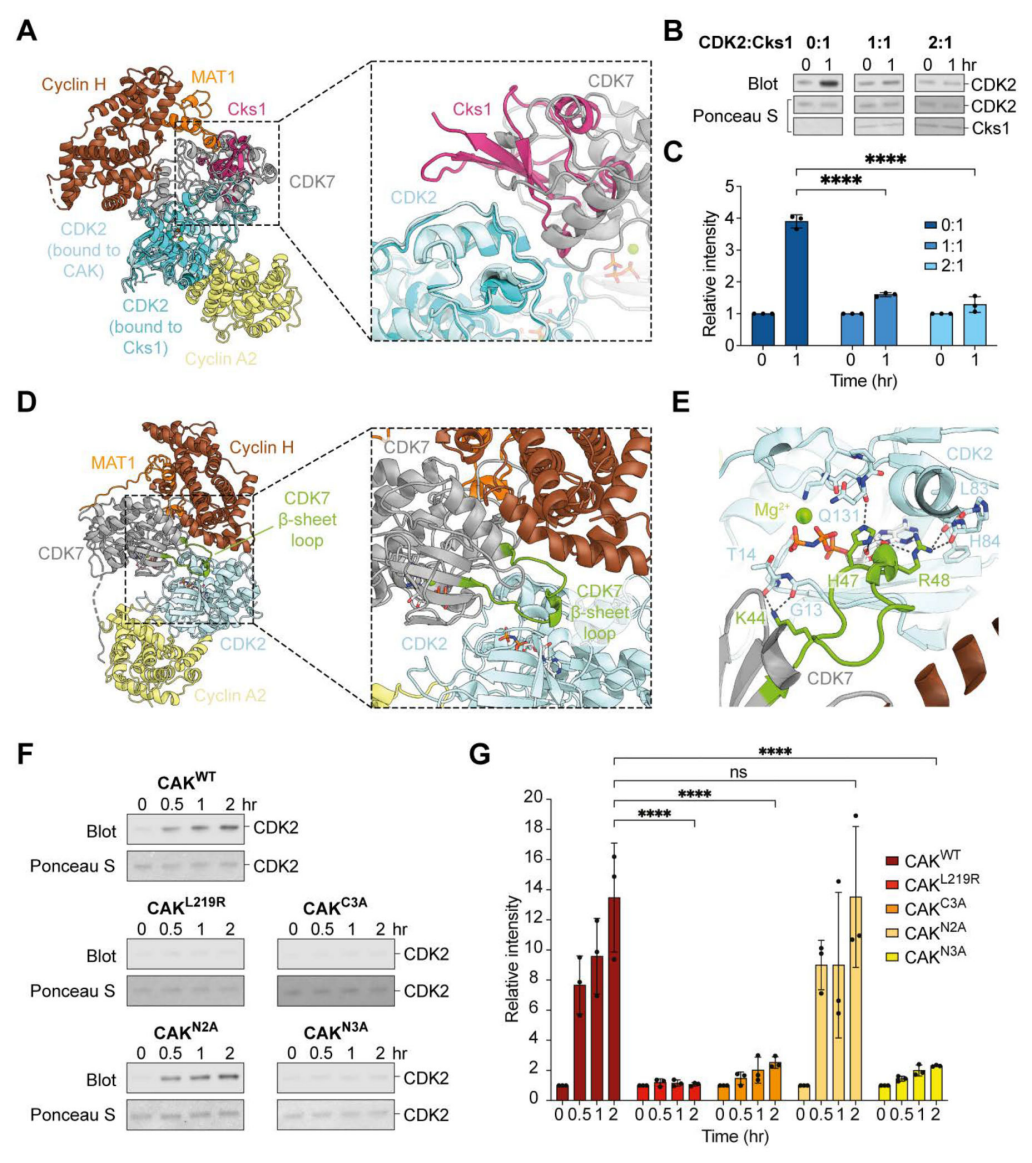
Analysis of the kinase-kinase interface. (**A**) Superposition of the CAK-CDK2-cyclin A2-AMP-PNP complex with CDK2-Cks1 (PDB 1BUH) (26). Inset: Close-up view of the overlap between Cks1 and CDK7 (MAT1 and cyclin H are omitted for clarity). (**B**) Western blots against phosphorylated CDK2 and Ponceau S-stained loading controls for assays assessing the activity of the CAK towards CDK2 in the presence/absence of Cks1. Two biologically independent sets of experiments were performed, each with N = 3 technical replicates. One representative technical replicate from one set of experiments is shown here; remaining replicates are shown in fig. S4, C and D. (**C**) Quantification of Western blot band intensities for one set of experiments (including the data shown in panel B), presented as the mean ± standard deviation of N = 3 technical replicates. *P*-values obtained by two-way ANOVA and Tukey’s multiple comparisons test: 0:1 1 hr vs. 1:1 1 hr *P* < 0.0001; 0:1 1 hr vs. 2:1 1 hr *P* < 0.0001 (**** = *P* ≤ 0.0001). The plot for the second set of experiments is shown in fig. S4E. (**D**) Side view of the CAK-CDK2-cyclin A2-AMP-PNP complex. Inset: Close-up of the CDK7 β-sheet loop inserting into the CDK2 N-terminal lobe. (**E**) Another view of the CDK7 β-sheet loop in the CAK-CDK2-cyclin A2-AMP-PNP complex. CDK7 loop residue K44 mediates backbone contacts with CDK2 residues, while H47 and R48 insert into the CDK2 active site. (**F**) Western blots against phosphorylated CDK2 and Ponceau S-stained loading controls for assays assessing the activity of CAK^WT^, CAK^L219R^, CAK^C3A^, CAK^N2A^, and CAK^N3A^ towards CDK2. Two biologically independent sets of experiments were performed, each with N = 3 technical replicates. One representative technical replicate from one set of experiments is shown here; remaining replicates are shown in fig. S5, C and D. (**G**) Quantification of Western blot band intensities for one set of experiments (including the data shown in panel F), presented as the mean ± standard deviation of N = 3 technical replicates. *P*-values obtained by two-way ANOVA and Tukey’s multiple comparisons test: CAK^WT^ 2 hr vs. CAK^L219R^ 2 hr *P* < 0.0001; CAK^WT^ 2 hr vs. CAK^C3A^ 2 hr *P* < 0.0001; CAK^WT^ 2 hr vs. CAK^N2A^ 2 hr *P* > 0.9999; CAK^WT^ 2 hr vs. CAK^N3A^ 2 hr *P* < 0.0001 (**** = *P* ≤ 0.0001, ns = *P* > 0.05). The plot for the second set of experiments is shown in fig. S5E.

The interaction cluster between the CDK7 and CDK2 C-terminal lobes primarily consists of residues within the CDK7 αG helix and L14 loop that interact with the CDK2 α5 helix and L14 loop (4, 25) (Fig. 1E). Polar and hydrophobic side chains from both kinases contribute a hydrogen bonding network and hydrophobic interactions at this interface (Fig. 1, E and F). This region of the CDK2 C-terminal lobe is the same interface employed by CDK2 when binding to Cks proteins (fig. S4, A and B) (26), which promote the recruitment of CDK1 and CDK2 to phosphorylated substrates (27). However, the order of Cks protein binding to CDKs and their activating phosphorylation by the CAK has remained ambiguous. Our structural data suggest that binding of the CAK and Cks proteins to CDK2 is mutually exclusive (Fig. 2A). Accordingly, we found that the presence of Cks1 resulted in a strong dose-dependent decrease in CAK activity towards CDK2 (Fig. 2, B and C, and fig. S4, C to E), thereby confirming that the presence of bound Cks1 is incompatible with CDK2 activation by CAK. In the context of CDK regulation, the activating phosphorylation of CDKs by the CAK must therefore occur before Cks protein binding (fig. S4F).

The N-terminal lobe interaction cluster is primarily formed by a loop between the β3 strand and αC helix of CDK7, which inserts into the CDK2 N-terminal lobe (Fig. 1E and Fig. 2, D and E). Given that this loop is not visualised in structures of the isolated human CAK (28–30) (fig. S5A) and CDK7 alone (25), our structures indicate that it becomes ordered upon substrate binding. In particular, the side chain of CDK7 loop residue Lys44 mediates backbone contacts with residues Gly13 and Thr14 of the CDK2 β1 strand. Additionally, the side chains of CDK7 loop residues His47 and Arg48 insert into the CDK2 active site in both apo- and nucleotide-bound complexes, where they contact the bound nucleotide (if present) and surrounding active site residues (Fig. 2E and fig. S5B).

We used mutagenesis to validate the two interaction clusters observed in the structures. The C-lobe hydrophobic and hydrogen bonding interaction networks were disrupted by generation of a CDK7 L219R mutant (CAK^L219R^) and a CDK7 R176A, S217A, T223A triple mutant (CAK^C3A^). The N-lobe interaction was targeted by generation of a CDK7 H47A, R48A double mutant (CAK^N2A^) and a CDK7 K44A, H47A, R48A triple mutant (CAK^N3A^). In-vitro kinase assays using CDK2 and the wild-type (WT) CAK complex (CAK^WT^) produced a substantial phospho-CDK2 signal, confirming that the WT enzyme is capable of rapidly phosphorylating CDK2 (Fig. 2, F and G, and fig. S5, C to E). Phosphorylation of CDK2 Thr160 was confirmed by mass spectrometry, thereby validating the antibody used (Data S1). By contrast, both C-lobe mutants showed no or minimal increase in phospho-CDK2 signal across two hours of incubation (Fig. 2, F and G, and fig. S5, C to E); loss of the C-lobe interface is therefore sufficient to abolish the ability of the CAK to phosphorylate CDK2. Although the activity of the N-lobe triple mutant (CAK^N3A^) is also reduced, the N-lobe double mutant (CAK^N2A^) behaves comparably to CAK^WT^, indicating that the insertion of the CDK7 N-lobe residues His47 and Arg48 into the CDK2 active site is not critical for CDK2 activation (Fig. 2, F and G, and fig. S5, C to E). These results suggest that both the N- and C-lobe interaction clusters between CDK7 and CDK2 are critical for CDK2 activation by CAK, and loss of either interaction cluster is sufficient to abolish the ability of the CAK to phosphorylate CDK2. Analysis of small cryo-EM datasets confirmed that all mutants retain their expected fold, ability to assemble into the CAK complex, and nucleotide-binding capability (fig. S6).

The reciprocal experiment was performed by mutagenesis of the C-lobe interface of CDK2. We generated a CDK2 I209R mutant (CDK2^I209R^), equivalent to the CDK7 L219R mutant. In our in-vitro kinase assay system, CDK2^I209R^ was unable to be phosphorylated by the CAK (fig. S7), thereby further validating the identified C-lobe CDK7-CDK2 interaction cluster. Nano-differential scanning fluorimetry (nano-DSF) thermal stability analysis indicates that the CDK2^I209R^ mutant is still folded (fig. S8).

### Evidence for a general architecture of CAK-CDK substrate recognition and activation complexes

**Fig. 3:**
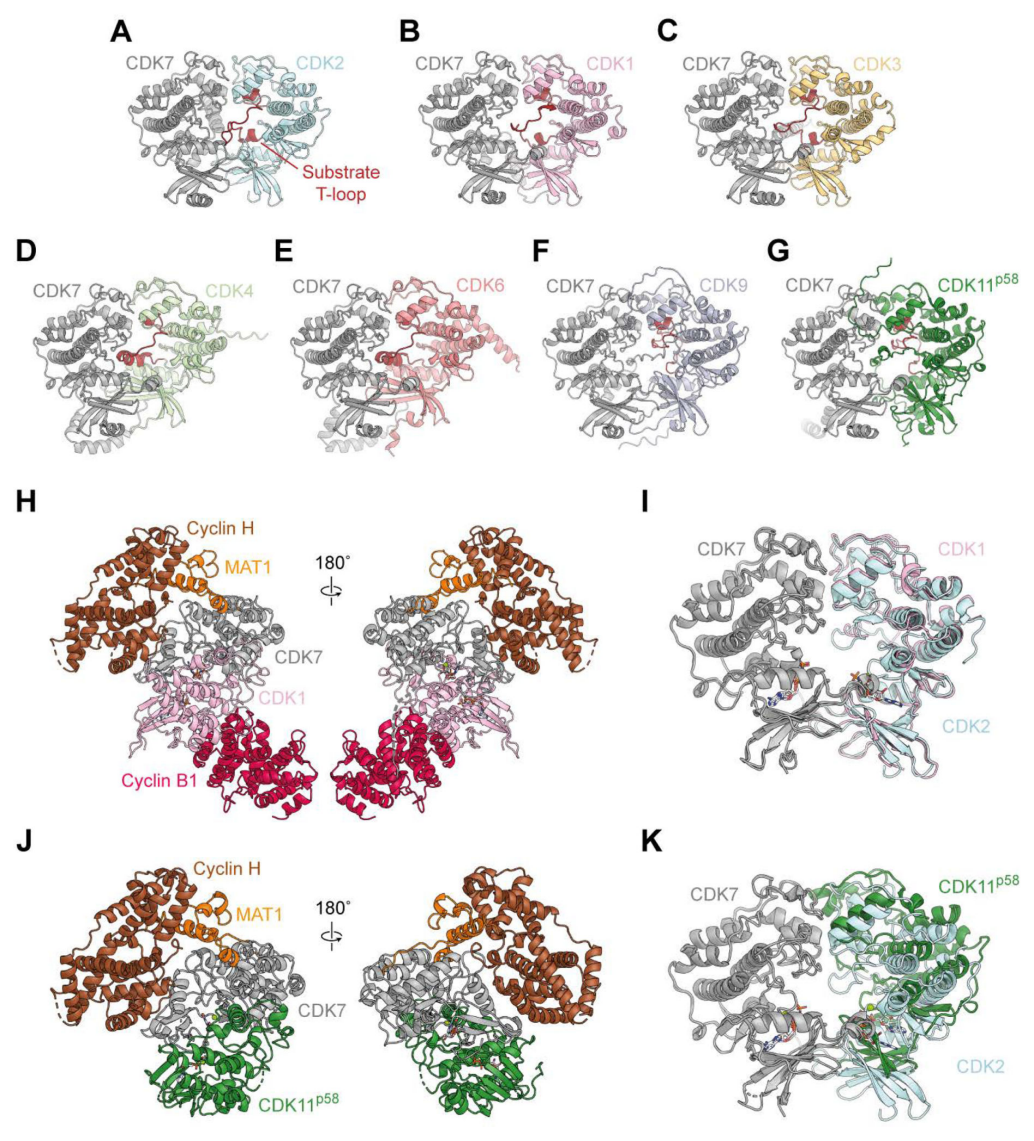
A general mechanism for CAK-CDK recognition. (**A-G**) AlphaFold3-predicted structures of CAKΔN (the CAK without the N-terminal 219 residues of MAT1) in complex with substrate CDKs. For clarity, only the two kinases are shown in each panel. The T-loop of the substrate is coloured red in each panel. Predicted local distance difference test (pLDDT) and predicted aligned error (PAE) plots (46) are provided in Fig. S10. (**H**) Cryo-EM structure of the CAK-CDK1-cyclin B1 complex. (**I**) Close-up of the kinase-kinase interface of the CAK-CDK1-cyclin B1 complex, superimposed with the kinase-kinase interface of the AMP-PNP-bound CAK-CDK2-cyclin A2 complex. (**J**) Cryo-EM structure of the CAK-CDK11 complex. (**K**) Close-up of the kinase-kinase interface of the CAK-CDK11 complex, superimposed with the kinase-kinase interface of the AMP-PNP-bound CAK-CDK2-cyclin A2 complex.

Multiple sequence alignment reveals that several of the identified interface residues in CDK2 are conserved or semi-conserved across other CDKs that are substrates of the CAK (fig. S9). For instance, residues with similar biochemical properties are found in the positions of Leu83, Ser207, Glu208, Ile209, and Phe240 in all or most other CDK substrates. This conservation suggests that our structures may represent a more general architecture of CAK-CDK complexes, applicable to the recognition and activation of other CDK substrates by the CAK. Therefore, we performed macromolecular structure prediction using AlphaFold3 (24) to predict the interaction interfaces formed by the CAK in complex with a range of CDKs (Fig. 3, A to G). AlphaFold3 correctly predicts the pseudo-symmetrical, head-to-head CDK7-CDK2 interface observed in our structures (Figs. 1E and 3A) and additionally predicts a very similar interface for complexes of the CAK with other CDKs that are known CAK substrates (Fig. 3, B to G). The predicted aligned errors (PAEs) of these interactions are low (fig. S10, A to G), suggesting a high level of confidence in the binding mode of these CDKs. By contrast, AlphaFold3 does not confidently predict such complexes between the CAK and known non-substrates CDK7 (21) or CDK8 (31), as indicated by higher PAEs for these predictions (fig. S10, H and I). This is also the case for the predicted complex between the CAK and CDK5 (fig. S10J); whereas CDK5 is a proposed substrate of the CAK in the brain (32), it cannot be phosphorylated in vitro by the CAK isolated from other tissues (32, 33), indicating that it may not be a direct CAK substrate. Hence, our structures likely depict a general kinase-kinase interaction architecture for the recognition and activation of CDKs by the human CAK.

To experimentally test this hypothesis, we determined additional cryo-EM structures of the CAK in complex with two other known CDK substrates: CDK1-cyclin B1 and CDK11. CDK11 is a kinase that functions in the regulation of transcription, mitotic progression, and splicing (34). Among CDKs, it is relatively distantly related to CDK2 (1) but is nonetheless a known CAK substrate (20), making it a good subject to test the validity of our hypothesis. CDK1 is a well established substrate of the CAK in cell cycle control, but is known to follow a different activation pathway compared to CDK2 (9, 22, 35). CDK2 likely follows a phosphorylation-first activation pathway *in vivo*, but CDK1 instead follows a concerted pathway, whereby phosphorylation and stable cyclin binding are interdependent (35).

We obtained cryo-EM reconstructions of CAK-CDK1-cyclin B1 bound to AMP-PNP and of CAK-CDK11^p58^ bound to ADP at global resolutions of 3.4 Å and 3.5 Å, respectively (Fig. 3, H to K, fig. S11, and Table S3). The kinase-kinase interfaces in both structures are essentially identical to that between the CAK and CDK2 (Fig. 3, I and K). Despite the different activation pathways of CDK1 and CDK2, the CAK-CDK1-cyclin B1 and CAK-CDK2-cyclin A2 structures are almost identical (Figs. 1B and 3H and fig. S12A), except for the slightly different conformation of the CDK1 T-loop, which is phosphorylated (fig. S12, B and C). The T-loop of CDK11 is also observed in an extended conformation, pointing away from the CDK7 active site. However, the CDK7-CDK11^p58^ interface is more conformationally flexible than the CDK7-CDK2 interface due to more dynamic N-lobe interactions (Fig. 3K), which was reflected in a lower resolution of the reconstruction. Overall, however, these data confirm that the CAK employs a common architecture of the kinase-kinase interface for the recognition and activation of its substrate CDKs.

### Interaction of a CDK7 C-terminal RxL motif with cyclins

The C-terminal tail of CDK7 possesses a peptide with the sequence PKKLIF (residues 341-346) that conforms to the consensus sequence known as the cyclin-binding (Cy), or RxL, motif. Generally, RxL motifs act as a recruitment peptides for CDK substrates by binding to a hydrophobic patch containing the conserved MRAIL sequence on the surface of cyclins (37).

**Fig. 4:**
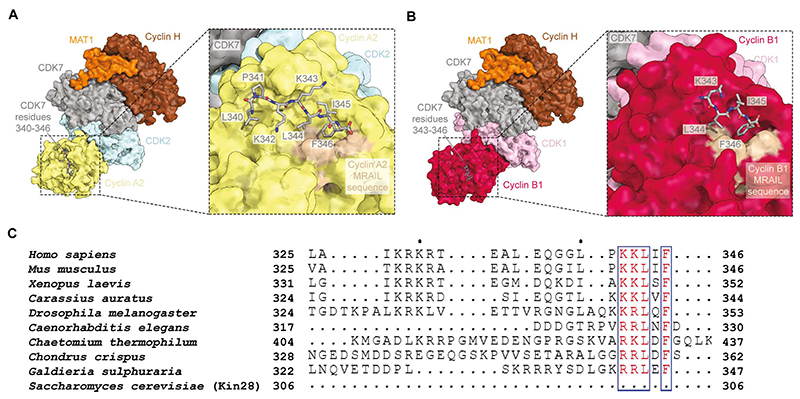
Interaction of the CDK7 C-terminal RxL motif with cyclins. (**A**) Surface representation of the apo-CAK-CDK2-cyclin A2 complex with CDK7 residues Lys340-Phe346 shown as sticks. Inset: Close-up view showing CDK7 residues Lys340-Phe346 modelled in the cyclin A2 hydrophobic patch. (**B**) Surface representation of the CAK-CDK1-cyclin B1 complex with CDK7 residues Lys343-Phe346 shown as sticks. Inset: Close-up view showing CDK7 residues Lys343-Phe346 modelled in the cyclin B1 hydrophobic patch. (**C**) Multiple sequence alignment of C-terminal sequences of CDK7 homologues from different organisms showing conservation of the RxL motif. The alignment includes the C-terminal sequence of *S. cerevisiae* Kin28, a homologue of CDK7 in budding yeast that possesses neither CDK-activating kinase activity nor the C-terminal extension containing the RxL motif. The complete alignment is shown in fig. S14.

After modelling the core complex in our structure of apo-CAK-CDK2-cyclin A2, we observed additional unmodelled density in the hydrophobic groove of cyclin A2 (fig. S13A). This density supports modelling of the CDK7 C-terminal PKKLIF peptide, as well as the preceding Leu340 residue (Fig. 4A). Local, masked refinement of the CDK2-cyclin A portion of the CAK-CDK2-cyclin A2- AMP-PNP reconstruction yielded a 2.6-Å resolution map that is consistent with this interpretation (fig. S13, B to H). Additional density was also observed in the hydrophobic patch of cyclin B1 in the CAK-CDK1-cyclin B1 reconstruction (fig. S13I). This density is of poorer quality compared to the CAK-CDK2-cyclin A2 structures, possibly because of residual flexibility or because the cyclin B hydrophobic patch is predicted to bind RxL motifs more weakly than cyclin A (38), but it nonetheless permits modelling of residues Lys343-Phe346 of CDK7 (Fig. 4B).

Kin28, a CDK7 homologue in *S. cerevisiae*, possesses kinase activity towards the Pol II CTD but lacks both CAK activity (11) and the C-terminal extension that contains this RxL motif (Fig. 4C and fig. S14). Furthermore, AlphaFold3 confidently predicts interactions between the CDK7 RxL motif and several cyclins, including the CDK-associated cyclins B1 (in agreement with our CAK-CDK1-cyclin B1 structure), D1, and E1 (fig. S15, A to D). This is consistent with a possible function of the RxL motif of CDK7 in its role as the CAK. Considering that CDK2, without bound cyclin and thus unable to form this RxL interaction, is the preferred CAK substrate (22), binding of the CDK7 C-terminus to cyclin A is unlikely to serve to improve CDK2 recruitment to the CAK; however, interaction of the CDK7 C-terminus with cyclins other than cyclin A2 might support recruitment of CDK-cyclin complexes that are preferentially phosphorylated in their dimeric form, such as CDK1-cyclin B. Alternatively, it is possible that this sequence serves as a recruitment peptide for CDK7 as a substrate of CDK2-cyclin and CDK1-cyclin complexes. This is because the CDK7 T-loop itself possesses two phosphorylation sites (Ser164 and Thr170 in human CDK7) that are targets of CDK1 and CDK2 (21, 36). The functions associated with the CDK7 RxL motif are likely conserved, given the presence of this motif in CDK7 homologues across several species, including some fungi and plants (Fig. 4C, fig. S14, S16).

### Enhanced CDK2 T-loop flexibility in the absence of bound cyclin

**Fig. 5:**
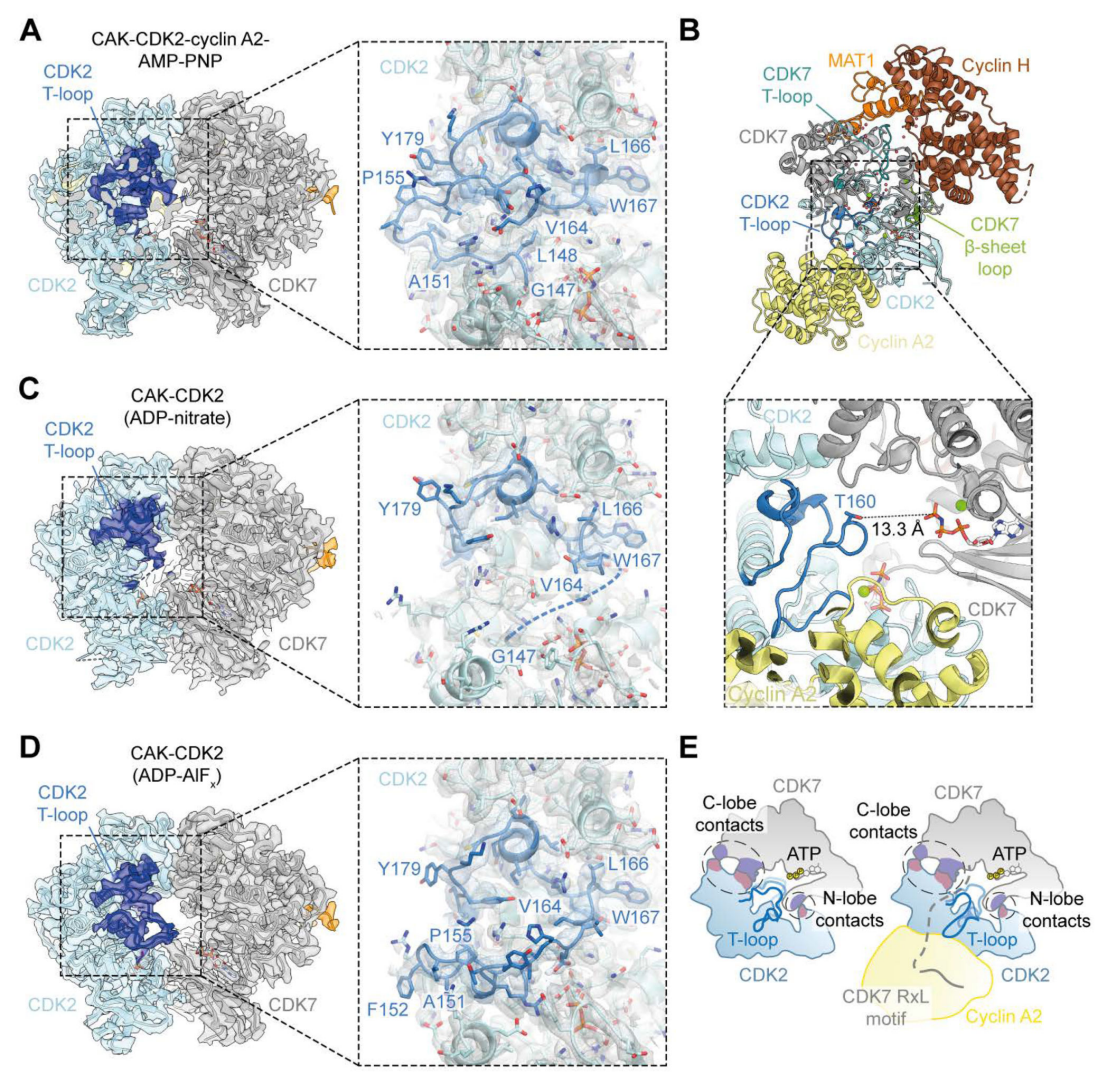
Increased CDK2 T-loop dynamics of CAK-CDK2 complexes. (**A**) Map-model fit of the kinase-kinase interface of the CAK-CDK2-cyclin A2-AMP-PNP complex. Inset: Close-up of the CDK2 T-loop showing that it adopts a well-ordered conformation similar to that seen in isolated CDK2-cyclin complexes. (**B**) Atomic model of the CAK-CDK2-cyclin A2-AMP-PNP complex. Inset: Close-up of the CDK2 T-loop. Thr160, the phosphorylation target residue, sits 13.3 Å away from the γ-phosphate of the CDK7 active site nucleotide. (**C**) Map-model fit of the kinase-kinase interface of the CAK-CDK2 (ADP-nitrate) complex. Inset: Close-up of the CDK2 T-loop showing that it is highly flexible, as indicated by a lack of density for the segment between G147 and V164 (dotted line). The views are the same as in panel A. (**D**) Map-model fit of the kinase-kinase interface of the CAK-CDK2 (ADP-AlF_x_) complex. Inset: Close-up of the CDK2 T-loop showing that it adopts an intermediate conformation. The views are the same as in panels A and C. (**E**) Schematic illustrating the proposed model of CDK recognition and activation by the CAK. MAT1 and cyclin H are not depicted for clarity.

In our structures of the CAK-CDK2-cyclin A2 complex, the CDK2 T-loop adopts a rigid conformation, as indicated by well-defined density (Fig. 5A), almost identical to that seen in structures of cyclin-bound, unphosphorylated CDK2 (18). In this conformation, the phosphorylation target residue, Thr160, sits approximately 13 Å away from the γ-phosphate of the CDK7 active site nucleotide (Fig. 5, A and B), which is incompatible with phosphoryl transfer by CDK7. Hence, this structure likely represents a post-recognition, pre-catalyic complex. The formation of contacts between the CDK2 T-loop and cyclin A (buried surface area 480 Å^2^) suggests that the T-loop is constrained from readily entering the CDK7 active site by the presence of the cyclin. This may contribute to the lower rate of phosphorylation of cyclin-bound CDK2 compared to CDK2. To investigate this hypothesis, we turned to our cryo-EM structures of the CAK bound to monomeric CDK2. These structures have been obtained in the presence of either ADP-nitrate or ADP-AlF_x_, which have been used previously to mimic phosphorylation transition states in kinases, including CDKs (39–41). Although the nitrate or AlF_x_ groups were not observed in our cryo-EM maps, these reconstructions still provided additional insights into the T-loop conformations of CAK-bound CDKs.

The CAK-CDK2 complex adopts an identical kinase-kinase interface as observed for the cyclin-bound complexes (Fig. 1E, fig. S2, I to K), but the density for the CDK2 T-loop in the cyclin-free complex determined in the presence of ADP-nitrate is virtually non-existent (Fig. 5C). This suggests increased T-loop flexibility in the cyclin-free complex. This is in agreement with the higher conformational variability of the T-loop in free CDK2 and its higher propensity to assume conformations that expose Thr160 to the solvent compared to CDK2-cyclin A, as determined by nuclear magnetic resonance experiments (42). Interestingly, the CDK2 T-loop in the CAK-CDK2 ADP-AlF_x_ complex is visualised, but in a different conformation compared to the CAK-CDK2-cyclin A2 complexes (Fig. 5, A and D, and fig. S17A). This conformation is intermediate between those of free CDK2 (43) and CDK2-cyclin A (18) (fig. S17, B and C) and is likely visualised because the otherwise flexible T-loop is trapped by salt bridges between CDK2 residue Glu162 and the Mg^2+^ ions in the CDK2 active site (fig. S17D).

The increased flexibility of the CDK2 T-loop in the absence of cyclin A2 probably increases the likelihood of Thr160 insertion into the CDK7 active site. This may help explain why the CAK has a higher enzyme turnover, indicating a higher rate of phosphorylation, on monomeric compared to cyclin-bound CDK2 (22). We identify two possible reasons for the decreased flexibility of the T-loop in the presence of cyclin A2: First, the T-loop forms additional interactions with the cyclin, which are likely to stabilise an extended T-loop conformation, pointing away from the CDK7 active site, and thereby reducing the propensity of the T-loop to sample conformations compatible with active site insertion. Second, a superposition of the structure of free, inactive CDK2 (PDB 1B38, (43)) onto our cryo-EM structures shows that the conformation of the unphosphorylated T-loop in free CDK2 approaches the CDK7 active site (fig. S17C), but that this conformation is sterically incompatible with the presence of cyclin A2 (fig. S17, E to G). We note that despite the steric constraints imposed by the presence of cyclin A2, CDK2-cyclin A2 is still phosphorylated in our in vitro kinase assay system, albeit more slowly than monomeric CDK2 (fig. S18), in agreement with published data (22).

## Discussion

Taken together, our observations allow us to propose a mechanistic model for CDK recognition and activation by the CAK (Fig. 5E). According to this model, stable N- and C-lobe contacts establish the CAK-substrate complex and determine substrate specificity, while flexibility of the substrate’s T-loop determines the kinetics of phosphorylation by the CAK once the enzyme-substrate complex has been formed. Our data combined with structure predictions suggest that the first step – substrate recognition and binding – occurs via a set of interactions that are broadly similar across different CAK substrates. However, the properties of different substrate CDKs and their associated cyclins may enable substrate-specific fine-tuning of the activity of CAK, such as a preference for free or substrate-bound CDK substrates. In the case of CDK2, a bound cyclin may (i) stabilise the enzyme-substrate complex due to the interaction formed by the CDK7 RxL motif, delaying substrate release and thereby reducing turnover and (ii) restrict the flexibility of the T-loop, thereby slowing the access of the T-loop to the active site.

Kinase signalling cascades in which upstream kinases bind and activate downstream kinases are key to many signal transduction pathways in human cells. Our high-resolution structures of the kinase-kinase interface of the CAK-CDK2 complex may therefore inform on the activation mechanism of other kinases within signalling pathways. Indeed, the CAK-CDK2 interface resembles that of the activation complex observed between the mitogen-activated protein kinase (MAPK) p38α and its activating kinase MKK6 at lower resolution (44). Our cryo-EM maps directly visualise the molecular details of the interaction interfaces between the two kinases, while the p38α-MKK6 complex exhibits more flexibility, possibly due to a weaker N-lobe interaction (44). Molecular dynamics simulations of the p38α-MKK6 complex, revealing that the p38α activation loop is conformationally variable and thereby able to sample conformations approaching the MKK6 catalytic site (44), are in agreement with our model of the role of CDK T-loop flexibility in access of the substrate to the kinase catalytic site. The mechanistic insights provided by our structures are therefore not restricted to CAK-CDK complexes but provide a structural framework for the analysis of more general kinase-kinase activation mechanism applicable to the activation of CMGC (CDK, MAPK, glycogen synthase kinase, and CDK-like kinase) group kinases more widely.

The kinase CDK7 within the CAK is the target of multiple active drug discovery programmes (15, 45). Discovery of highly specific CDK inhibitors is challenging due to the presence of 20 members of this protein family within human cells, some of which share high homology in the active site region (1, 3). Our structural results may facilitate the discovery of small molecule or peptide therapeutics that specifically target CDK activation by CDK7 while mitigating off-target effects.

## Supplementary Material

Data S1

Supplementary Materials

## Data Availability

Cryo-EM maps generated in this study have been deposited to the Electron Microscopy Data Bank (EMDB) with accession codes EMD-53027, EMD-53028, EMD-52759, EMD-52760, EMD-52761, EMD-52758 and EMD-54971. Atomic coordinates generated in this study have been deposited to the Protein Data Bank (PDB) with accession codes pdb_00009QCV, pdb_00009QCX, pdb_00009I9K, pdb_00009I9J, pdb_00009I9I, and pdb_00009SKQ. Mass spectrometry data have been deposited to the ProteomeXchange Consortium via the PRIDE partner repository with accession code accession code PXD068021. All other data are provided in the paper or Supplementary Materials. All materials generated in this study are available form the authors without a Materials Transfer Agreement or can be produced according to the detailed methods in the Supplementary Materials.
