# Supplementary material for: Structural basis of T-loop-independent recognition and activation of CDKs by the CDK-activating kinase
Source: Science. Author manuscript; Available in PMC 2025 Oct 24. (PMC7618291; doi:10.1126/science.adw0053)
Supplement: Supplementary Materials [file EMS209656-supplement-Supplementary_Materials.pdf]

Science AAAS

Supplementary Materials for

Structural basis of T-loop-independent recognition and activation of CDKs by the CDK-activating kinase

Victoria I. Cushing, Amy J. S. McGeoch, Sophie L. Williams, Theodoros I. Roumeliotis, Junjie Feng, Lucy M. Dan, Jyoti S. Choudhary, Norman E. Davey, Basil J. Greber*

* Corresponding author: Basil J. Greber, basil.greber@icr.ac.uk

This PDF file contains:

Materials and Methods
Figures S1 to S24
Tables S1 to S4
References (*47-68*)

Other Supplementary Materials for this manuscript include the following:

Data S1
MDAR Reproducibility Checklist

Materials and Methods

Protein expression and purification

CDK2 and CDK2-cyclin A2: The CDK2 construct consisted of residues 1-282 of human CDK2 with an N-terminal His_{10} tag. This was purchased from Addgene (Addgene plasmid #79726; http://n2t.net/addgene:79726; RRID:Addgene_79726) , deposited by J. Chodera, N. Levinson, and M. Seeliger (*47*). The cyclin A2 construct was obtained by cloning the human cyclin A2 sequence (provided by J. Pines) into a bacterial expression vector containing an N-terminal His_6 tag. A $CDK2^{I209R}$ mutant construct was generated by performing PCR to linearise the wild-type (WT) plasmid and remove the region to be mutated, which was replaced by insertion of a mutant g-block sequence (Integrated DNA Technologies). A $CDK2^{D145N}$ mutant construct was generated by performing PCR of the WT plasmid using mutagenic primers. DNA sequences of primers and g-blocks used in this study are given in Table S4. For each construct, 10 ml cultures of LB containing 100 µg/ml ampicillin and 34 µg/ml chloramphenicol were each inoculated with a single colony of transformed *E. coli* Rosetta (DE3) pLysS cells and incubated at 37 ˚C overnight. 1 L cultures of LB containing 100 µg/ml ampicillin and 34 µg/ml chloramphenicol were inoculated with the entirety of a single overnight culture and incubated at 37 ˚C for ~3 hours. At OD ~0.6, cultures were induced with 1 mM IPTG before incubation at 24 ˚C overnight. Cultures were harvested by spinning at 10,000 x g for 10 minutes at 4 ˚C using a JLA 8.1000 rotor (Beckman Coulter), before pellets were resuspended in a small amount of supernatant for transfer to smaller tubes. These were spun at 4,000 rpm (approx. 3,000 x g) for 15 minutes at 4 ˚C using an SX4250 rotor (Beckman Coulter). Pellets were snap frozen in liquid nitrogen for storage at -80 ˚C.

For purification of CDK2-cyclin A2 complexes, CDK2 pellet was combined with an excess of cyclin A2 pellet and resuspended in purification buffer A (50 mM HEPES pH 7.5, 180 mM NaCl, 5 % (v/v) glycerol, 2 mM $MgCl_2$, 2 mM DTT) supplemented with protease inhibitors, DNaseI, and 10 mM imidazole. The cell suspension was lysed by sonication and the lysate clarified by spinning at 18,000 rpm (approx. 19,000 x g) for at least 30 minutes at 4 ˚C using an F0650 rotor (Beckman Coulter). The supernatant was filtered through a Minisart NML 0.45 µm polyethersulfone syringe filter (Sartorius) to remove remaining cell debris before loading onto an equilibrated 5 ml HisTrap column (Cytiva). The column was washed with purification buffer A supplemented with 20 mM imidazole. Elution was performed using a linear gradient of 20-300 mM or 20-500 mM imidazole across 60 ml. Selected fractions were combined and concentrated before incubating with TEV protease (at a 1:10 or 1:20 ratio of protease:protein by mass) at 4 ˚C overnight to cleave the tags. To remove/reduce the imidazole content, either buffer exchange was performed before cleaving the tags using a HiPrep 26/10 desalting column (Cytiva) equilibrated with gel filtration buffer A (20 mM HEPES pH 7.5, 150 mM NaCl, 5 % (v/v) glycerol, 2 mM $MgCl_2$, 2 mM DTT), or the sample was diluted in gel filtration buffer A after cleaving the tags. The sample was incubated with equilibrated Ni-NTA Superflow beads (Qiagen) at room temperature (RT) for ~30 minutes to remove the His_6-tagged protease. The flow-through was concentrated and spun at 14,000 rpm (approx. 20,000 x *g*) for 15 minutes at 4 ˚C in an FA-45-12-17 rotor (Eppendorf) to pellet aggregates before gel filtration, which was performed using a Superdex 200 Increase 10/300 column (Cytiva). Selected fractions were combined, aliquotted, snap frozen in liquid nitrogen, and stored at -80 ˚C. An additional preparation of CDK2-cyclin A2 was generated using a construct of Strep-cyclin A2 and His_{10}-CDK2, for use as a third biological replicate in pull-down assays. This was purified via StrepTactin and Ni-NTA affinity chromatography followed by size exclusion chromatography. A frozen sample was thawed and subjected to tag cleavage by TEV protease before another size exclusion chromatography step to isolate untagged complex.

For purification of monomeric CDK2, pellets were resuspended in purification buffer B (40 mM HEPES pH 7.9, 250 mM KCl, 10 % (v/v) glycerol, 2 mM $MgCl_2$, 5 mM β-mercaptoethanol) supplemented with protease inhibitors and DNase I, and 10 mM imidazole, then lysed by sonication. The lysate was clarified by spinning at 18,000 rpm (approx. 19,000 x g) for 30 minutes at 4 ˚C using an F0650 rotor, then incubated with equilibrated Ni-NTA Superflow beads for 30 minutes at 4 ˚C. The beads were washed with purification buffer B supplemented with 25 mM imidazole before elution with purification buffer B supplemented with 300 mM imidazole. To remove imidazole, the sample was subjected to buffer exchange on a Sephadex G25 desalting column (Cytiva) or HiPrep 26/10 desalting column equilibrated with gel filtration buffer B (25 mM HEPES pH 7.9, 200 mM KCl, 5 % (v/v) glycerol, 2 mM $MgCl_2$, 5 mM β-mercaptoethanol). Output protein was then incubated with TEV protease (at a protease:protein ratio of 1:10 by mass) for 2 hr at RT, or overnight at 4 ˚C, to remove the tag. After cleavage, the sample was incubated with equilibrated Ni-NTA Superflow beads for 30 minutes at RT to remove the His_6-tagged protease. The flow-through was then further purified by gel filtration using a Superdex 75 10/300 column (Cytiva) or Superdex 200 Increase 10/300 column; eluted fractions were combined, aliquotted, and snap frozen in liquid nitrogen for storage at -80 ˚C.

CAK: Expression constructs for CAK complexes were plasmids of the 438-series (*48*) for expression in insect cells. Mutant CDK7 expression plasmids were generated via the same method as for the $CDK2^{I209R}$ construct (see Table S4 for sequences of primers and g-blocks). WT and mutant CAK complexes for use in assays and for CAK-CDK11 structure determination were obtained by infecting Sf9 (*Spodoptera frugiperda*) cells (Thermo Fisher Scientific, cell line Sf9, cat. no. 11496015, RRID:CVCL_0549) with bacmid DNA to generate separate baculoviruses encoding Strep-CDK7 or His_6-MAT1 along with untagged cyclin H. These were used for co-expression in High Five (*Trichoplusia ni*) cells (Thermo Fisher Scientific, cell line BTI-TN-5B1-4, cat. no. B85502, RRID:CVCL_C190) by supplementing two 500 ml cell cultures with 10 ml of each virus. WT CAK for structural studies of CAK-CDK2(-cyclin A2) complexes was expressed in High Five cells using a single baculovirus encoding all three subunits. Cultures were incubated at 27 ˚C for 72 hours before harvesting by spinning at 1,500 x g for 10 minutes at 4 ˚C using a JS-4.750 rotor (Beckman Coulter), then pellets were snap frozen in liquid nitrogen for storage at -80 ˚C.

Pellets were resuspended in purification buffer B supplemented with protease inhibitors, DNaseI, and 10 mM imidazole and lysed by sonication. The lysate was clarified as described, then incubated with equilibrated Ni-NTA Superflow beads for 45 minutes at 4 ˚C. The beads were washed with purification buffer B supplemented with 25 mM imidazole before elution with purification buffer B supplemented with 300 mM imidazole. The eluted fractions were incubated with equilibrated StrepTactin Superflow Plus beads (Qiagen) or StrepTactin Superflow beads (IBA) for 45 minutes at 4 ˚C. The beads were washed with purification buffer B before elution with purification buffer B supplemented with 10 mM desthiobiotin. The eluted fractions were further purified by gel filtration using a Superdex 200 Increase 10/300 column, with gel filtration buffer C (20 mM HEPES pH 7.9, 200 mM KCl, 5 % (v/v) glycerol, 2 mM $MgCl_2$, 5 mM β-mercaptoethanol). Selected fractions were combined, concentrated to approx. 2.0 mg/ml, aliquotted, snap frozen in liquid nitrogen, and stored at -80 ˚C.

CDK1-cyclin B1: CDK1 and cyclin B1 were expressed and purified separately before being combined to form the CDK1-cyclin B1 complex. Expression constructs of pLIB-GST-3C-CDK1 (Addgene plasmid #177011; http://n2t.net/addgene:177011; RRID:Addgene_177011), pLIB-HIS-TEV-scCAK1 (Addgene plasmid #177014; http://n2t.net/addgene:177014;

RRID:Addgene_177014), and pLIB-His$_6$-TEV-cyclin B1 (Addgene plasmid #177012; http://n2t.net/addgene:177012; RRID:Addgene_177012), deposited by A. Musacchio (*49*), were each used for preparation of baculoviruses in Sf9 cells. For expression of CDK1, Hi5 cultures were co-infected with baculoviruses encoding CDK1 and scCAK1, thereby ensuring that the CDK1 T-loop would be phosphorylated, at dilutions of 1:20 and 1:40, respectively. For expression of cyclin B1, Hi5 cultures were infected with baculovirus encoding cyclin B1 at a ratio of 1:20. Cultures were incubated and harvested as described. The cells were washed with phosphate buffered saline (PBS) before being snap frozen in liquid nitrogen for storage at -80 ˚C.

CDK1 pellet was resuspended in purification buffer C (50 mM HEPES pH 7.4, 200 mM NaCl, 5 % (v/v) glycerol, 2 mM tris(2-carboxyethyl)phosphine [TCEP]) supplemented with protease inhibitors and DNase I. The lysate was clarified as described, then passed through a 1.22 µm filter before incubating with equilibrated Glutathione Sepharose 4 Fast Flow beads (Cytiva) for 90 minutes at 4 ˚C. The beads were washed with purification buffer C. Elution was performed by incubating the beads with PreScission protease (Cytiva), at a ratio of 1 U protease per 0.1 mg of expected protein, overnight at 4 ˚C. Eluted protein was concentrated and subjected to gel filtration using a Superdex 200 13/30 column (Cytiva), with purification buffer D (50 mM HEPES pH 7.4, 250 mM NaCl, 5 % (v/v) glycerol, 2 mM TCEP). Selected fractions were combined, concentrated, aliquotted, snap frozen in liquid nitrogen, and stored at -80 ˚C.

Cyclin B1 pellet was resuspended in purification buffer D supplemented with protease inhibitors, DNase I, and 15 mM imidazole. The lysate was clarified as described, then incubated with equilibrated HIS-Select nickel magnetic agarose beads (Sigma) for 60 minutes at 4 ˚C. The beads were washed with purification buffer D supplemented with 15 mM imidazole and eluted with purification buffer D supplemented with 250 mM imidazole. Eluted protein was concentrated and incubated with TEV protease (at a final protease concentration of 100 µg/ml) at 4 ˚C overnight to cleave the tag. Gel filtration was performed using a Superdex 200 HiLoad 16/600 column (Cytiva), with purification buffer D. Selected fractions were combined and incubated with equilibrated HIS-Select nickel magnetic agarose beads for 1 minute at 4 ˚C to remove the protease. The flow-through was collected and concentrated, aliquotted, snap frozen in liquid nitrogen, and stored at -80 ˚C.

To generate the CDK1-cyclin B1 complex, purified CDK1 and cyclin B1 were combined in a 1:1 molar ratio and incubated on ice for 2 hours. Gel filtration was then performed using a Superdex 200 Increase 10/300 column, with purification buffer D. Selected fractions were combined, concentrated to approximately 1.5 mg/ml, aliquotted, snap frozen in liquid nitrogen, and stored at -80 ˚C.

CDK11: The sequence encoding residues 357-795 of human CDK11B^{p110} was obtained as a synthetic gene, codon-optimised for insect cell expression (Twist Bioscience), and cloned into an insect cell expression vector of the 438-series, resulting in an N-terminal Strep tag (see Table S4 for primer sequences). Baculovirus was generated and used for protein expression in High Five (*Trichoplusia ni*) cells in the same way as for CAK complexes. Purification followed the same general protocol as for CAK complexes, except that only a single StrepTactin affinity step was performed and the tag was cleaved before gel filtration by incubation with TEV protease for 1 hr at 4 ˚C. Selected fractions from gel filtration were combined, concentrated, aliquotted, snap frozen in liquid nitrogen, and stored at -80 ˚C.

Cks1: The Cks1 expression plasmid was purchased from GenScript. The construct consisted of the sequence encoding residues 5-73 of human Cks1 (*50*), codon-optimised for expression in *E. coli,* cloned into the pGEX-6P-1 vector (resulting in an N-terminal GST tag). Expression was performed in 1 L *E. coli* Rosetta (DE3) pLysS cell culture as described. The pellet was resuspended in purification buffer B supplemented with protease inhibitors and DNaseI and lysed by sonication. The lysate was clarified by spinning as described, then incubated with equilibrated Glutathione Sepharose 4B beads (Cytiva) for 60 minutes at 4 ˚C. The beads were washed with purification buffer B and eluted with purification buffer B supplemented with 10 mM reduced glutathione. The eluted fractions were combined, concentrated, and exchanged into gel filtration buffer C using a HiPrep 26/10 desalting column to remove the reduced glutathione. Output protein was then incubated with PreScission protease, at a ratio of 2 U protease per 0.1 mg tagged protein, at 4 ˚C overnight to cleave the GST tag. After cleavage, the sample was incubated with equilibrated Glutathione Sepharose 4B beads for 60 minutes at 4 ˚C to remove the GST-tagged protease and free GST. The flow-through was then subjected to gel filtration using a Superdex 75 10/300 column; eluted fractions were combined, concentrated to 0.9 mg/ml, aliquotted, and snap frozen in liquid nitrogen for storage at -80 ˚C.

Sample preparation for cryo-EM

For the CAK-CDK2-cyclin A2 complexes, 12 µg CAK was combined with 9 µg CDK2-cyclin A2, made up to 50 µl with buffer (25 mM HEPES pH 7.5, 150 mM NaCl, 10 mM $MgCl_2$, with or without 0.5 mM AMP-PNP for nucleotide-bound and apo samples, respectively), and incubated for 15 minutes at RT before being placed on ice. For the CAK-CDK2 (ADP-nitrate) complex, 6 µg CAK was combined with 6.5 µg CDK2, made up to 25 µl with ADP-nitrate buffer (25 mM HEPES pH 7.5, 150 mM NaCl, 5 mM $Mg(NO_3)_2(H_2O)_6$, 1 mM ADP), and incubated for 15 minutes at RT before being placed on ice. For the CAK-CDK2 (ADP-AlF_x) complex, ADP-AlF_x buffer was first prepared by combining 25 mM HEPES pH 7.5, 150 mM NaCl, and 5 mM $MgCl_2$ with (in the following order) 30 mM NaF, 5 mM $AlCl_3$, and 1 mM ADP (mixing after each addition), then incubating this for 30 minutes at RT. To prepare the sample, 6 µg CAK was combined with 8.6 µg CDK2, made up to 25 µl with ADP-AlF_x buffer, and incubated for 30 minutes at RT before being placed on ice. For the CAK-CDK1-cyclin B1 complex, 12 µg CAK was combined with 9 µg CDK1-cyclin B1, made up to 50 µl with buffer (25 mM HEPES pH 7.5, 150 mM NaCl, 10 mM $MgCl_2$, 0.5 mM AMP-PNP), and incubated for 15 minutes at RT before being placed on ice. For the CAK-CDK11 complex, 12 µg CAK was combined with 13.6 µg CDK11, made up to 50 µl with buffer (20 mM HEPES pH 7.9, 200 mM KCl, 2 mM $MgCl_2$, 2 mM ADP), and incubated for 15 minutes at RT before being placed on ice.

Cryo-EM grids were prepared by applying 4 µl sample to UltrAuFoil R1.2/1.3 holey gold grids (Quantifoil Microtools) that had been plasma cleaned for 50 s using a Tergeo plasma cleaner (PIE Scientific). Grids were blotted for 1, 1.5, 2, or 2.5 seconds (for CAK-CDK2(-cyclin A2) and CAK-CDK1-cyclin B1 complexes) or 1.5, 2, 2.5, or 3 seconds (for CAK-CDK11 due to increased sample viscosity as a result of higher glycerol content) at 5 ˚C and 100 % humidity using a Vitrobot Mark IV (Thermo Fisher Scientific) before plunge-freezing in liquid ethane at liquid nitrogen temperature. Grids were then clipped into autogrid cartridges.

Cryo-EM grid screening and data collection

CAK-CDK2-cyclin A2-AMP-PNP: Several grids were screened across multiple sessions on a 300 kV Krios microscope (Thermo Fisher Scientific) equipped with a K3 detector (Gatan) and a BioQuantum energy filter (Gatan) at the London Cryo-EM Consortium (LonCEM) facility at The Francis Crick Institute, London or on a 200 kV Glacios microscope (Thermo Fisher

Scientific) equipped with Falcon 4i detector (Thermo Fisher Scientific) at the Institute of Cancer Research (ICR), London. During screening on the LonCEM Krios, eight grids were loaded, atlases were generated, and a few test exposures were taken across different areas of selected grids. Two grids were chosen for data collection, which was performed on a 300 kV Krios microscope equipped with K3 detector and BioQuantum energy filter at the electron Bio-Imaging Centre (eBIC) at Diamond Light Source, Oxfordshire. Imaging was performed at a nominal magnification of 130,000, giving a pixel size of 0.645 Å/pixel, a total electron exposure of 50 $e^-/Å^2$, and with a C2 aperture of 50 µm, yielding a total of 33,998 movies in TIFF format from across both grids. Grids that were screened on the ICR Glacios did not end up progressing to large collection but were subjected to small data collections of approx. 2,000-4,000 movies. These were processed in cryoSPARC live (*51*) to assess sample quality and generate a preliminary reconstruction that would later serve as the initial reference for processing of the data collected at eBIC.

Apo-CAK-CDK2-cyclin A2: For screening, multiple grids were loaded on the LonCEM Krios, atlases were generated, and a few test shots were taken from the most promising grids. A large dataset was then collected from the best grid on a 300 kV Krios microscope equipped with Falcon 4i detector and Selectris X energy filter (Thermo Fisher Scientific) at eBIC. Data were collected at a nominal magnification of 215,000, giving a pixel size of 0.576 Å/pixel, and a total dose of 70 $e^-/Å^2$, yielding 10,934 movies in EER format.

CAK-CDK2 (ADP-nitrate): Grids were screened on the LonCEM Krios in the same way as for the CAK-CDK2-cyclin A2 complexes. A single grid was selected and progressed immediately to overnight collection on the same microscope. Imaging was performed at a nominal magnification of 165,000, giving a pixel size of 0.51 Å/pixel, a total dose of 60 $e^-/Å^2$, and with a GIF slit width of 20 eV, yielding a total of 13,913 movies in TIFF format.

CAK-CDK2 (ADP-AlF$_x$): Grids were screened on the ICR Glacios. Seven grids were loaded and atlases generated before small datasets were collected from selected grids (approx. 200-300 movies), which were assessed by cryoSPARC live processing as above. A large dataset was collected from the best grid on a 300 kV Krios microscope equipped with K3 detector at eBIC. Data were collected at a nominal magnification of 165,000, giving a pixel size of 0.513 Å/pixel, and a total dose of 70 $e^-/Å^2$, yielding 23,694 movies in TIFF format.

CAK-CDK1-cyclin B1: Grids were screened using a similar strategy on the ICR Glacios. A single grid was chosen and used for collection of two separate datasets on the same microscope. Data were collected at a nominal magnification of 165,000, giving a pixel size of 0.7 Å/pixel, and a total dose of 60 $e^-/Å^2$, yielding a total of 14,030 movies in EER format across the two datasets.

CAK-CDK11: Grids were screened on the LonCEM Krios in the same way as for the CAK-CDK2-cyclin A2 and CAK-CDK2 (ADP-nitrate) complexes. Two grids were selected and queued for overnight collection on the same microscope using EPU MultiGrid (Thermo Fisher Scientific). Imaging was performed at a nominal magnification of 165,000, giving a pixel size of 0.51 Å/pixel, a total dose of 70 $e^-/Å^2$, and with a GIF slit width of 20 eV, yielding a total of 10,274 movies in TIFF format from across both grids.

Cryo-EM data processing

CAK-CDK2-cyclin A2-AMP-PNP: Datasets from two grids were pre-processed separately in cryoSPARC (*51*) before being combined and further processed in RELION 4.0 (*52*, *53*). Data

were first processed in cryoSPARC live, during which movies were binned 2x during motion correction. Particles were picked using the in-built blob picker (both elliptical and circular blobs), with diameter 90-110 Å, and extracted using a box size of 160 x 160 pixels. Picked particles were classified into 50 2D classes using default parameters and selected classes were refined in 3D, using a previous reconstruction from an earlier screening dataset as an initial reference. In the main cryoSPARC interface, selected 2D classes from the live session were re-extracted from accepted micrographs to improve particle centring and duplicate particles were removed. The particle set was exported and converted to a .star file format compatible with RELION using the csparc2star.py program within the PYEM package (*54*).

In RELION 4.0, accepted movies from the two datasets were separately motion corrected while binning 2x, giving a pixel size of 1.29 Å/pixel. Exported particles from cryoSPARC were extracted from motion-corrected movies using a box size of 160 x 160 pixels and separately 3D refined using an initial angular sampling of 7.5 degrees. Refined particles were re-extracted using a larger box size of 196 x 196 pixels, then particles from the two datasets were combined and refined again before being classified into four classes by alignment-free 3D classification ($\tau = 16$, mask diameter 140 Å). The best class was selected, refined, and subjected to Bayesian polishing (performed separately for the two sets of movies after training each on 5,000 particles) with re-windowing and re-scaling to a box size of 234 x 234 pixels and a pixel size of 1.09154 Å/pixel. After another round of 3D refinement followed by CTF refinement of per-micrograph astigmatism, the particle set was refined a final time and post-processed using a tight mask to generate a reconstruction of the full complex at 2.5 Å. A local refinement of the final particle set was additionally performed using a mask encompassing only the CDK2-cyclin A2 subcomplex. This yielded a reconstruction at 2.6 Å with improved density for the CDK7 C-terminal RxL motif bound in the cyclin A2 hydrophobic patch. The data processing workflow for this complex is outlined in fig. S19.

Apo-CAK-CDK2-cyclin A2: Data were initially processed in cryoSPARC, as for each of the AMP-PNP-bound CAK-CDK2-cyclin A2 datasets, before selected particle picks were exported to RELION 5.0. In RELION, movies were motion corrected while binning 2x, giving a pixel size of 1.152 Å/pixel. Particle extraction, re-extraction, 3D refinement, and alignment-free 3D classification were performed as for the AMP-PNP-bound complex. The best class was selected, refined, and subjected to CTF refinement of beamtilt, trefoil, and fourth-order aberrations, followed by Bayesian polishing (after training the polishing on 5,000 particles) without re-windowing or re-scaling. The polished particles were refined a final time and post-processed to generate a reconstruction at 2.6 Å. The data processing workflow for this complex is outlined in fig. S20.

CAK-CDK2 (ADP-nitrate): Data were first processed in cryoSPARC live, with blob picking and 2D classification performed as for each of the CAK-CDK2-cyclin A2 datasets. Selected live classes were subjected to homogeneous refinement in the main cryoSPARC interface, using a reconstruction generated from a now-defunct dataset of CAK-CDK2 bound to AMP-PNP (collected on the ICR Glacios) as an initial reference. Refinement produced an anisotropic reconstruction due to preferred orientation of the live-picked particles. To maximise particle numbers and isolate rare views to improve the isotropy of the reconstruction, two additional rounds of template-based picking were performed in cryoSPARC – first using selected live classes as templates, then using projections derived from the reconstruction as templates. Template-based picks were extracted from accepted micrographs using a box size of 160 x 160 pixels and re-classified in 2D (200 classes, 120 Å circular mask, 120 iterations, batch size per class 200). To remove ice contamination, two additional rounds of 2D classification were

performed for each template-based picking strategy, whereby all non-ice classes were selected and re-classified. The best 2D classes from the final four rounds of classification were selected and combined, alongside selected classes from the live classification, before duplicate particles were removed. After re-extraction from accepted micrographs to improve particle centring, a final 2D classification (100 classes) was performed to generate the cleanest particle set for export to RELION 4.0.

The initial motion correction, extraction, refinement, and re-extraction strategy performed in RELION was the same as for the CAK-CDK2-cyclin A2 complexes. Duplicate particles were then removed and the remaining particles were classified into four 3D classes; the three best classes were selected and refined before a second round of 3D classification. The best class was selected, refined, and subjected to two rounds of CTF refinement (of per-micrograph astigmatism followed by beamtilt, trefoil, and fourth-order aberrations) before Bayesian polishing (after training the polishing on 5,000 particles) without re-windowing or re-scaling. Polished particles were refined again and post-processed to yield a reconstruction at 3.1 Å. Up to this point, due to pixel size calibration issues, the data processing had been performed using a binned pixel size of 1.04 Å, so the final refinement job was re-run using an input .star file in which the defocus values had been corrected according to a pixel size correction factor and using a reference and mask scaled to the correct pixel size of 1.02 Å. This was post-processed to yield the final reconstruction at 3.1 Å global resolution. The data processing workflow for this complex is outlined in fig. S21.

CAK-CDK2 (ADP-AlF$_x$): Data were initially processed in cryoSPARC live, then followed by an additional two rounds of template-based picking in the main cryoSPARC interface, as for the ADP-nitrate dataset. Template-based picks were extracted from accepted micrographs using a box size of 160 x 160 pixels, binned to 80 x 80 pixels, then each subjected to a round of 2D classification (200 classes). Live-picked particles were re-extracted using the same down-sampled box size and also re-classified. Selected particles from the three classifications were combined, duplicates removed, and extracted from accepted micrographs using a box size of 160 x 160 pixels (without down-sampling). Duplicates were re-removed and a final 2D classification (100 classes) was performed before export to RELION 5.0.

In RELION, movies were motion corrected while binning 2x, giving a pixel size of 1.026 Å/pixel. The data were then subjected to the same extraction, re-extraction, refinement, and 3D classification strategy as the other datasets (with a duplicates removal step performed before the second extraction). The best three classes were carried forward into CTF refinement of per-micrograph astigmatism before Bayesian polishing (after training the polishing on 5,000 particles) without re-windowing or re-scaling. Polished particles were subjected to another round of 3D classification to select the highest-quality particle subset for a final round of refinement and post-processing, yielding a reconstruction at 2.4 Å global resolution. The data processing workflow for this complex is outlined in fig. S22.

CAK-CDK1-cyclin B1: The two datasets were pre-processed separately in cryoSPARC. For each dataset, data were initially processed in cryoSPARC live before a further two rounds of template-based picking in the main cryoSPARC interface, as for the CAK-CDK2 datasets, due to issues of preferred orientation. Template-based picks were extracted from accepted micrographs using a box size of 160 x 160 pixels, binned to 80 x 80 pixels, then each subjected to a round of 2D classification (200 classes). Selected classes were combined and duplicate particles removed before re-extraction from accepted micrographs using a box size of 160 x 160 pixels. Re-extracted particles were combined with selected blob-picked particles from the

live session; this particle set was then classified into 100 2D classes and selected particles were subjected to a final round of re-extraction to improve particle centring. Duplicates were re-removed and particles were exported to RELION, resulting in two particle sets for RELION 5.0 processing.

In RELION 5.0, accepted movies from the two datasets were separately motion corrected while binning 2x, giving a pixel size of 1.4 Å/pixel. Each set of exported particles from cryoSPARC was extracted from their respective motion-corrected movies, using a box size of 160 x 160 pixels, before being combined and 3D refined. Refined particles were subjected to CTF refinement, followed by 3D refinement, Bayesian polishing (performed separately for the two sets of movies and extracting with a larger box size of 170 x 170 pixels), and another round of 3D refinement. At this stage, a reconstruction at 3.1 Å global resolution could be obtained – however, due to flexibility, cyclin B1 was not very well resolved. Alignment-free 3D classification into four classes was therefore performed, with the aim of isolating a subset of particles with better density in this region. Selection of the best class was followed by a final round of 3D refinement and post-processing to generate a reconstruction at 3.4 Å global resolution with improved map quality. The data processing workflow for this complex is outlined in fig. S23.

CAK-CDK11: Despite increasing the blotting time for sample preparation of this complex, the increased glycerol content resulted in thick ice across both grids imaged. The two datasets were processed separately in cryoSPARC live according to the usual protocol, during which movies with too-thick ice were excluded. Due to poor quality, processing of one dataset was terminated early, resulting in 1,271 accepted movies from a single dataset being taken forward into downstream processing. In the main cryoSPARC interface, two re-classifications of all live-picked particles, into 50 or 100 classes, and a 25-class re-classification of the best live-picked classes were performed to isolate further views. Selected classes from these three classifications were merged and duplicates removed before progressing into RELION 5.0. In RELION, exported particles were extracted from accepted motion-corrected movies, 3D refined, then classified into four 3D classes with Blush regularisation (*55*). The best particle subset was selected, re-classified into four 3D classes with Blush regularisation to further clean up the data, then subjected to CTF refinement of beamtilt and trefoil before a final refinement. Post-processing yielded a final reconstruction at 3.5 Å global resolution. The data processing workflow for the accepted dataset is outlined in fig. S24.

<u>Collection and processing of small cryo-EM datasets for mutant CAK complexes</u>

Samples for cryo-EM were prepared by diluting each mutant in buffer (20 mM HEPES pH 7.9, 200 mM KCl, 2 mM $MgCl_2$) to a final concentration of 0.4 mg/ml and supplementing with 2 mM AMP-PNP. 4 µl sample was applied to cryo-EM grids and blotted for 1.5 or 2 s (two grids for each mutant) before plunge freezing as described. Small datasets of approximately 1,000-2,000 micrographs were collected on the ICR Glacios using a nominal magnification of 165,000, a pixel size of 0.7 Å/pixel, an energy filter slit width of 10 eV, and a total dose of 60 $e^-/Å^2$. Each dataset was processed in cryoSPARC live as for the large datasets, before re-classification of the blob-picked particles from the live session into 100 2D classes. Selected particles were then subjected to homogeneous refinement in cryoSPARC using a previous CAK reconstruction as a reference.

<u>Atomic model building and refinement</u>

A model of the CAK-CDK2-cyclin A2-AMP-PNP complex was built first and later served as the initial model for building of the remaining complexes. An initial model of CAK-CDK2-

cyclin A2 was generated by rigid-body fitting of our previous high-resolution cryo-EM structure of CAK-ATPγS (PDB 8P6Y) (*30*) and an existing crystal structure of CDK2-cyclin A bound to an inhibitory peptide in the cyclin A hydrophobic patch (chains C, D, and F of PDB 1OKV) (*56*) into the full-complex reconstruction in UCSF ChimeraX (*57*). This was then edited by iterative rounds of manual model building in COOT (*58*) and real-space refinement in PHENIX (*59*). AMP-PNP ligands were imported from the internal COOT monomer library and manually real-space refined into the density in COOT. Water molecules were manually modelled in COOT where their presence was supported by clear density and the existence of nearby hydrogen bonding partner(s). Any water molecules with a Q-score (*60*) below 0.70 were excluded. After PHENIX real-space refinement of the model in the full-complex reconstruction, the model was real-space refined in the locally refined reconstruction to improve the accuracy of modelling of the CDK7 C-terminal peptide. The peptide was then copied back into the output model from real-space refinement in the full map, which was then real-space refined again with reference restraints on the peptide.

Initial models of the apo-CAK-CDK2-cyclin A2 complex and each of the CAK-CDK2 complexes were generated by fitting of the revelant chains of the CAK-CDK2-cyclin A2-AMP-PNP model into the post-processed maps in UCSF ChimeraX. An initial model of the CAK-CDK1-cyclin B1 complex was produced by rigid-body fitting of the CAK subunit chains from the CAK-CDK2-cyclin A2-AMP-PNP model and the CDK1 and cyclin B1 chains from a published crystal structure of the Cks2-CDK1-cyclin B complex (PDB 5HQ0) (*61*) into the map in UCSF ChimeraX. For the CAK-CDK11 complex, an initial model was generated by rigid-body fitting of the CDK7, cyclin H, and MAT1 chains from the CAK-CDK2-cyclin A2-AMP-PNP model and the CDK11 chain from the AlphaFold3-predicted model of the CAK-CDK11 complex into the post-processed map in UCSF ChimeraX. For all complexes, this was followed by manual rebuilding in COOT, during which ADP ligands were imported from the internal COOT monomer library as necessary. Manual adjustment of the protein chains and modelling of water molecules (performed according to the outlined criteria) was carried out alongside iterative rounds of real-space refinement in PHENIX. A final round of real-space refinement generated the final version of each model. Poor density in the active sites of the CAK-CDK2 complexes meant that the nitrate and AlF_x ligands could not be modelled.

The atomic models were validated by the comprehensive validation tool within PHENIX (*59*). The resulting statistics are shown in Tables S1 to S3.

Calculation of the surface area buried by the interaction of the CDK2 T-loop with cyclin A2 in the CAK-CDK2-cyclin A2-AMP-PNP complex was performed using the PDBe PISA server (*62*).

AlphaFold structure prediction

For structure prediction of CAK-CDK activation complexes, sequences for human CDK7 (full-length), cyclin H (full-length), MAT1 (residues 220-309), and various CDKs (full-length) were input into the online AlphaFold3 server (alphafoldserver.com) (*24*). For prediction of CDK7 RxL-cyclin interactions, full-length sequences for human cyclin B1, D1, E1, or F alongside the C-terminal 20 residues of human CDK7 were input into the server. For each prediction, the first output (*model_0.cif) was used for generation of model figures and PAE plots.

Pull-down assays

WT CAK was diluted in binding buffer (25 mM HEPES pH 7.5, 150 mM NaCl, 10 mM $MgCl_2$, 500 µM AMP-PNP) to a concentration of 0.1 mg/ml. Equilibrated StrepTactin 4Flow beads

(IBA) were incubated for 45 minutes at 4 ˚C with 10 µg CAK (which has an N-terminal Strep tag on CDK7), or binding buffer for negative controls. The beads were washed with binding buffer before incubating for 15 minutes at RT with 20 µg CDK2-cyclin A2 (at a concentration of 0.2 mg/ml in binding buffer) or 15 µg CDK2 (at a concentration of 0.15 mg/ml in binding buffer). The beads were washed with binding buffer and eluted with binding buffer supplemented with 10 mM desthiobiotin. 20 µl samples were analysed by SDS-PAGE using NuPAGE 4-12% Bis-Tris gels (Thermo Fisher Scientific). Gels were stained using Coomassie InstantBlue gel stain (Abcam) and imaged using the ChemiDoc imaging system (Bio-Rad). The intensity of each CDK2 band relative to the corresponding CDK7 band in eluted CDK2- and CDK2-cyclin A2-containing complexes was quantified using ImageLab 6.0.1 (Bio-Rad). Relative CDK2 band intensities were plotted as the mean and standard deviation of N = 3 biologically independent replicates and analysed by Welch's two-tailed t test using GraphPad Prism (v10.5.0).

In-vitro kinase assays

100 nM CAK was combined with 10 µM substrate and 2 mM ATP in a total volume of 100 µl in assay buffer A (25 mM HEPES pH 7.9, 150 mM KCl, 9 mM $MgCl_2$, 2.5 % glycerol, 5 mM BME) for assays with CDK2-cyclin A2 and $CDK2^{I209R}$ as substrates, or assay buffer B (as buffer A but with 5 mM $MgCl_2$) for all other assays. For assays assessing the impact of Cks1 on the activity of the CAK towards CDK2, Cks1 was additionally included in the reaction at a molar ratio of 0:1, 1:1, or 2:1 relative to CDK2. Reactions were incubated for 1 or 2 hr at RT and samples were taken at 0 hr and then every 0.5 hr thereafter, diluted 1:20 in assay buffer, and quenched by mixing with SDS-PAGE loading dye. For all experiments, two biologically independent sets of experiments using different protein preparations were performed, each with N = 3 technical replicates. 10 µl samples were subjected to SDS-PAGE using NuPAGE 4-12% Bis-Tris gels, followed by transfer to 0.2 µm nitrocellulose membranes using the Trans-Blot Turbo Transfer System (Bio-Rad) for Western blotting. Successful blotting was confirmed by inclusion of a control sample on each blot as needed.

Membranes were stained using Ponceau S staining solution (Thermo Fisher Scientific) and imaged using the ChemiDoc imaging system to obtain loading controls. Membranes were destained by washing in PBST (1X PBS supplemented with 0.1 % v/v Tween 20), then blocked in 5 % BSA/PBST (PBST supplemented with 5 % w/v bovine serum albumin [BSA]) at 4 ˚C overnight. Blotting was performed for 1 hr at RT using a rabbit anti-phospho-CDK2 (Thr160) primary antibody (Cell Signaling Technology, cat. no. 2561, RRID:AB_2078685) diluted 1:1000 in 5 % BSA/PBST. Membranes were washed in PBST, then incubated for 1 hr at RT with a goat anti-rabbit secondary antibody conjugated to IRDye® 800CW (LICORbio, cat. no. 926-32211, RRID:AB_621843) diluted 1:20,000 in 5 % BSA/PBST. After washing in PBST, membranes were imaged using the Odyssey CLx imaging system (LICORbio), using the 700 and 800 nm channels at laser intensity 4. The signal from the 800 nm channel was used for quantification and figure preparation. Band intensities were quantified using ImageJ (*63*). Blot band intensities were divided by those of loading controls to account for loading differences. For assays in which the substrate was constant, values for each time point were normalised to the relevant 0-hr sample value. For assays in which the substrate was varied (i.e. those assessing the activity of the CAK towards WT vs. mutant CDK2 or monomeric vs. cyclin-bound CDK2), values were normalised using the control signal. Relative intensity values were then plotted as the mean and standard deviation of N = 3 technical replicates and analysed by two-way ANOVA and Tukey's or Šídák's multiple comparisons test using GraphPad Prism (v10.5.0).

Mass spectrometry

Triplicate CDK2 samples with and without phosphorylation by the CAK (approx. 16 µg) were adjusted with triethylammonium bicarbonate buffer (TEAB) at a final concentration of 100 mM. Proteins were reduced and alkylated with 5 mM TCEP and 10 mM iodoacetamide (IAA) simultaneously for 60 min in the dark and digested overnight with trypsin at a final concentration of 50 ng/µL (Pierce). The samples were dried and peptides were cleaned up with desalting spin columns (Pierce) according to manufacturer's instructions.

Liquid chromatography-mass spectrometry (LC-MS) analysis was performed on a Vanquish Neo UHPLC system coupled with an Orbitrap Ascend Mass Spectrometer (Thermo Fisher Scientific). Peptides were reconstituted in 30 µL 0.1% TFA and 2 µL were loaded onto a PepMap 100, C18, 5 µm, 0.3×5 mm, 1500 bar, (Thermo Scientific cat. no. 174500) trapping column following elution in the analytical column which was attached to a Nanospray Flex ion source via a stainless-steel emitter (Thermo Scientific, cat. no. ES542). Peptides were then subjected to a gradient elution on a capillary column (Waters, nanoE MZ PST BEH130 C18, 1.7 µm, 75 µm × 250 mm). Mobile phase A was 0.1% formic acid and mobile phase B was 80% acetonitrile, 0.1% formic acid. The separation method at a flow rate of 300 nL/min was an 80 min gradient from 5%-35% B. Precursors between 375-1,500 m/z and charge states 2-7 were selected at 120,000 resolution in the top speed mode in 3 sec and isolated for HCD fragmentation (collision energy 32%) with quadrupole isolation width 0.7 Th, Orbitrap detection with 45,000 resolution and 91 ms Maximum Injection Time. Targeted MS precursors were dynamically excluded from further isolation and activation for 45 seconds with 10 ppm mass tolerance.

Peptide and protein identification was conducted in Proteome Discoverer 3.0 (Thermo Fisher Scientific) with the Sequest HT search engine. Precursor and fragment mass tolerances were 20 ppm and 0.02 Da respectively with a maximum of 2 trypsin missed cleavages allowed. Dynamic modifications included Carbamidomethyl at C, Oxidation of M, Deamidation of N/Q, and Phosphorylation of S/T/Y. Spectra were searched against a FASTA file containing reviewed *Homo sapiens* UniProt entries. Peptides were filtered at q-value < 0.01 using the Percolator node and target-decoy database search. Phosphorylation localization probabilities were estimated with the IMP-ptmRS node. Label-free peptide quantification was performed with the Minora Feature Detector and Feature Mapper nodes with RT alignment enabled, S/N greater than 5 and RT dependent normalization.

Nano-differential scanning fluorimetry (NanoDSF) thermal stability assay

Protein melting temperatures were determined using the Prometheus NT.48 instrument (NanoTemper Technologies, Munich, Germany). Purified protein was diluted in gel filtration buffer B (25 mM HEPES pH 7.9, 200 mM KCl, 5 % (v/v) glycerol, 2 mM $MgCl_2$, 5 mM BME). Standard nano-DSF grade capillaries (NanoTemper Technologies, Munich, Germany) were loaded with 12 µl sample at approx. 0.5 or 1.0 mg/ml. Excitation was set to 40 % or 20 % power, respectively, and a temperature gradient of 1 °C/min was applied from 20 to 95 °C. The intrinsic protein fluorescence at 330 and 350 nm was measured. Data were analysed using PR.ThermControl software v2.3.1 (NanoTemper Technologies, Munich, Germany) and visualised using GraphPad Prism (v10.5.0).

Data visualisation

Cryo-EM reconstructions and atomic models were visualised using UCSF ChimeraX (*57*) (version 1.8) and PyMOL (The PyMOL Molecular Graphics System, versions 2.5.2 and 3.0.5, Schrödinger, LLC) for data analysis, interpretation, and figure preparation. AlphaFold3 PAE

plots were visualised for figure preparation using the PAE Figure Editor tool at thecodingbiologist.com. Multiple sequence alignments were visualised for figure preparation using the ESPript 3.0 server (*64*).

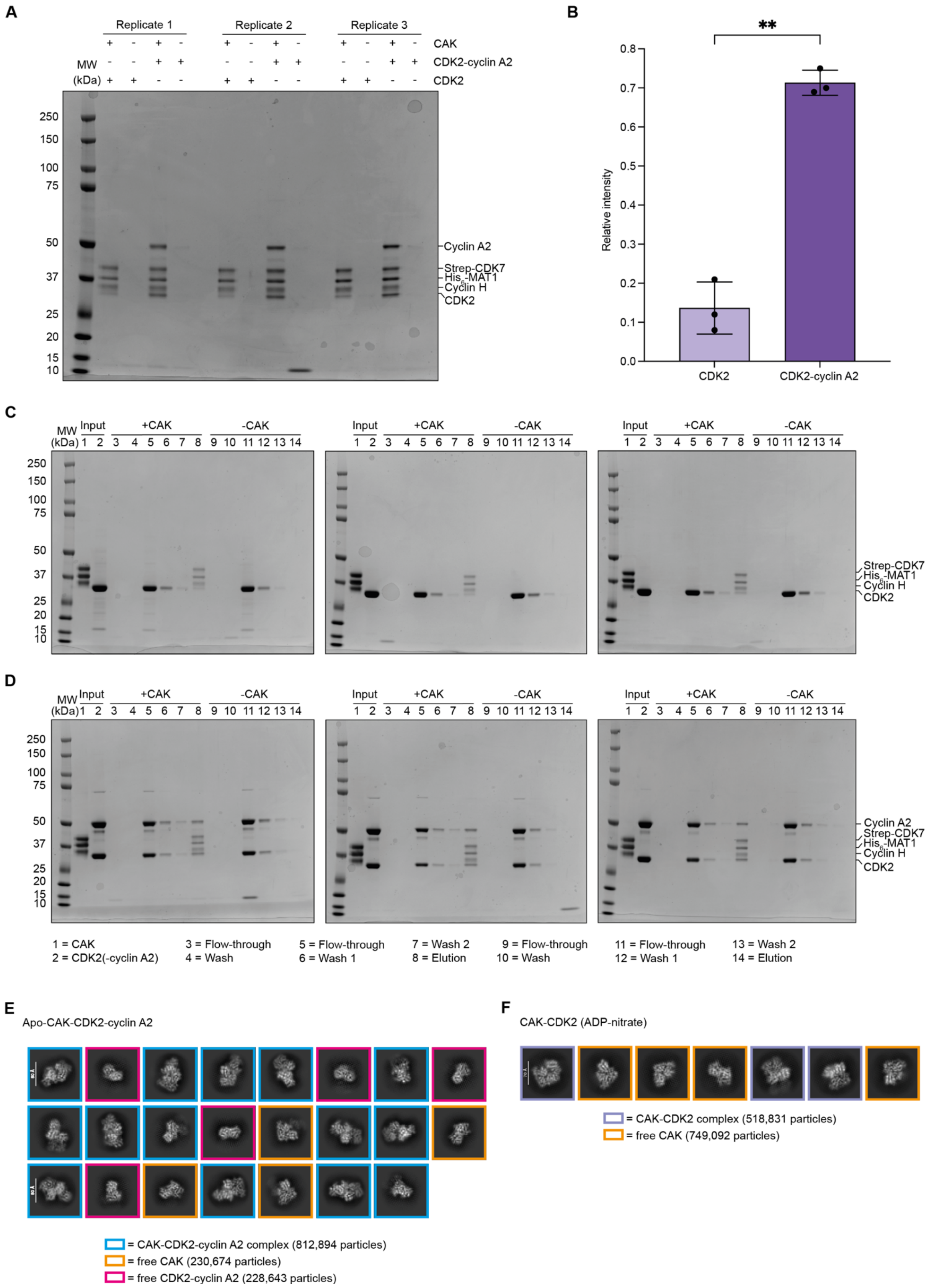

Fig. S1. Increased binding of CAK to CDK2 in the presence of bound cyclin. (A) SDS-PAGE analysis of eluted fractions from N = 3 biologically independent replicates of pull-down assays in which recombinant CAK complex (Strep-CDK7, His$_6$-MAT1, cyclin H) was immobilised on StrepTactin beads, incubated with either monomeric CDK2 or CDK2-cyclin

A2, and the resulting complexes eluted. For each replicate, CDK2 is recovered in roughly stoichiometric amounts in the presence of cyclin A2 and is sub-stoichiometric in the absence of bound cyclin. (**B**) Quantification of the intensity of the CDK2 band, relative to the CDK7 band, in eluted fractions of CDK2- and CDK2-cylin A2-containing complexes from the SDS-PAGE analysis shown in panel A. The plot is presented as the mean ± standard deviation of N = 3 biologically independent replicates. *P*-value obtained by Welch's two-tailed t test = 0.0011 (** = $P \leq 0.01$). (**C**, **D**) SDS-PAGE analysis of all fractions from all three biologically independent replicates of pull-down assays of CAK with CDK2 (C) and CDK2-cyclin A2 (D). (**E**, **F**) All good-quality classes arising from cryoSPARC live 2D classification of the apo-CAK-CDK2-cyclin A2 (E) and CAK-CDK2 (ADP-nitrate) (F) datasets. In the absence of bound cyclin, fewer assembled CAK-CDK2 complexes and a higher fraction of isolated components are present in the micrographs, resulting in substantially fewer good-quality classes of the full complex.

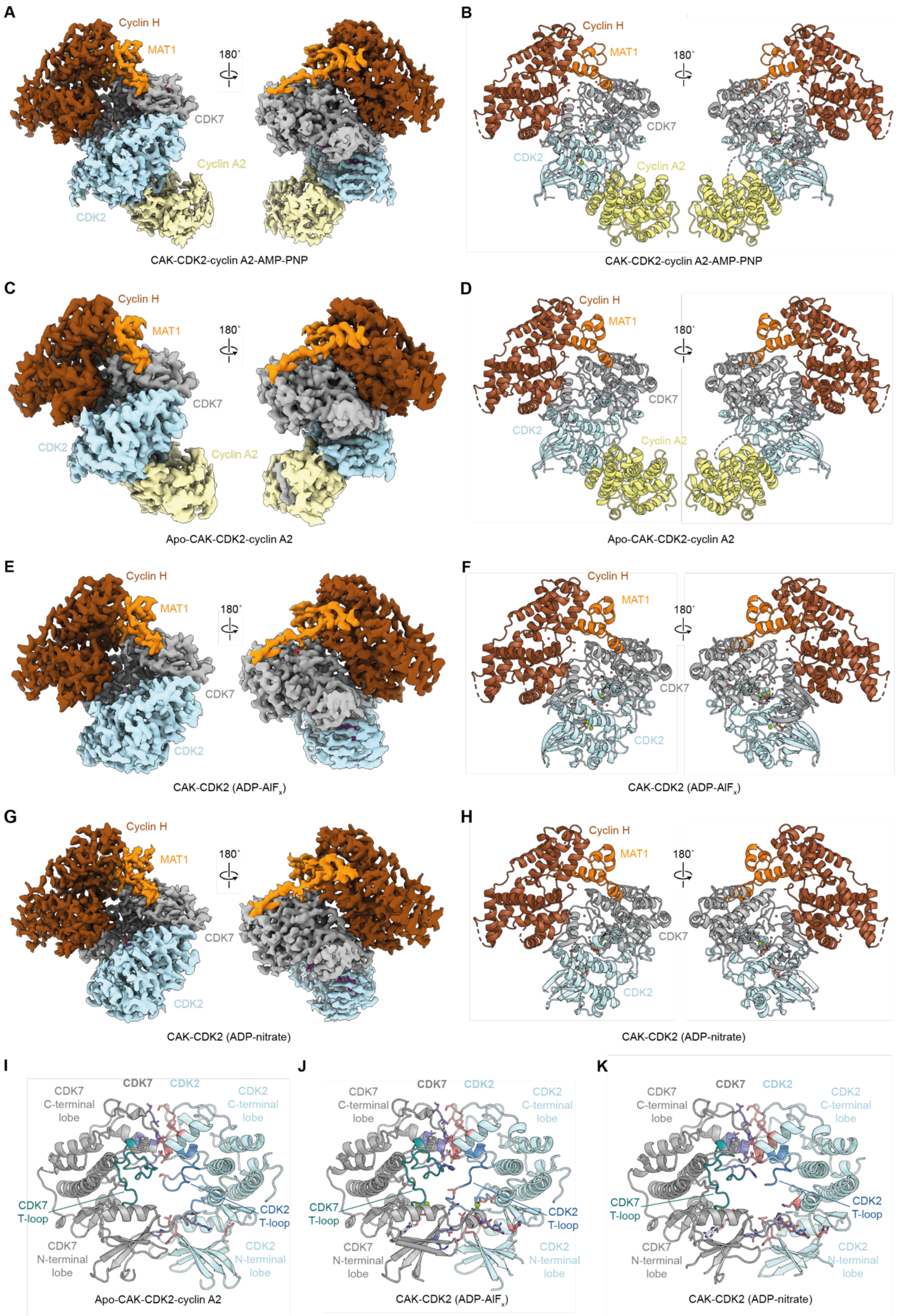

Fig. S2. Comparison of all cryo-EM structures of CAK-CDK2-cyclin A2 and CAK-CDK2 complexes. (**A**) Cryo-EM reconstruction of the CAK-CDK2-cyclin A2-AMP-PNP complex.

(**B**) Atomic model of the CAK-CDK2-cyclin A2-AMP-PNP complex. (**C**) Cryo-EM reconstruction of the apo-CAK-CDK2-cyclin A2 complex. (**D**) Atomic model of the apo-CAK-CDK2-cyclin A2 complex. (**E**) Cryo-EM reconstruction of the CAK-CDK2 (ADP-AlF_x) complex. (**F**) Atomic model of the CAK-CDK2 (ADP-AlF_x) complex. (**G**) Cryo-EM reconstruction of the CAK-CDK2 (ADP-nitrate) complex. (**H**) Atomic model of the CAK-CDK2 (ADP-nitrate) complex. (**I-K**) Close-up view of the CDK7-CDK2 interaction interface in the apo-CAK-CDK2-cyclin A2 (I), CAK-CDK2 (ADP-AlF_x) (J), and CAK-CDK2 (ADP-nitrate) (K) complexes (for clarity, only the two kinases are shown). Interacting residues of CDK7 and CDK2 are shown in purple and pink. To identify and visualise the interactions, any residues within a 3.4 Å distance of residues in the neighbouring subunit were initially selected. This was combined with visual inspection to exclude any ≤ 3.4 Å contacts that are not chemically meaningful and to additionally include longer-range hydrophobic contacts (≤ 4 Å).

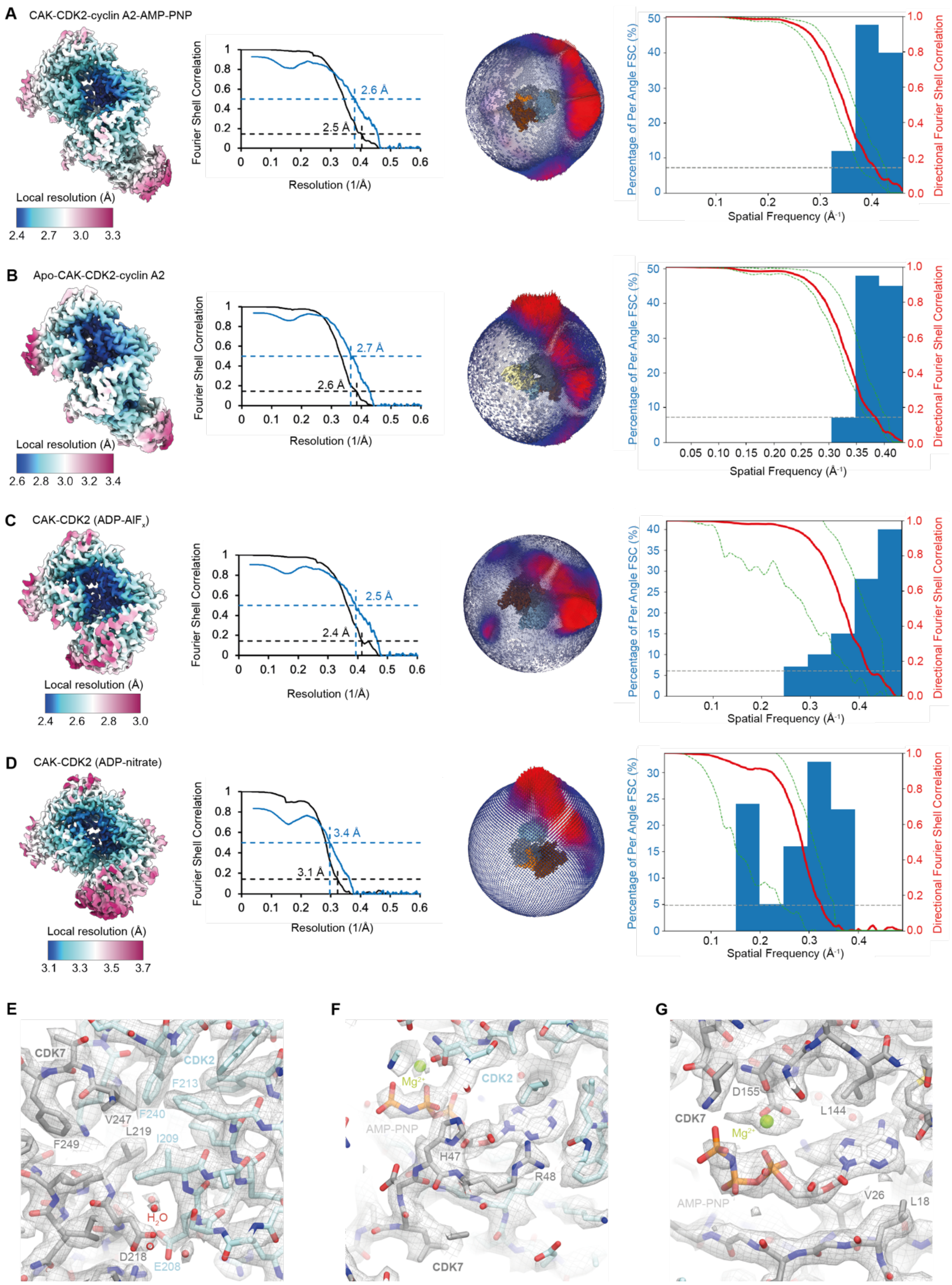

Fig. S3. Validation of cryo-EM structures of CAK-CDK2-cyclin A2 and CAK-CDK2 complexes. (**A-D**) From left to right: Cryo-EM reconstruction coloured by local resolution, FSC curves, particle orientation distribution plot, and 3D FSC calculation (*65*) for the CAK-CDK2-cyclin A2-AMP-PNP (A), apo-CAK-CDK2-cyclin A2 (B), CAK-CDK2 (ADP-AlF$_x$) (C), and CAK-CDK2 (ADP-nitrate) (D) complexes. Half-map FSC curves are shown in black and model vs. map FSC curves are shown in blue. Resolutions are estimated according to the

FSC = 0.143 criterion for half-maps, and the FSC = 0.5 criterion for model vs. map FSCs (*66*). Sphericity values of the reconstructions are given in Tables S1 and S2. (**E**) Map-model fit highlighting the cryo-EM density around the C-lobe kinase-kinase interaction interface in the CAK-CDK2-cyclin A2-AMP-PNP cryo-EM structure. Map contour level = 6 σ; carving radius = 2.5 Å. (**F**) Map-model fit highlighting the cryo-EM density around the N-lobe kinase-kinase interaction interface in the CAK-CDK2-cyclin A2-AMP-PNP cryo-EM structure. Map contour level = 5 σ; carving = 2.5 Å. (**G**) Map-model fit highlighting the cryo-EM density around the CDK7 active site in the CAK-CDK2-cyclin A2-AMP-PNP cryo-EM structure. Map contour level = 5.5 σ; carving = 20.0 Å.

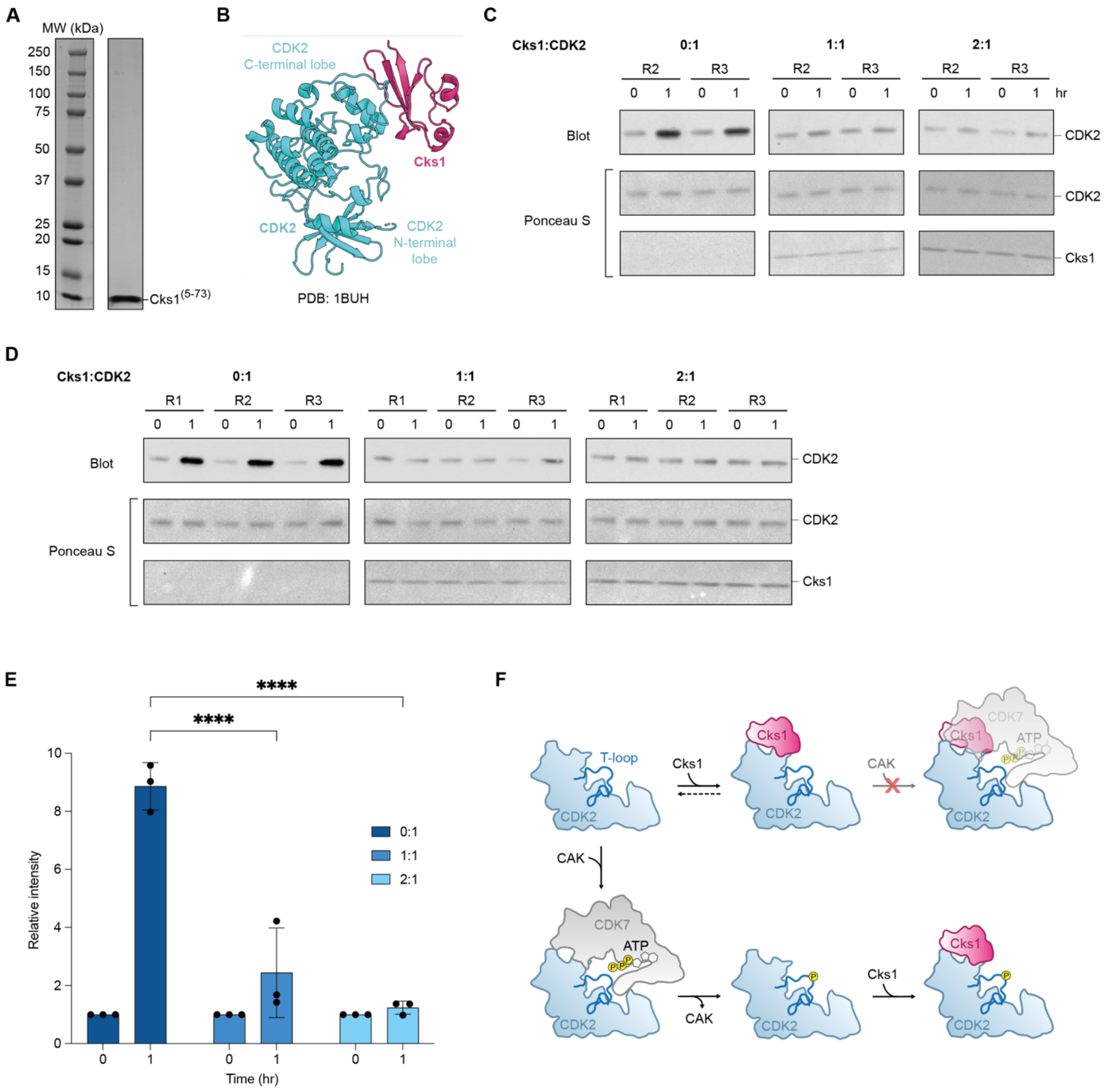

Fig. S4. Cks1 binding prevents phosphorylation of CDK2 by the CAK . (**A**) SDS-PAGE of purified Cks1 protein (residues 5-73). (**B**) Atomic model of the CDK2-Cks1 complex (PDB 1BUH) (*26*). (**C**, **D**) Western blots against phosphorylated CDK2 and Ponceau S-stained loading controls for kinase assays assessing the activity of the CAK towards CDK2 in the presence/absence of Cks1. Two biologically independent sets of experiments were performed, each with N = 3 technical replicates (R1, R2, and R3). The remaining two technical replicates from the set of experiments represented in Fig. 2B are shown in panel C; three technical replicates from the second set of experiments are shown in panel D. (**E**) Quantification of Western blot band intensities for the data shown in panel D, presented as the mean ± standard deviation of N = 3 technical replicates. P-values obtained by two-way ANOVA and Tukey's multiple comparisons test: 0:1 1 hr vs. 1:1 1 hr $P < 0.0001$; 0:1 1 hr vs. 2:1 1 hr $P < 0.0001$ (**** = $P \leq 0.0001$). (**F**) Cartoon outlining the order of events with respect to phosphorylation of, and Cks1 binding to, CDK2. Since CDK2 cannot bind both the CAK and Cks1 at the same time, phosphorylation by the CAK (represented by CDK7 only) must occur before Cks1 binding to CDK2. Cyclins are not shown for clarity.

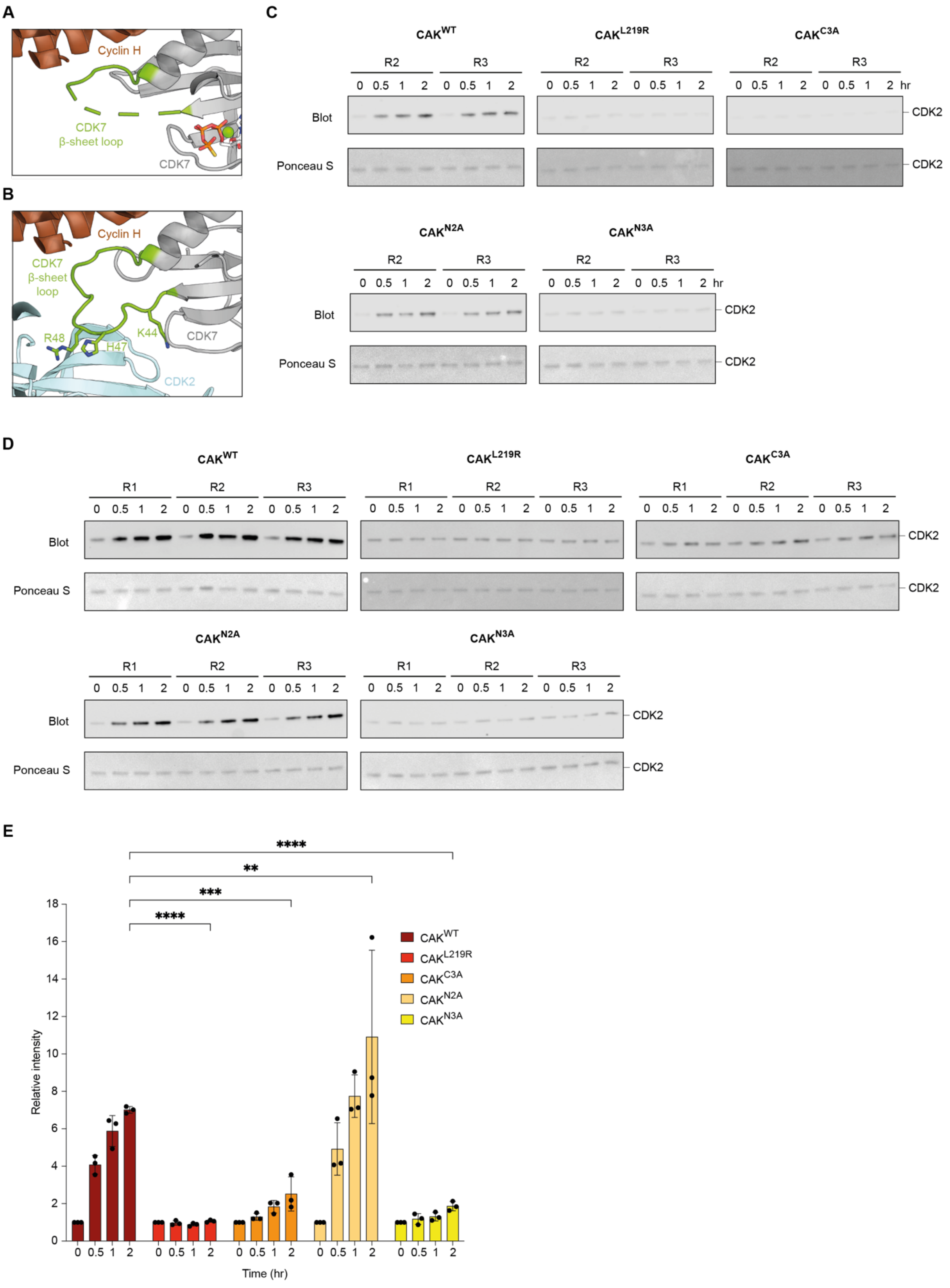

Fig. S5. Further analysis of the N- and C-terminal interface regions of the CAK-CDK2-cyclin A2 complex. (**A**) Close-up of free CAK (PDB 8P6Y) (*30*) showing that the CDK7 N-terminal loop is unstructured in the absence of substrate, as indicated by a dashed line. (**B**) Close-up of the apo-CAK-CDK2-cyclin A2 complex, in the same view as in panel A,

highlighting CDK7 loop residues K44A, H47, and R48. (**C**, **D**) Western blots against phosphorylated CDK2 and Ponceau S-stained loading controls for assays assessing the activity of CAK^{WT}, CAK^{L219R}, CAK^{C3A}, CAK^{N2A}, and CAK^{N3A} towards CDK2. Two biologically independent sets of experiments were performed, each with N = 3 technical replicates (R1, R2, and R3). The remaining two technical replicates from the set of experiments represented in Fig. 2F are shown in panel C; three technical replicates from the second set of experiments are shown in panel D. (**E**) Quantification of Western blot band intensities for the data shown in panel D, presented as the mean ± standard deviation of N = 3 technical replicates. *P*-values obtained by two-way ANOVA and Tukey's multiple comparisons test: CAK^{WT} 2 hr vs. CAK^{L219R} 2 hr $P < 0.0001$; CAK^{WT} 2 hr vs. CAK^{C3A} 2 hr $P = 0.0002$; CAK^{WT} 2 hr vs. CAK^{N2A} 2 hr $P = 0.0016$; CAK^{WT} 2 hr vs. CAK^{N3A} 2 hr $P < 0.0001$ (** = $P \leq 0.01$, *** = $P \leq 0.001$, **** = $P \leq 0.0001$).

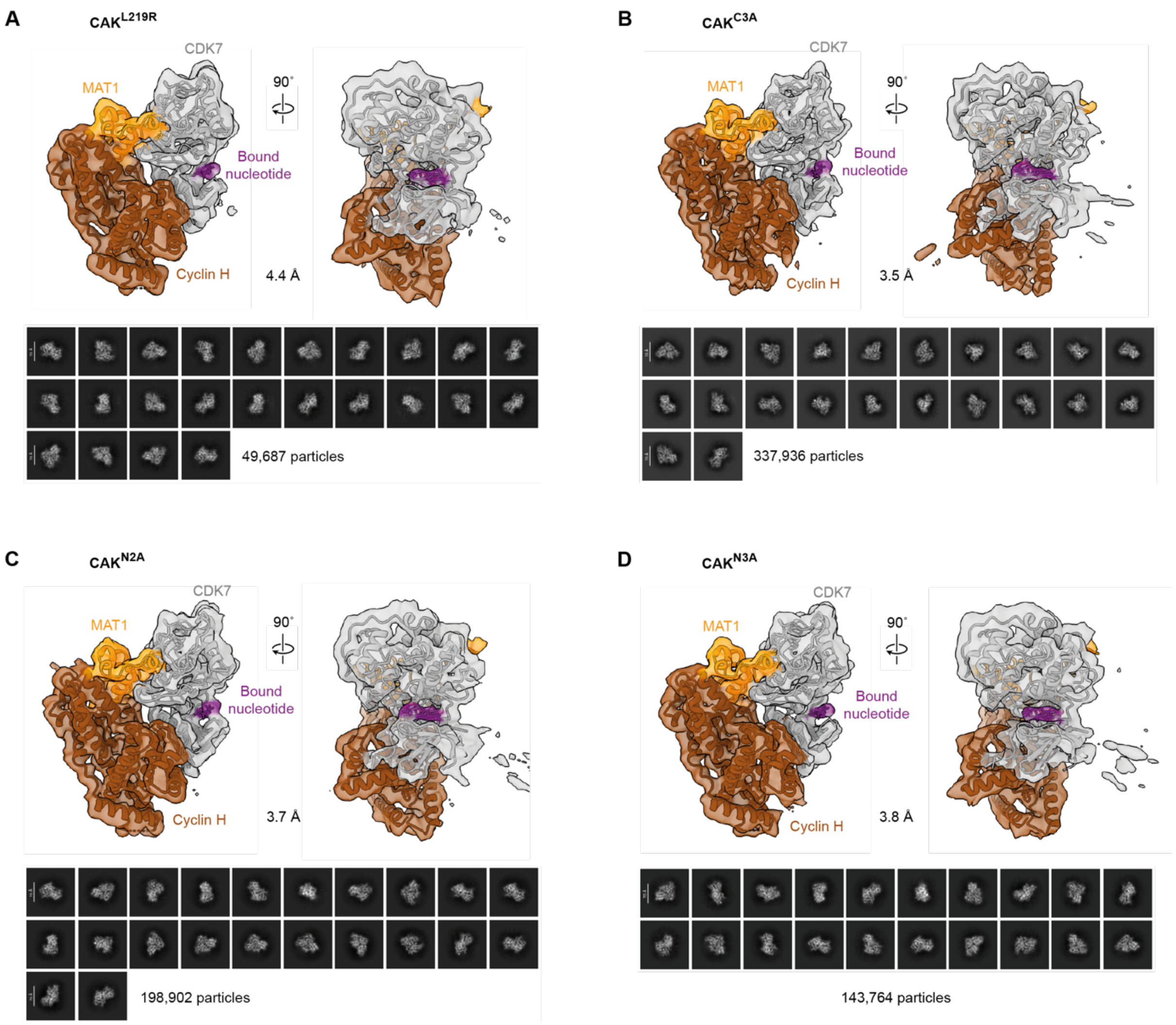

Fig. S6. Small cryo-EM data collections of CAK mutants. (**A-D**) 3D reconstruction (top) and all qood quality 2D classes (bottom) from small cryo-EM datasets of CAK^{L219R} (A), CAK^{C3A} (B), CAK^{N2A} (C), and CAK^{N3A} (D) complexes. An atomic model derived from a high-resolution cryo-EM map of the WT CAK complex bound to the nucleotide analogue ATPγS (PDB 8P6Y, (*30*)) is shown fitted into each 3D reconstruction. The 2D classes and the excellent fit of the model to the 3D reconstructions indicate the presence of folded CDK7 and confirm that all mutants can still bind nucleotides (AMP-PNP was used for the cryo-EM reconstructions) in their active site.

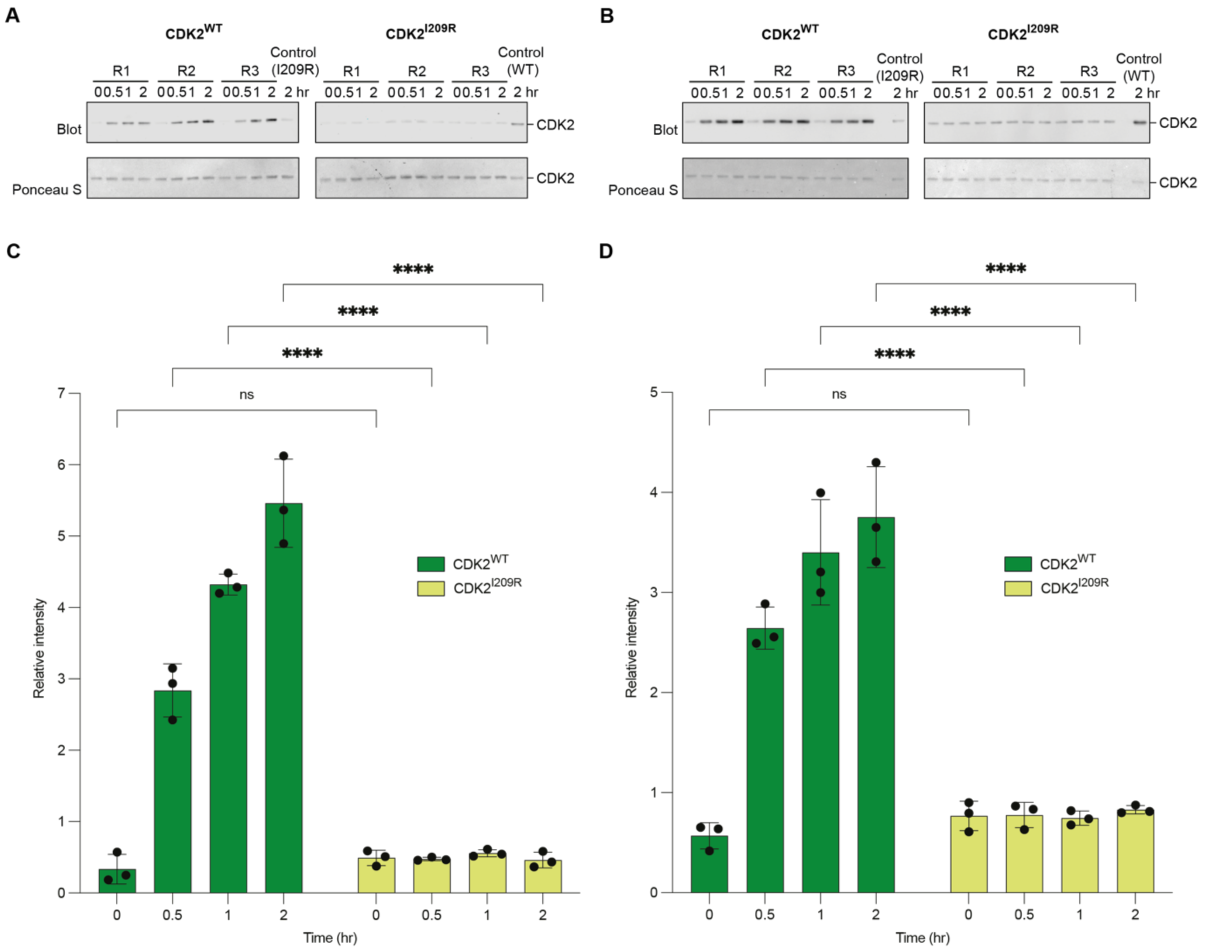

Fig. S7. In-vitro kinase assays assessing the activity of the CAK towards interface mutant CDK2. (**A**, **B**) Western blots against phosphorylated CDK2 and Ponceau S-stained loading controls for assays assessing the activity of the CAK towards $CDK2^{WT}$ (left) and $CDK2^{I209R}$ (right). Two biologically independent sets of experiments were performed (A, B), each with N = 3 technical replicates (R1, R2, and R3). (**C**) Quantification of Western blot band intensities for the data shown in panel A, presented as the mean ± standard deviation of N = 3 technical replicates. *P*-values obtained by two-way ANOVA and Šídák's multiple comparisons test: $CDK2^{WT}$ 0 hr vs. $CDK2^{I209R}$ 0 hr $P = 0.9358$; $CDK2^{WT}$ 0.5 hr vs. $CDK2^{I209R}$ 0.5 hr $P < 0.0001$; $CDK2^{WT}$ 1 hr vs. $CDK2^{I209R}$ 1 hr $P < 0.0001$; $CDK2^{WT}$ 2 hr vs. $CDK2^{I209R}$ 2 hr $P < 0.0001$. (ns = $P > 0.05$; **** = $P < 0.0001$). (**D**) Quantification of Western blot band intensities for the data shown in panel B, presented as the mean ± standard deviation of N = 3 technical replicates. *P*-values obtained by two-way ANOVA and Šídák's multiple comparisons test: $CDK2^{WT}$ 0 hr vs. $CDK2^{I209R}$ 0 hr $P = 0.8706$; $CDK2^{WT}$ 0.5 hr vs. $CDK2^{I209R}$ 0.5 hr $P < 0.0001$; $CDK2^{WT}$ 1 hr vs. $CDK2^{I209R}$ 1 hr $P < 0.0001$; $CDK2^{WT}$ 2 hr vs. $CDK2^{I209R}$ 2 hr $P < 0.0001$.

A

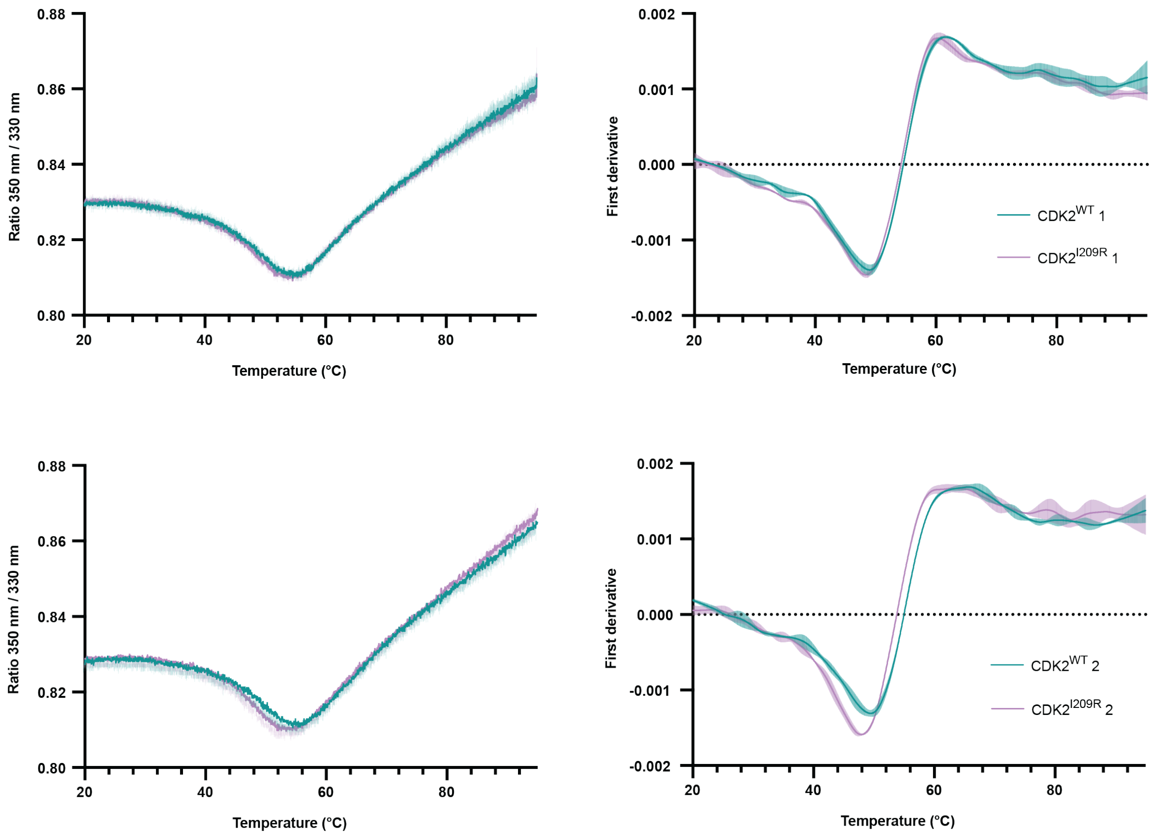

B

	T_m for ratio 350 nm / 330 nm (°C)	T_m at 330 nm (°C)	T_m at 350 nm (°C)
$CDK2^{WT}$ 1	48.8 ± 0.2	56.1 ± 0.1	56.6 ± 0.1
$CDK2^{I209R}$ 1	48.2 ± 0.0	55.5 ± 0.1	56.0 ± 0.1
ΔT_m	0.6	0.6	0.6

	T_m for ratio 350 nm / 330 nm (°C)	T_m at 330 nm (°C)	T_m at 350 nm (°C)
$CDK2^{WT}$ 2	49.2 ± 0.5	55.5 ± 0.1	55.8 ± 0.0
$CDK2^{I209R}$ 2	47.9 ± 0.3	53.4 ± 0.1	54.0 ± 0.1
ΔT_m	1.3	2.1	1.8

Fig. S8. NanoDSF analysis of CDK2 thermal stability. (**A**) Curves of the average fluorescence ratio signal (left) and first derivative (right) for two biological replicates (1 and 2) of $CDK2^{WT}$ (teal) and $CDK2^{I209R}$ (purple) (N = 3 technical replicates). Data were analysed by PR.ThermControl software (v2.3.1) and visualised using GraphPad Prism (v10.5.0). (**B**) Average melting temperatures ± standard deviation for each biological replicate, determined from the inflection point of thermograms of fluorescence at 330 nm, 350 nm and fluorescence ratio (350 nm/330 nm) (N = 3 technical replicates). Inflection points were assigned by PR.ThermControl software (v2.3.1).

```
CDK2          ............................................................
CDK1          ............................................................
CDK3          ............................................................
CDK4          ............................................................
CDK6          ............................................................
CDK9          ............................................................
CDK11     1   MSEDEERENENHLLVVPESRFDRDSGESEEAEEEVGEGTPQSSALTEGDYVPDSPALSPI

CDK2      1   ..................MENFQKVEKIGEGTYGVVYKARNKL.TGEVVALKKIRLDTE.
CDK1      1   ..................MEDYTKIEKIGEGTYGVVYKGRHKT.TGQVVAMKKIRLESE.
CDK3      1   ..................MDMFQKVEKIGEGTYGVVYKAKNRE.TGQLVALKKIRLDLE.
CDK4      1   ................MATSRYEPVAEIGVGAYGTVYKARDPH.SGHFVALKSVRVPNGG
CDK6      1   .........MEKDGLCRADQQYECVAEIGEGAYGKVFKARDLKNGGRFVALKRVRVQTG.
CDK9      1   .MA..KQYDSVECPFCDEVSKYEKLAKIGQGTFGEVFKARHRK.TGQKVALKKVLMENE.
CDK11    61   ELKQELPKYLPALQGCRSVEEFQCLNRIEEGTYGVVYRAKDKK.TDEIVALKRLKMEKE.

CDK2     41   ..TEGVPSTAIREISLLKE...LNHPNIVKLLDVIH........TENKLYLVFEFLHQDL
CDK1     41   ..EEGVPSTAIREISLLKE...LRHPNIVSLQDVLM........QDSRLYLIFEFLSMDL
CDK3     41   ..MEGVPSTAIREISLLKE...LKHPNIVRLLDVVH........NERKLYLVFEFLSQDL
CDK4     44   GGGGGLPISTVREVALLRRLEAFEHPNVVRLMDVCATSRT...DREIKVTLVFEHVDQDL
CDK6     51   ..EEGMPLSTIREVAVLRHLETFEHPNVVRLFDVCTVSRT...DRETKLTLVFEHVDQDL
CDK9     56   ..KEGFPITALREIKILQL...LKHENVVNLIEICRTKASPYNRCKGSIYLVFDFCEHDL
CDK11   119   ..KEGFPITSLREINTILK...AQHPNIVTVREIVVGS......NMDKIYIVMNYVEHDL

CDK2     88   KKFMDASA.LTGIPLPLIKSYLFQLLQGLAFCHSHRVLHRDLKPQNLLINTEGAIKLADF
CDK1     88   KKYLDSIPPGQYMDSSLVKSYLYQILQGIVFCHSRRVLHRDLKPQNLLIDDKGTIKLADF
CDK3     88   KKYMDSTP.GSELPLHLIKSYLFQLLQGVSFCHSHRVIHRDLKPQNLLINELGAIKLADF
CDK4    101   RTYLDKAP.PPGLPAETIKDLMRQFLRGLDFLHANCIVHRDLKPENILVTSGGTVKLADF
CDK6    106   TTYLDKVP.EPGVPTETIKDMMFQLLRGLDFLHSHRVVHRDLKPQNILVTSSGQIKLADF
CDK9    111   AGLLSNVLV..KFTLSEIKRVMQMLLNGLYYIHRNKILHRDMKAANVLITRDGVLKLADF
CDK11   168   KSLMETMKQ..PFLPGEVKTLMIQLLRGVKHLHDNWILHRDLKTSNLLLSHAGILKVGDF

                              T-loop
CDK2    147   GLARAFGVP....VRTYTHEVVTLWYRAPEILLGCKYYSTAVDIWSLGCIFAEMVTRRAL
CDK1    148   GLARAFGIP....IRVYTHEVVTLWYRSPEVLLGSARYSTPVDIWSIGTIFAELATKKPL
CDK3    147   GLARAFGVP....LRTYTHEVVTLWYRAPEILLGSKFYTTAVDIWSIGCIFAEMVTRKAL
CDK4    160   GLARIYSYQ....M.ALTPVVVTLWYRAPEVLLQS.TYATPVDMWSVGCIFAEMFRRKPL
CDK6    165   GLARIYSFQ....M.ALTSVVVTLWYRAPEVLLQS.SYATPVDLWSVGCIFAEMFRRKPL
CDK9    169   GLARAFSLAKNSQPNRYTNRVVTLWYRPPELLLGERDYGPPIDLWGAGCIMAEMWTRSPI
CDK11   226   GLAREYGSP....LKAYTPVVVTLWYRAPELLLGAKEYSTAVDMWSVGCIFGELLTQKPL

CDK2    203   FPGDSEIDQLFRIFRTLGTPDEVVWPGVTSMPDYKPS.FPKWARQDFSK.VVP.PLDEDG
CDK1    204   FHGDSEIDQLFRIFRALGTPNNEVWPEVESLQDYKNT.FPKWKPGSLAS.HVK.NLDENG
CDK3    203   FPGDSEIDQLFRIFRMLGTPSEDTWPGVTQLPDYKGS.FPKWTRKGLEE.IVP.NLEPEG
CDK4    214   FCGNSEADQLGKIFDLIGLPPEDDWPRDVSLPRG...AFPPRGPRPVQS.VVP.EMEESG
CDK6    219   FRGSSDVDQLGKILDVIGLPGEEDWPRDVALPRQ...AFHSKSAQPIEK.FVT.DIDELG
CDK9    229   MQGNTEQHQLALISQLCGSITPEVWPNVDNYELYEKLELVKGQKRKVKDRLKAYVRDPYA
CDK11   282   FPGKSEIDQINKVFKDLGTPSEKIWPGYSELPAVKKMTFSEHPYNNLRKRFGA.LLSDQG

CDK2    260   RSLLSQMLHYDPNKRISAKAALAHPFFQDVTKPVPHLRL.....................
CDK1    261   LDLLSKMLIYDPAKRISGKMALNHPYFNDLDNQIKKM.......................
CDK3    260   RDLLMQLLQYDPSQRITAKTALAHPYFSSPE.PSPAARQYVLQ......RFRH.......
CDK4    269   AQLLLEMLTFNPHKRISAFRALQHSYLHKDEGNPE.........................
CDK6    274   KDLLLKCLTFNPAKRISAYSALSHPYFQDLERCKENLDSHLPPSQ.NTSELNTA......
CDK9    289   LDLIDKLLVLDPAQRIDSDDALNHDFFWSDPMPSDLKGMLSTH...LTSMFEYLAPPRRK
CDK11   341   FDLMNKFLTYFPGRRISAEDGLKHEYFRETPLPIDPSMFPTWPAKSEQQRVKRGTSPRPP

CDK2          .......................................
CDK1          .......................................
CDK3          .......................................
CDK4          .......................................
CDK6          .......................................
CDK9    346   ..GSQITQQSTNQSR..........NPATTNQTEFERVF
CDK11   401   EGGLGYSQLGDDDLKETGFHLTTTNQGASAAGPGFSLKF
```

Fig. S9. Multiple sequence alignment of CAK substrate CDKs. Multiple sequence alignment of CDKs that are known substrates of the CAK: CDK2, CDK1, CDK3, CDK4, CDK6, CDK9, and CDK11 (CDK11B^{p58}). Residues of CDK2 that interact with CDK7 (based on the AMP-PNP-CAK-CDK2-cyclin A2 structure) are indicated by salmon-coloured triangles.

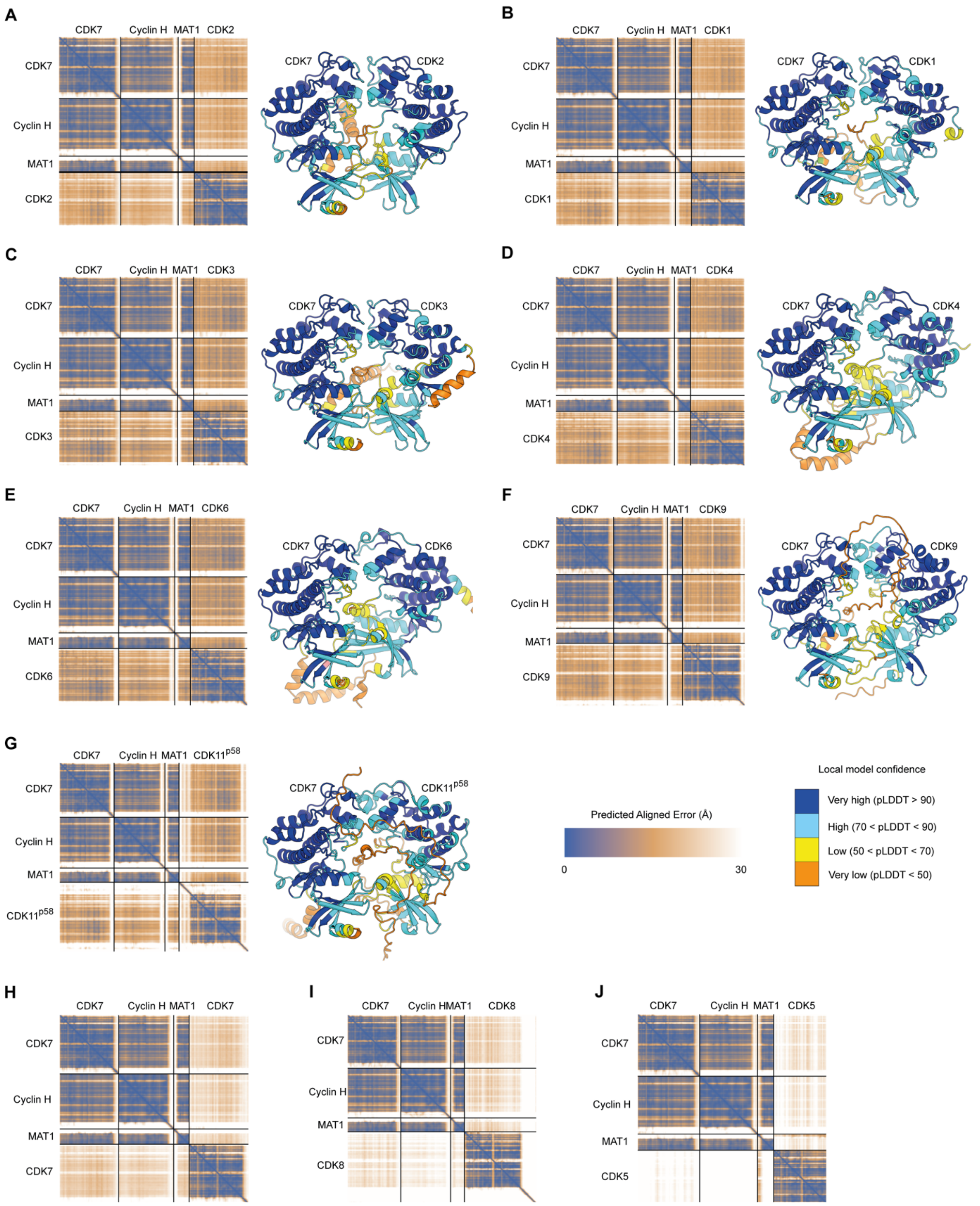

Fig. S10. PAE and pLDDT plots for AlphaFold3-predicted CAK-CDK complexes. (**A-G**) PAE and pLDDT plots for the AlphaFold3-predicted complexes between CAKΔN (the CAK with N-terminal 219 residues of MAT1 excluded) and substrate CDKs shown in Fig. 3. (**H-J**) PAE plots for AlphaFold3-predicted complexes between CAKΔN and non-substrate CDKs.

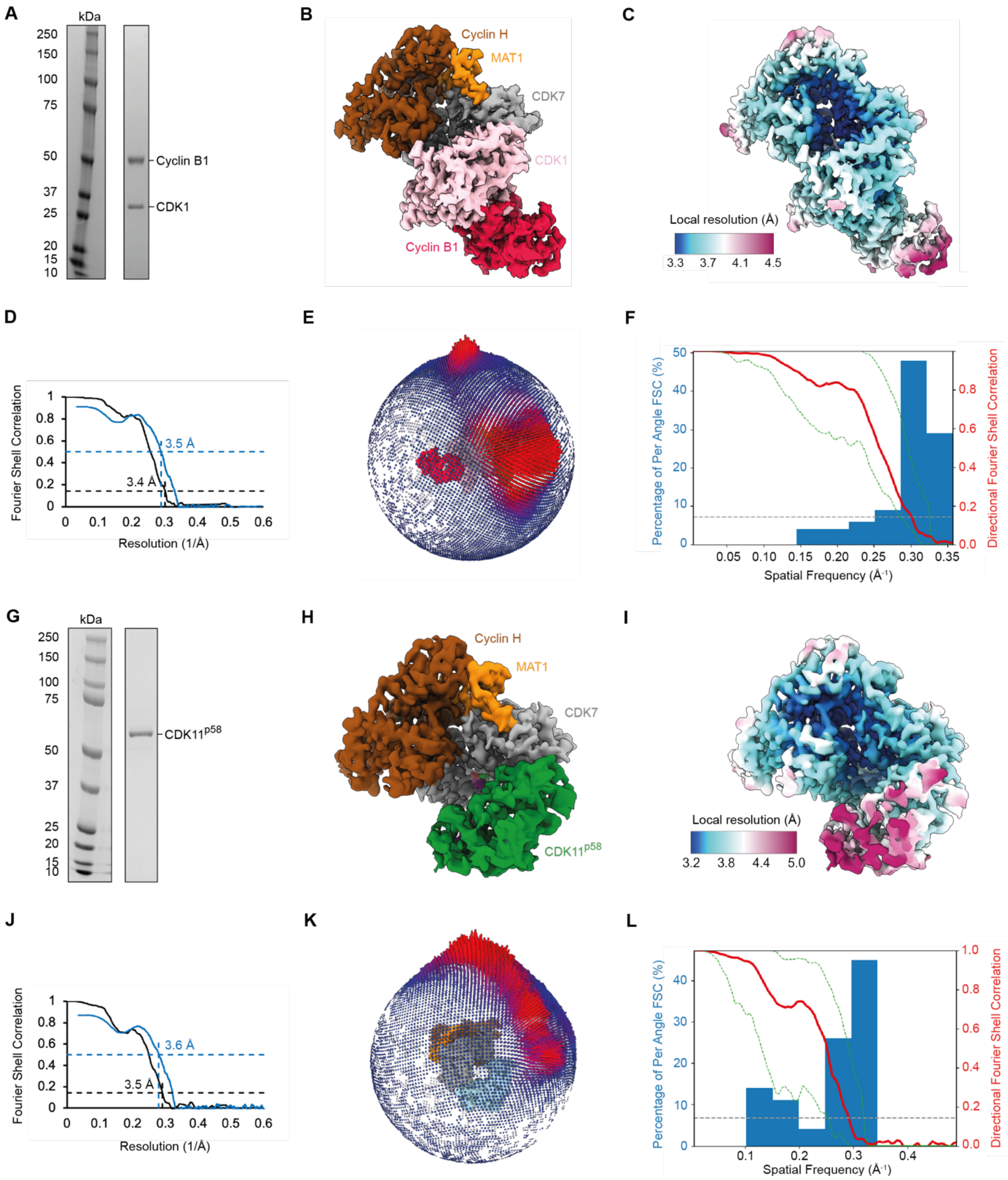

Fig. S11. Validation of the CAK-CDK1-cyclin B1 and CAK-CDK11 complex structures. (**A**) SDS-PAGE of the purified CDK1-cyclin B1 complex. (**B**) Cryo-EM reconstruction of the CAK-CDK1-cyclin B1 complex. (**C**) Cryo-EM reconstruction shown in panel B coloured by local resolution. (**D**) FSC curves for the CAK-CDK1-cyclin B1 cryo-EM reconstruction and the refined atomic model. The half-map FSC curve is shown in black and the model vs. map FSC curve is shown in blue. Resolutions are estimated according to the FSC = 0.143 criterion for half-maps, and the FSC = 0.5 criterion for model vs. map FSCs. (**E**) Orientation distribution plot for the CAK-CDK1-cyclin B1 structure. (**F**) Assessment of the CAK-CDK1-cyclin B1 cryo-EM reconstruction by 3D FSC. The sphericity of the reconstruction is 0.86. (**G**) SDS-PAGE of the purified CDK11^{p58} protein. (**H**) Cryo-EM reconstruction of the CAK-CDK11 complex. (**I**) Cryo-EM reconstruction shown in panel H coloured by local resolution. (**J**) FSC

curves for the CAK-CDK11 cryo-EM reconstruction and the refined atomic model. The half-map FSC curve is shown in black and the model vs. map FSC curve is shown in blue. Resolutions are estimated according to the FSC = 0.143 criterion for half-maps, and the FSC = 0.5 criterion for model vs. map FSCs. (**K**) Orientation distribution plot for the CAK-CDK11 structure. (**L**) Assessment of the CAK-CDK11 cryo-EM reconstruction by 3D FSC. The sphericity of the reconstruction is 0.85.

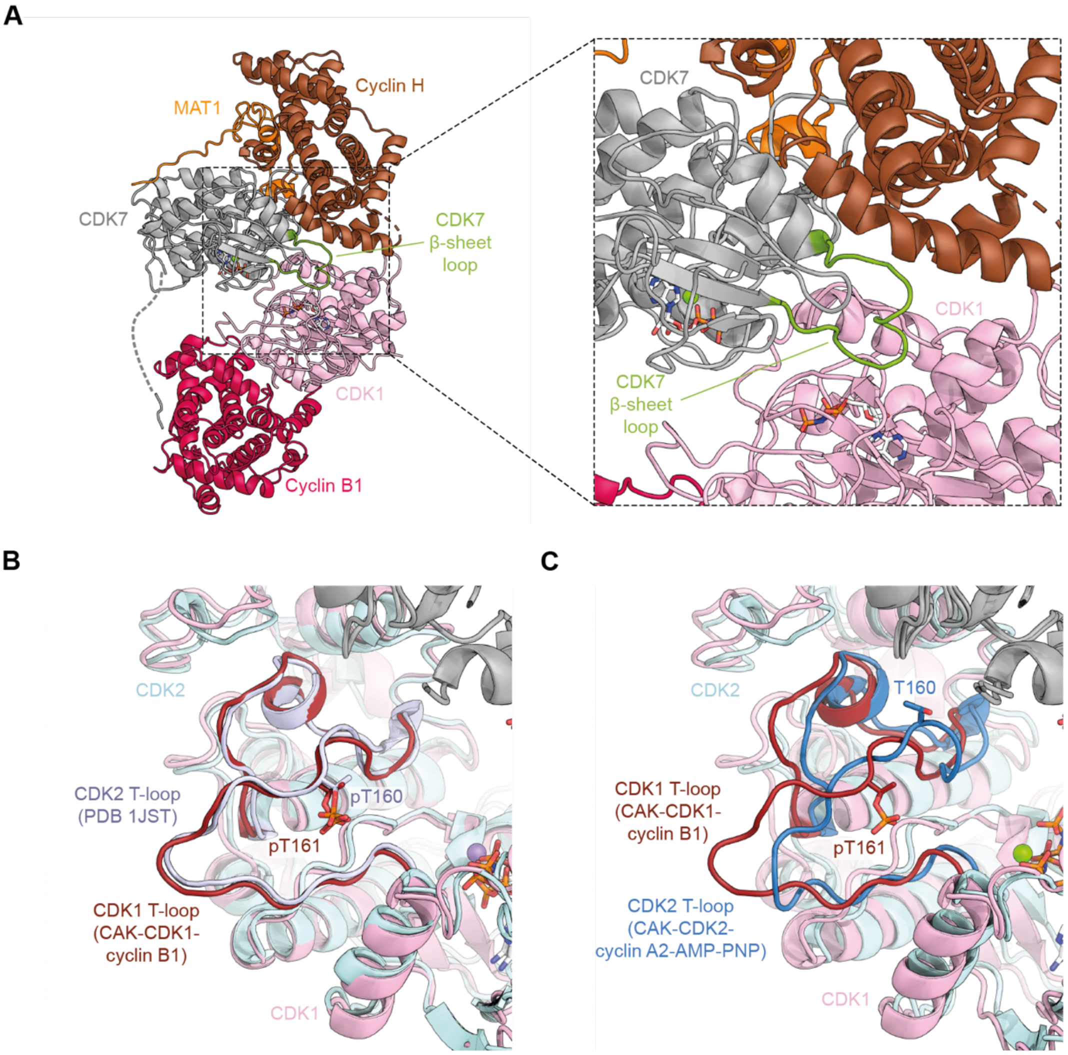

Fig. S12. Features of the CAK-CDK1-cyclin B1 complex. (**A**) Side view of the CAK-CDK1-cyclin B1 complex. Inset: Close-up view showing the CDK7 β-sheet loop inserting into the CDK1 N-terminal lobe. This interaction is essentially identical to that seen in the CAK-CDK2(-cyclin A2) complexes. (**B**, **C**) Superpositions of the CAK-CDK1-cyclin B1 complex with the structure of a phosphorylated CDK2-cyclin A complex (PDB 1JST (*19*)) (B) and with the CAK-CDK2-cyclin A2-AMP-PNP complex (C), highlighting the T-loop conformations in each case. For clarity, the bound cyclin is omitted in each panel.

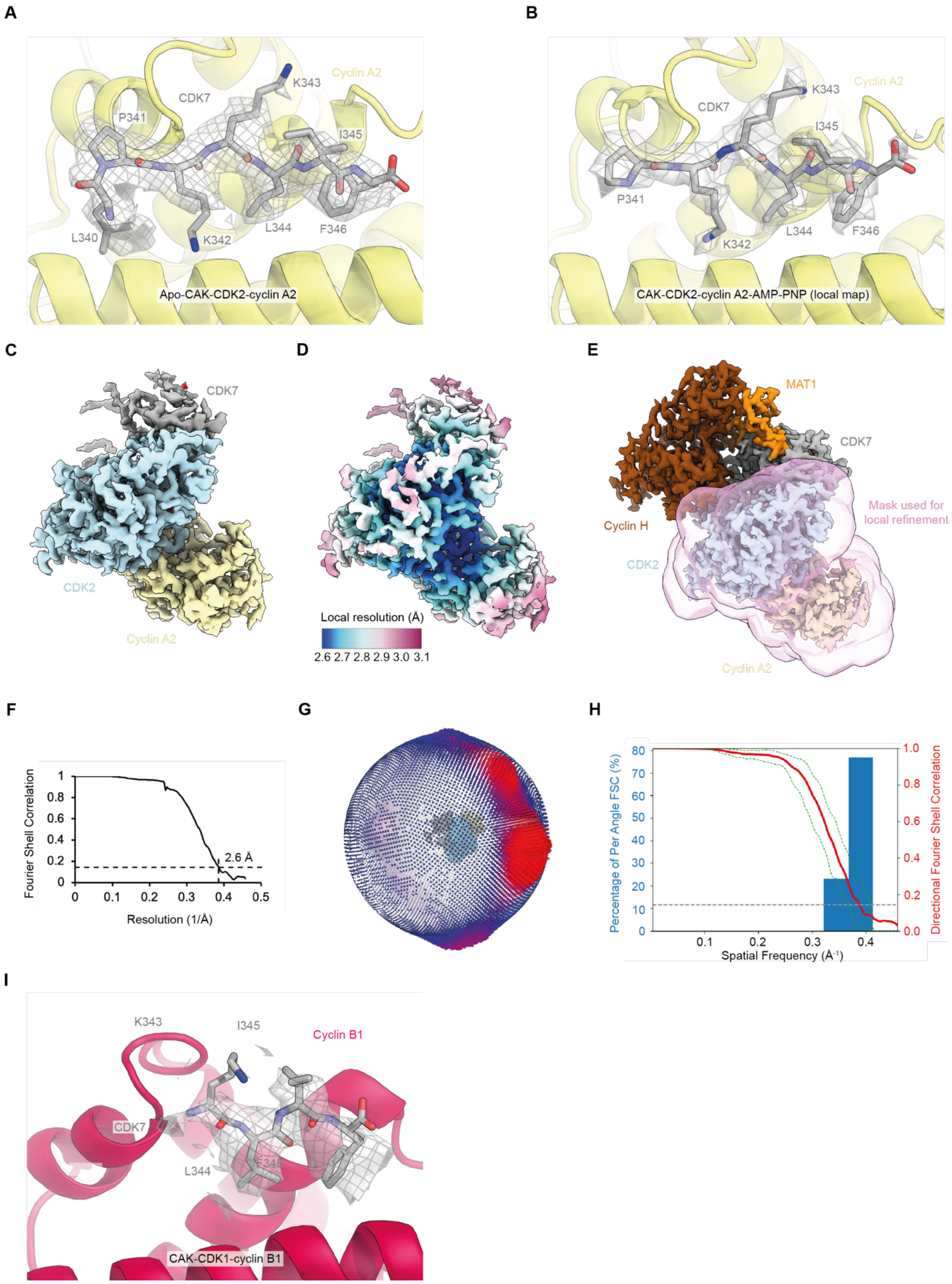

Fig. S13. Further analysis of the interaction between the CDK7 RxL motif and cyclins. (**A**) Close-up view of CDK7 residues Leu340-Phe346 interacting with cyclin A2 in the apo-CAK-CDK2-cyclin A2 complex, with the cryo-EM density shown as a mesh. Map contour level = 3.5 σ; carving = 2.0 Å. (**B**) Close-up view of CDK7 residues Pro341-Phe346 interacting with cyclin A2 in the CAK-CDK2-cyclin A2-AMP-PNP complex, with the cryo-EM density

resulting from the locally refined reconstruction shown as a mesh. Map contour level = 3.5 σ; carving = 2.0 Å. (**C**) Locally refined cryo-EM reconstruction of the CAK-CDK2-cyclin A2-AMP-PNP complex. (**D**) Cryo-EM reconstruction shown in panel C coloured by local resolution. (**E**) Cryo-EM reconstruction of the CAK-CDK2-cyclin A2-AMP-PNP complex superposed with the mask used for local refinement. (**F**) FSC curve for the locally refined CAK-CDK2-cyclin A2-AMP-PNP reconstruction. The resolution is estimated according to the FSC = 0.143 criterion for half-maps. (**G**) Orientation distribution plot for the locally refined CAK-CDK2-cyclin A2-AMP-PNP cryo-EM reconstruction. (**H**) Assessment of the locally refined CAK-CDK2-cyclin A2-AMP-PNP cryo-EM reconstruction by 3D FSC. The sphericity of the reconstruction is 0.93. (I) Close-up view of CDK7 residues Lys343-Phe346 interacting with cyclin B1 in the CAK-CDK1-cyclin B1 complex, with the cryo-EM density shown as a mesh. Map contour level = 3.5 σ; carving = 2.5 Å.

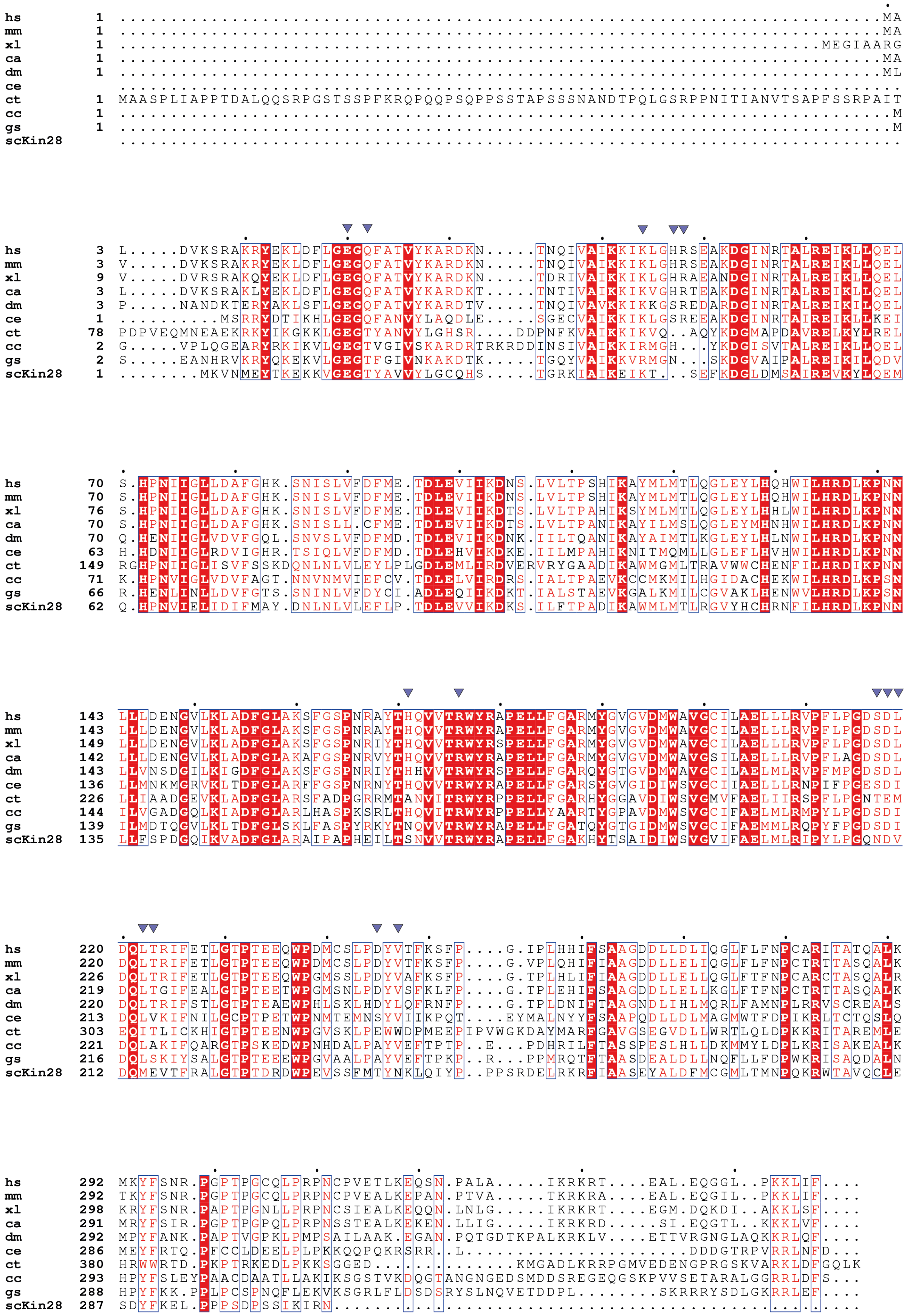

Fig. S14. Multiple sequence alignment of CDK7 homologues from different organisms. Multiple sequence alignment of CDK7 homologues from different organisms. UniProt

identifiers are provided with the protein names. Organisms: *Homo sapiens, hs* (CDK7; P50613) *Mus musculus, mm* (CDK7; Q03147); *Xenopus laevis, xl* (CDK7;); *Carassius auratus, ca* (CDK7; P51953); *Drosophila melanogaster, dm* (CDK7; Q24216); *Caenorhabditis elegans, ce* (CDK7; G5EFV5); *Chaetomium thermophilum, ct* (protein kinase domain-containing protein; G0SFC6); *Chondrus crispus, cc* (RNA polymerase subunit kinase; R7QKX5); *Galdieria sulphuraria, gs* (RNA polymerase subunit kinase; M2XXR7); and *Saccharomyces cerevisiae, scKin28* (serine/threonine-protein kinase Kin28; P06242). Residues of human CDK7 that interact with CDK2 (based on the CAK-CDK2-cyclin A2- AMP-PNP structure) are indicated by purple triangles.

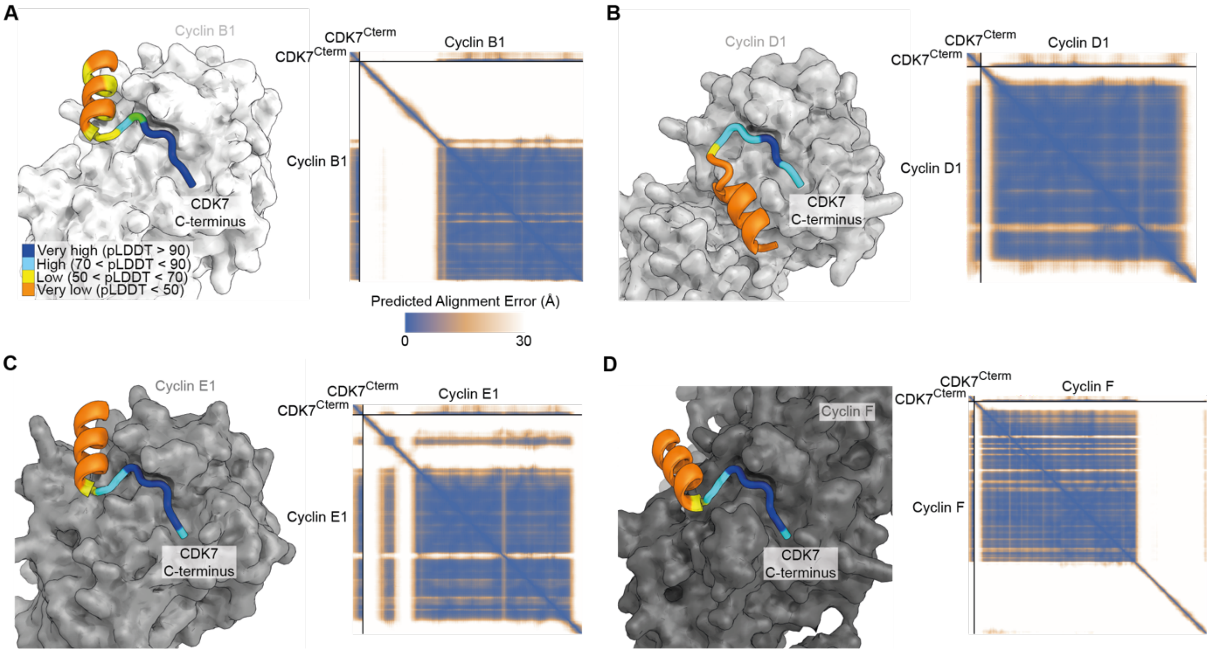

Fig. S15. AlphaFold3-predicted interactions between the CDK7 RxL motif and cyclins. (**A**) Left: AlphaFold3 structure prediction of cyclin B1 in complex with a peptide encompassing the C-terminal 20 residues of CDK7 (CDK7^{Cterm}). CDK^{Cterm} is coloured by the pLDDT score in each panel. Right: PAE plots for the AlphaFold3-predicted complexes. (**B**, **C**) As in A, but for cyclin D1 (B) and cyclin E1 (C). (**D**) As in A, but for cyclin F. Cyclin F does not bind a CDK partner but is instead a component of the SCF E3 ubiquitin ligase complex (*67*) and has been identified as a binder of the CDK7 RxL motif in a recent high-throughput RxL motif identification study (*68*).

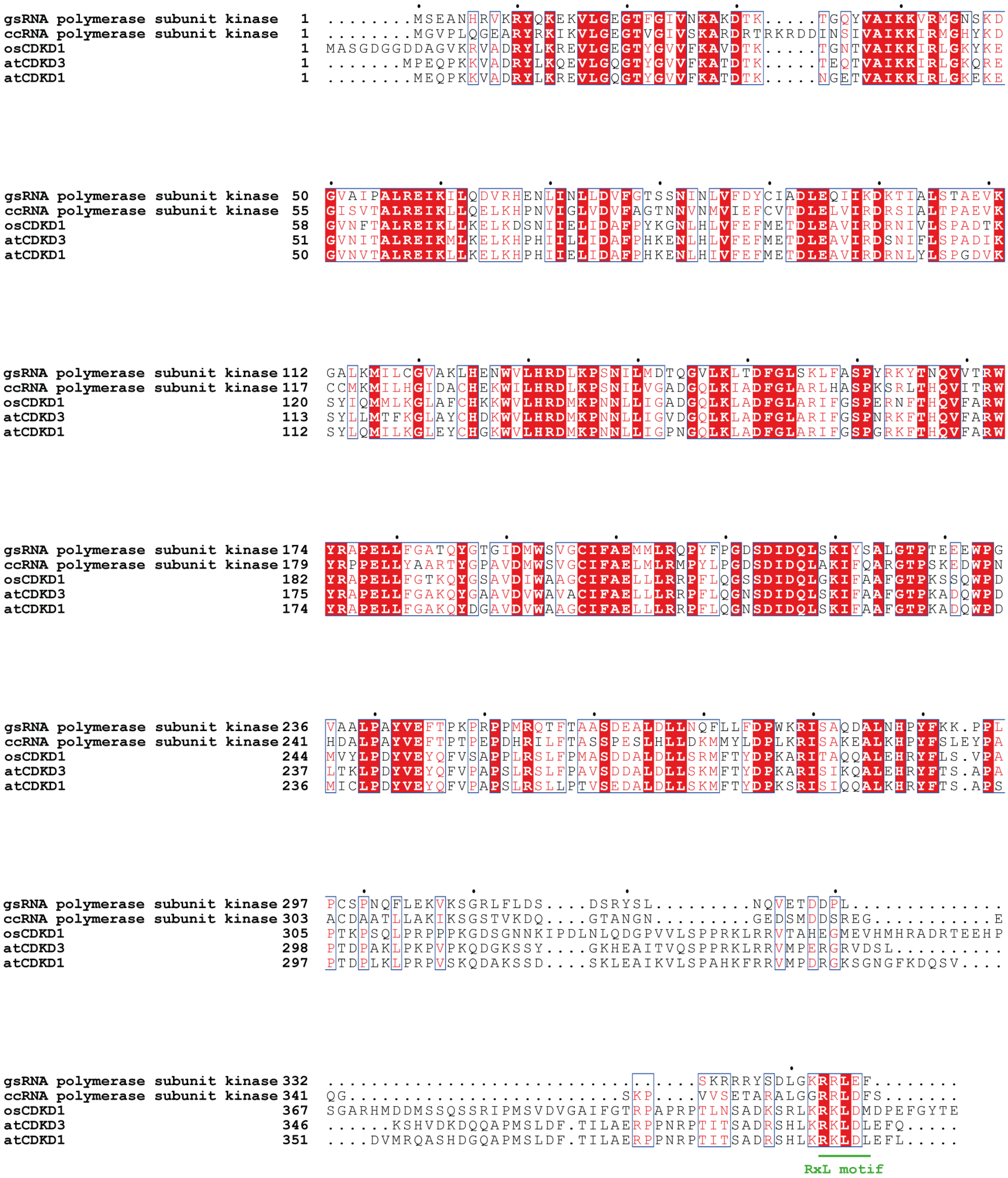

Fig. S16. Multiple sequence alignment of CDK7 homologues from plants. The RxL motif near the C-terminus of different plant CDK7 homologues is indicated. UniProt identifiers are provided with the protein names. Organisms: *Galdieria sulphuraria* (gsRNA polymerase subunit kinase; M2XXR7); *Chondrus crispus* (ccRNA polymerase subunit kinase; R7QKX5); *Oryza sativa japonica* (osCDKD1; P29620); *Arabidopsis thaliana* (atCDKD1, atCDKD3; Q9C9U2, Q9LMT0).

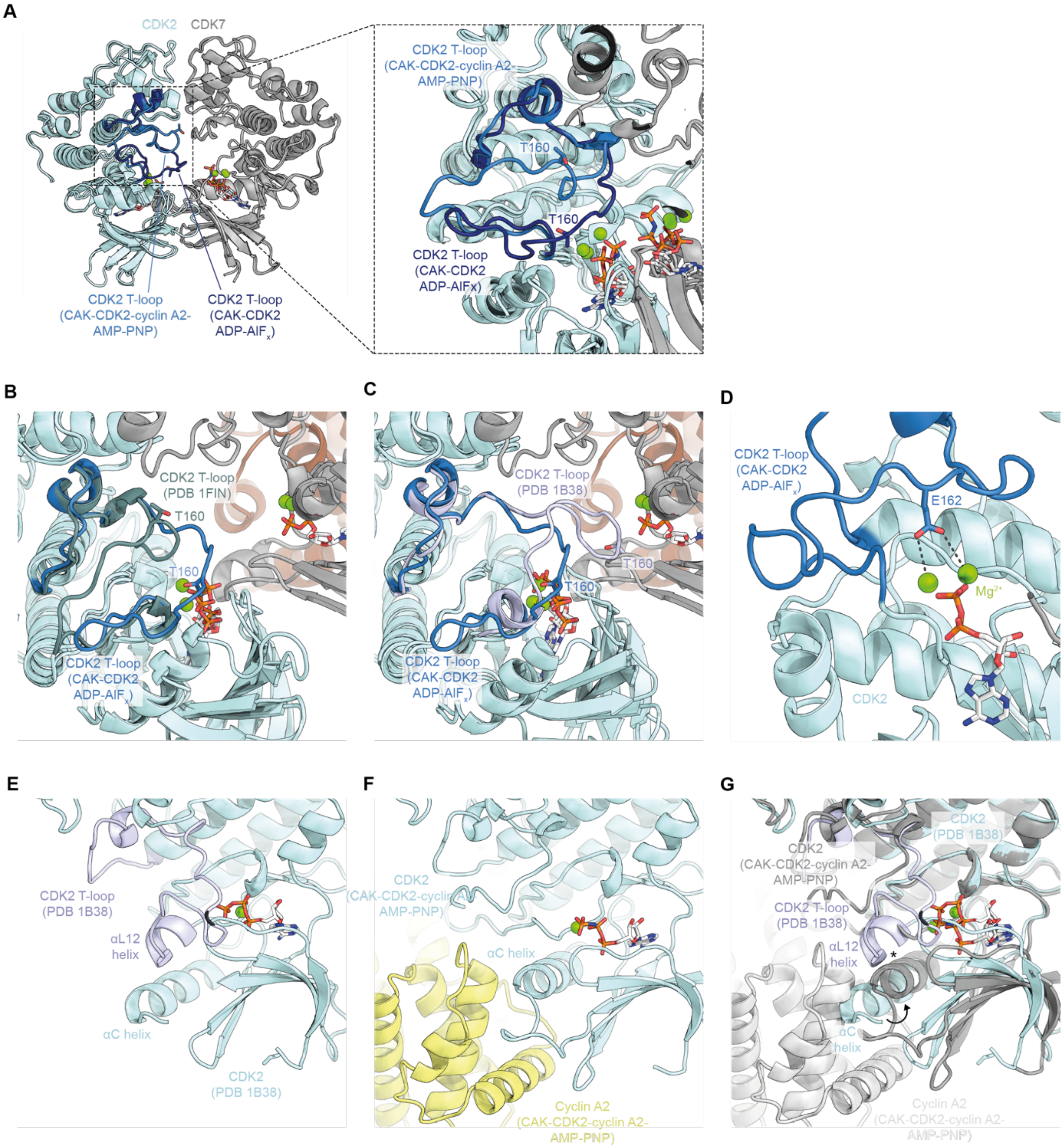

Fig. S17. Further analysis of T-loop conformations in CAK-CDK2 and CAK-CDK2-cyclin A2 complexes. (**A**) Superposition of the kinase-kinase interfaces in the CAK-CDK2-cyclin A2-AMP-PNP and CAK-CDK2 (ADP-AlF$_x$) complexes. Inset: Close-up view showing the different conformations of the CDK2 T-loop in each complex. (**B**) Superposition of the atomic model of the CAK-CDK2 (ADP-AlF$_x$) complex with a crystal structure of CDK2-cyclin A (PDB 1FIN (*18*); unphosphorylated T-loop; cyclin not shown for clarity). (**C**) Superposition of the atomic model of the CAK-CDK2 (ADP-AlF$_x$) complex with a crystal structure of monomeric CDK2 (PDB 1B38 (*43*)), in the same view as panel C. The T-loop conformation observed in free, monomeric CDK2 would bring Thr160 into close proximity of the CDK7 active site. (**D**) Close-up view of the CDK2 T-loop in the CAK-CDK2 (ADP-AlF$_x$) complex showing salt bridges formed between Glu162 and the Mg^{2+} ions in the CDK2 active site, which trap the flexible T-loop in an intermediate conformation. (**E**) Another view of the T-loop in the crystal structure of monomeric, inactive CDK2 (PDB 1B38 (*43*)). (**F**) Close-up view of the

atomic model of the CAK-CDK2-cyclin A2-AMP-PNP complex in the same view as in panel F. Only CDK2 and cyclin A2 are shown, for clarity. (**G**) Superposition of the atomic model of the CAK-CDK2-cyclin A2-AMP-PNP complex with the crystal structure of monomeric, inactive CDK2 (PDB 1B38 (*43*)) in the same view as in panels F and G. Monomeric CDK2 is shown in colour, while the CDK2 and cyclin A2 subunits of the AMP-PNP-CAK-CDK2-cyclin A2 complex are shown in shades of grey. The conformation of the T-loop of free, inactive CDK2 is sterically incompatible with the presence of bound cyclin A2; cyclin binding causes the αC helix of CDK2 to move inwards (represented by a black arrow), 'melting' the CDK2 αL12 helix (represented by an asterisk) and leading to a structural rearrangement of the T-loop (*18*).

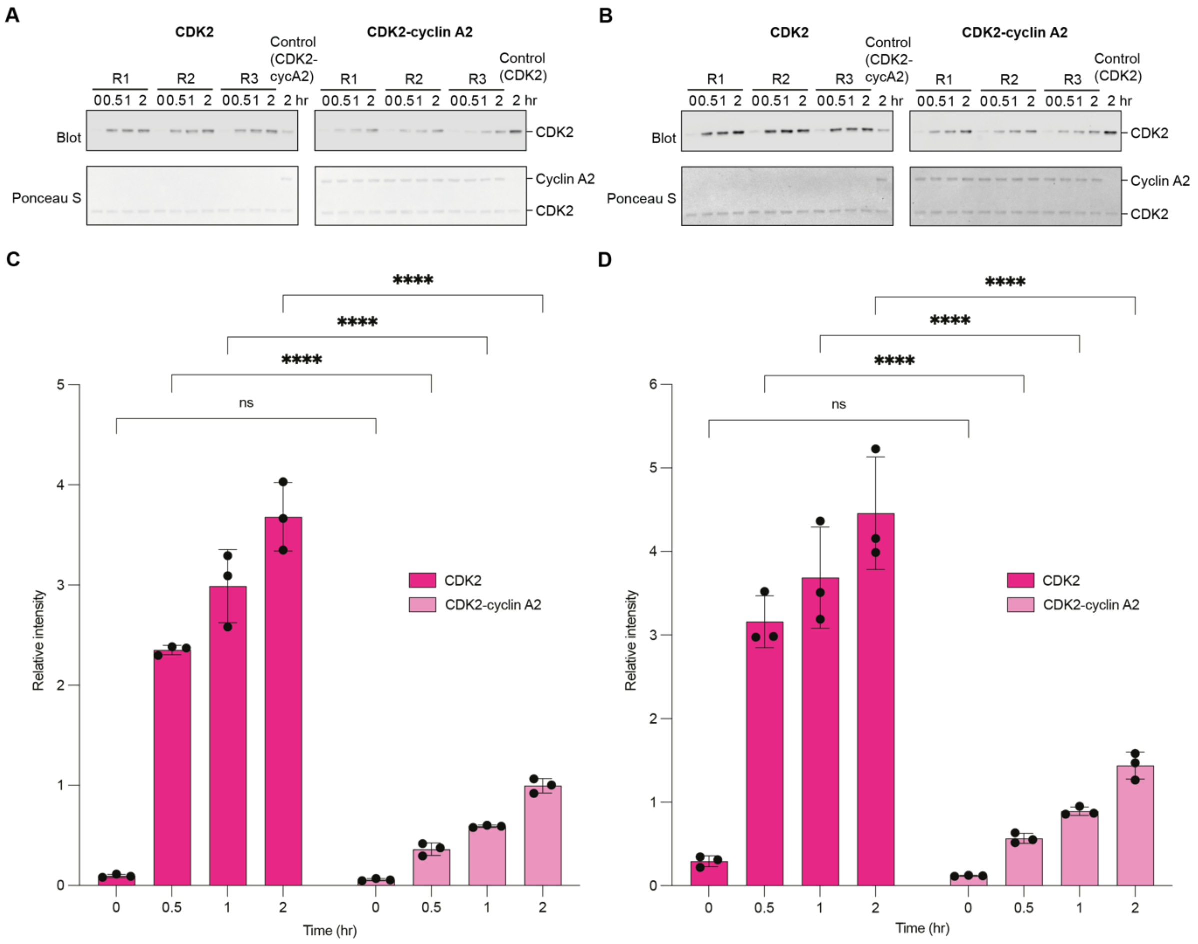

Fig. S18. CDK2 is less readily phosphorylated by the CAK in the presence of bound cyclin. (**A**, **B**) Western blots against phosphorylated CDK2 and Ponceau S-stained loading controls for assays assessing the activity of the CAK towards CDK2^{D145N} (left) and CDK2^{D145N}-cyclin A2 (right). The catalytically inactive CDK2^{D145N} mutation was used to prevent possible interference from the formation of catalytically fully active CDK2-cyclin A2 complexes during the reaction. Two biologically independent sets of experiments were performed (A, B), each with N = 3 technical replicates (R1, R2, and R3). (**C**) Quantification of Western blot band intensities for the data shown in panel A, presented as the mean ± standard deviation of N = 3 technical replicates. *P*-values obtained by two-way ANOVA and Šídák's multiple comparisons test: CDK2 0 hr vs. CDK2-cyclin A2 0 hr $P = 0.9983$; CDK2 0.5 hr vs. CDK2-cyclin A2 0.5 hr $P < 0.0001$; CDK2 1 hr vs. CDK2-cyclin A2 1 hr $P < 0.0001$; CDK2 2 hr vs. CDK2-cyclin A2 2 hr $P < 0.0001$. (ns = $P > 0.05$; **** = $P < 0.0001$). (**D**) Quantification of Western blot band intensities for the data shown in panel B, presented as the mean ± standard deviation of N = 3 technical replicates. *P*-values obtained by two-way ANOVA and Šídák's multiple comparisons test: CDK2 0 hr vs. CDK2-cyclin A2 0 hr $P = 0.9581$; CDK2 0.5 hr vs. CDK2-cyclin A2 0.5 hr $P < 0.0001$; CDK2 1 hr vs. CDK2-cyclin A2 1 hr $P < 0.0001$; CDK2 2 hr vs. CDK2-cyclin A2 2 hr $P < 0.0001$. (ns = $P > 0.05$; **** = $P < 0.0001$).

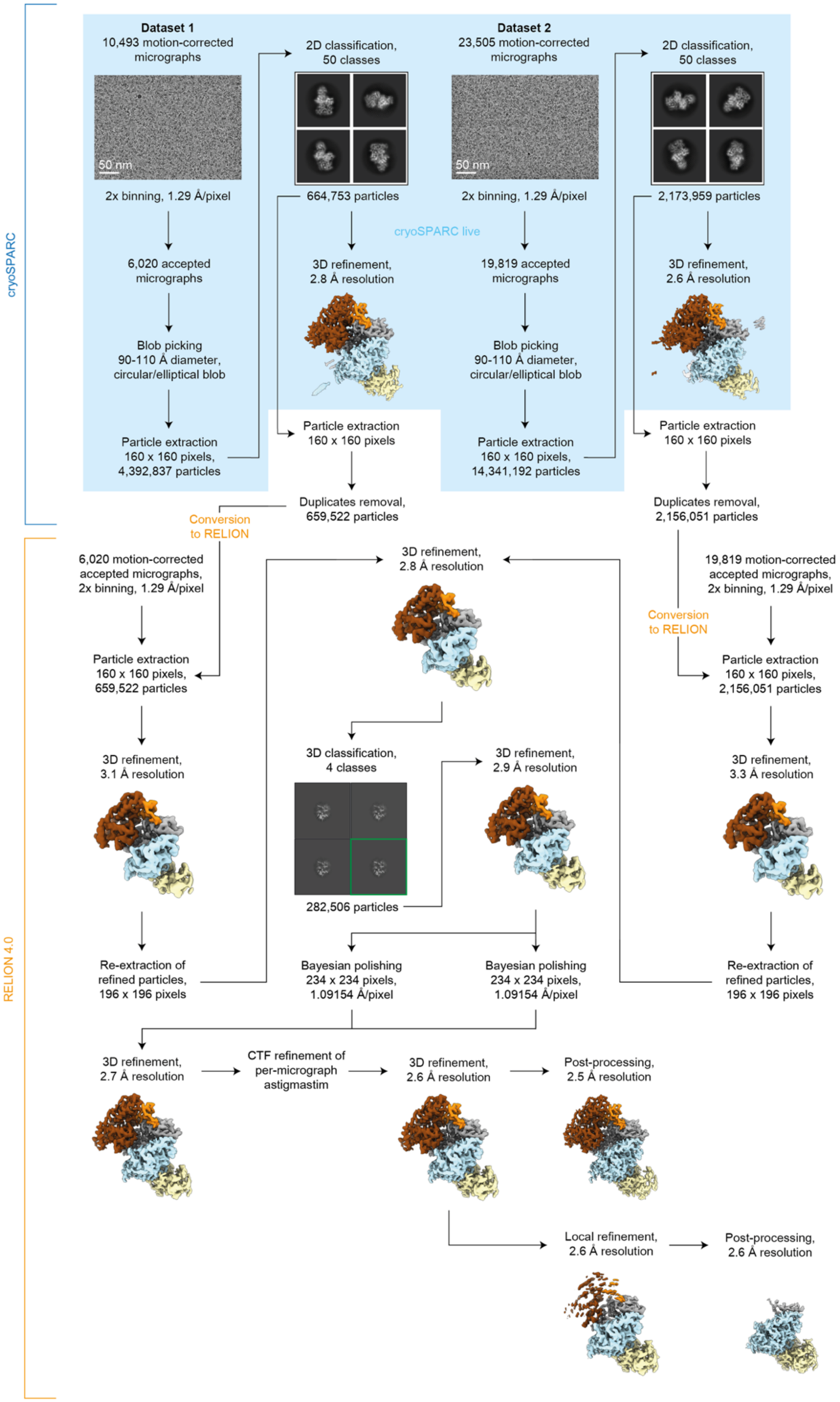

Fig. S19. Data processing workflow for the CAK-CDK2-cyclin A2-AMP-PNP complex. See Methods for details.

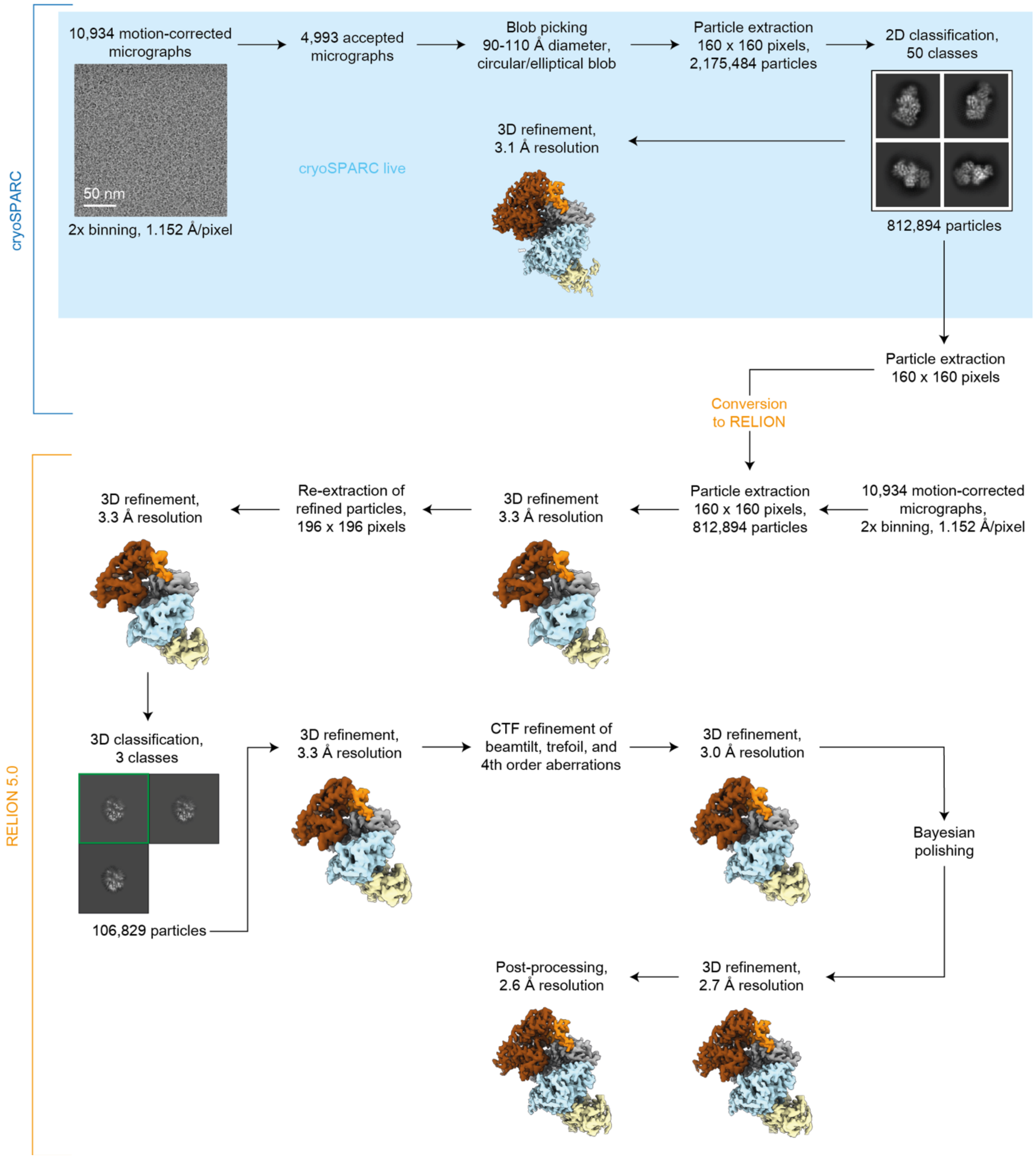

Fig. S20. Data processing workflow for the apo-CAK-CDK2-cyclin A2 complex. See Methods for details.

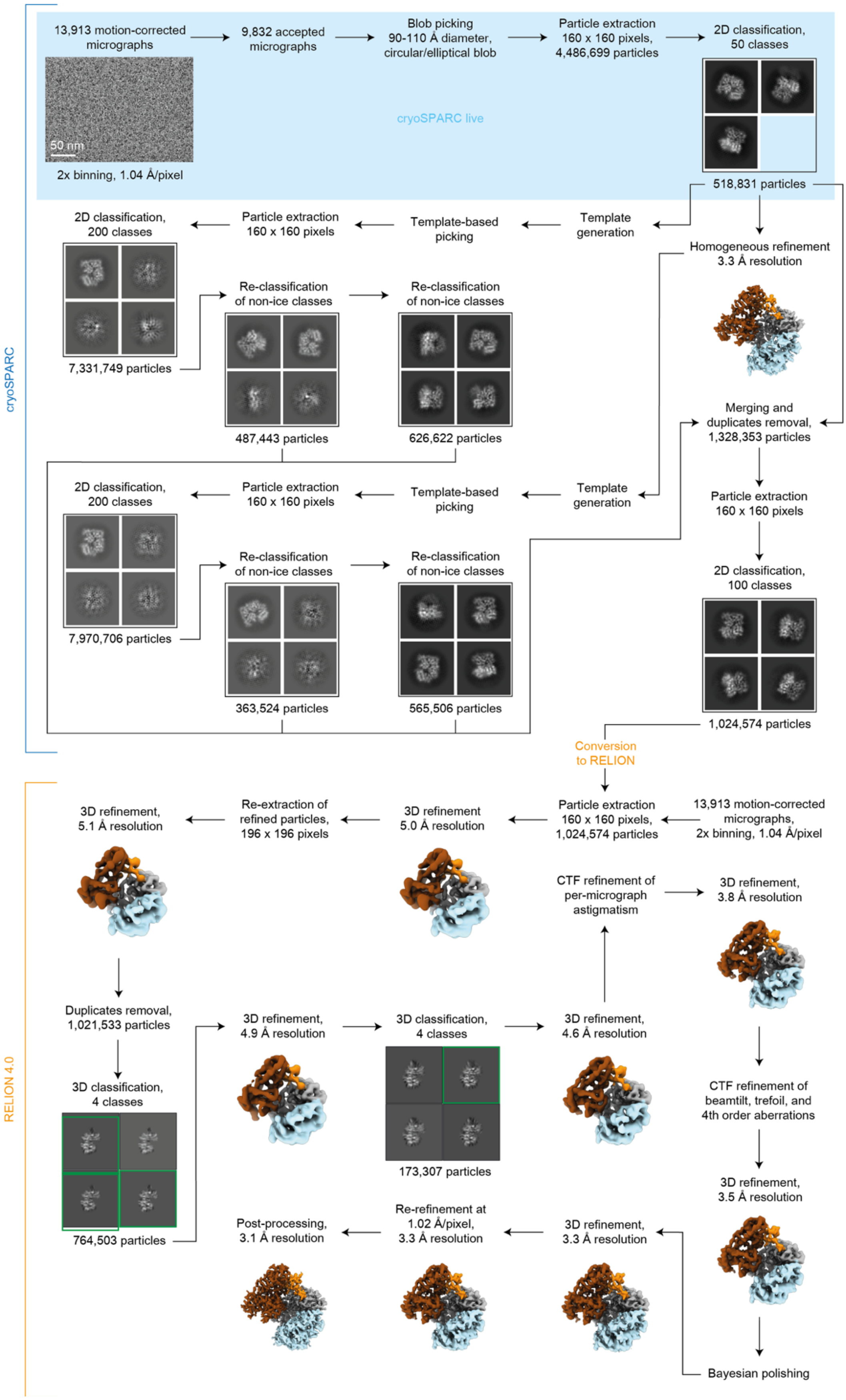

Fig. S21. Data processing workflow for the CAK-CDK2 ADP-nitrate complex. See Methods for details.

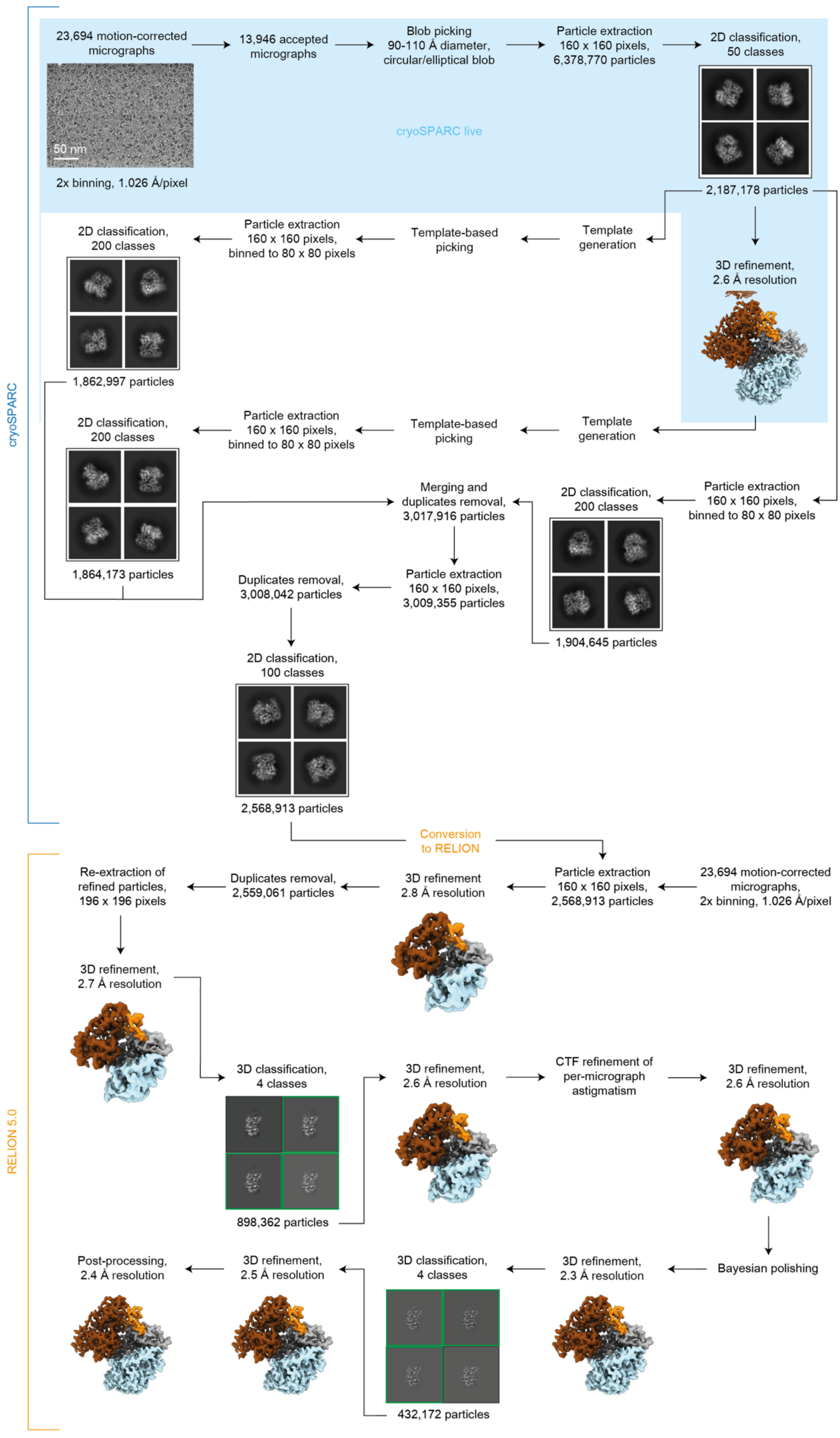

Fig. S22. Data processing workflow for the CAK-CDK2 ADP-AlF$_x$ complex. See Methods for details.

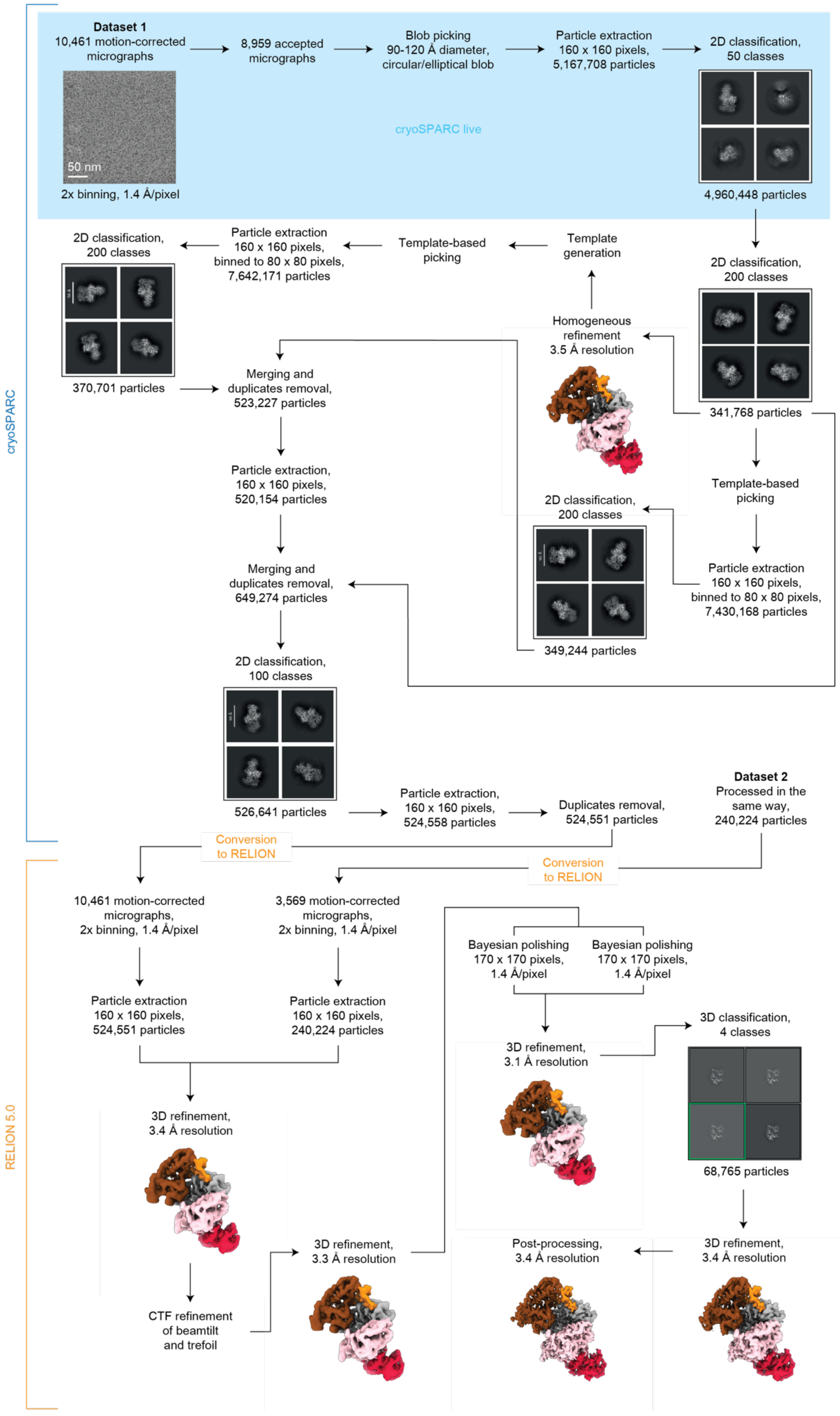

Fig. S23. Data processing workflow for the CAK-CDK1-cyclin B1 complex. See Methods for details.

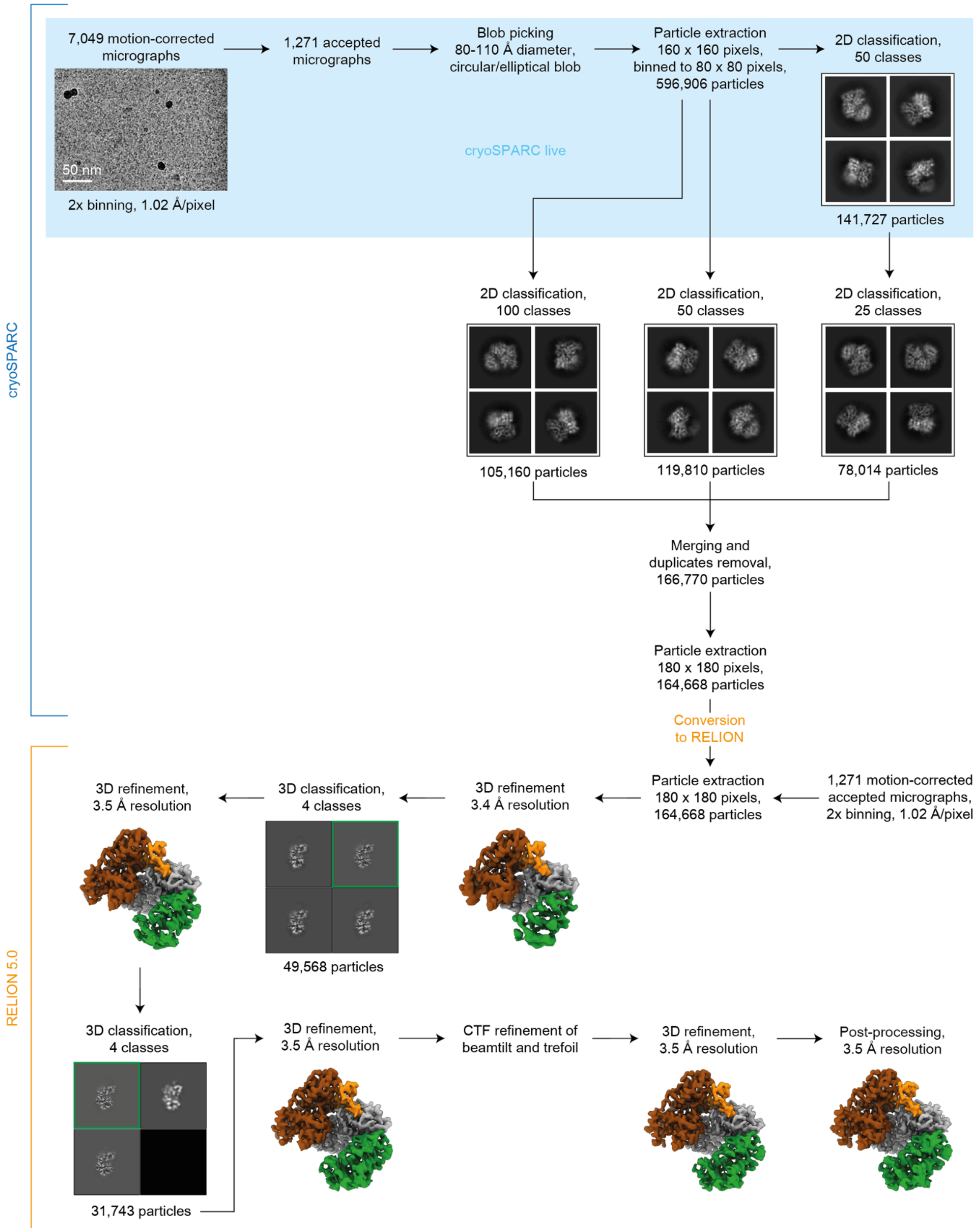

Fig. S24. Data processing workflow for the CAK-CDK11 complex. See Methods for details.

Table S1. Cryo-EM data collection, 3D reconstruction, refinement, and validation statistics (part 1)

**Complex**	**CAK-CDK2-cyclin A2-AMP-PNP, full map**	**CAK-CDK2-cyclin A2-AMP-PNP, local map**	**Apo-CAK-CDK2-cyclin A2**
Microscope	Krios		Krios
Stage type	Autoloader		Autoloader
Voltage (kV)	300		300
Detector	K3		Falcon 4i
Energy filter	BioQuantum		Selectris X
Acquisition mode	Counting		Counting
Physical pixel size (Å)	0.645		0.576
Defocus range (μm)	0.4-2.2		0.6-2.0
Electron exposure (e^-/A^2)	50		70
**Reconstruction**	**EMD-53027**	**EMD-52759**	**EMD-53028**
Software	RELION 4.0	RELION 4.0	RELION 4.0
Particles used	282,506	282,506	106,829
Box size (pixels)	234 x 234 x 234	234 x 234 x 234	196 x 196 x 196
Final pixel size (Å)	1.09154	1.09154	1.152
Accuracy rotations (°)	1.016	1.281	0.962
Accuracy translations (Å)	0.332	0.439	0.346
Map resolution (Å)	2.5	2.6	2.6
Map resolution range (Å)	2.4-3.3	2.6-3.1	2.6-3.4
Sphericity	0.942	0.930	0.967
Map sharpening B-factor ($Å^2$)	-55	-56	-10
**Coordinate refinement**			
Software	PHENIX	N/A	PHENIX
Refinement algorithm	REAL SPACE	N/A	REAL SPACE
Resolution cutoff (Å)	2.5	N/A	2.6
$FSC_{model\text{-}vs\text{-}map}$=0.5 (Å)	2.6	N/A	2.7
**Model**	**PDB-9QCV**	N/A	**PDB-9QCX**
Number of residues	1251	N/A	1196
Protein	1197	N/A	1195
Ligand	5	N/A	1
Water	49	N/A	0
B-factors overall	47.16	N/A	81.36
Protein	47.25	N/A	81.37
Ligand	51.20	N/A	62.82
Water	24.57	N/A	N/A
R.M.S. deviations			
Bond lengths (Å)	0.003	N/A	0.005
Bond angles (°)	0.565	N/A	0.978
**Validation**			
Molprobity score	1.43	N/A	1.60
Molprobity clashscore	7.09	N/A	5.68
Rotamer outliers (%)	1.13	N/A	1.80
C_β deviations (%)	0.00	N/A	0.00
Ramachandran plot			
Favored (%)	98.56	N/A	97.54
Allowed (%)	1.44	N/A	2.46
Outliers (%)	0.00	N/A	0.00

Table S2. Cryo-EM data collection, 3D reconstruction, refinement, and validation statistics (part 2)

**Complex**	**CAK-CDK2 (ADP-nitrate)**	**CAK-CDK2 (ADP-AlF$_x$)**
Microscope	Krios	Krios
Stage type	Autoloader	Autoloader
Voltage (kV)	300	300
Detector	K3 BioQuantum	K3
Acquisition mode	Counting	Counting
Physical pixel size (Å)	0.51	0.513
Defocus range (μm)	0.5-2.0	0.5-2.1
Electron exposure (e^-/A^2)	60	70
**Reconstruction**	**EMD-52760**	**EMD-52761**
Software	RELION 4.0	RELION 4.0
Particles used	173,307	432,172
Box size (pixels)	196 x 196 x 196	196 x 196 x 196
Final pixel size (Å)	1.02	1.026
Accuracy rotations (°)	1.957	1.155
Accuracy translations (Å)	0.605	0.343
Map resolution (Å)	3.1	2.4
Map resolution range (Å)	3.1-3.7	2.4-3.0
Sphericity	0.858	0.876
Map sharpening B-factor (Å^2)	-98	-20
**Coordinate refinement**		
Software	PHENIX	PHENIX
Refinement algorithm	REAL SPACE	REAL SPACE
Resolution cutoff (Å)	3.1	2.4
$FSC_{model\text{-}vs\text{-}map}$=0.5 (Å)	3.4	2.5
**Model**	**PDB-9I9J**	**PDB-9I9K**
Number of residues	876	946
Protein	870	919
Ligand	4	7
Water	2	20
B-factors overall	47.56	61.05
Protein	47.40	61.10
Ligand	68.97	62.12
Water	16.39	41.63
R.M.S. deviations		
Bond lengths (Å)	0.002	0.005
Bond angles (°)	0.526	1.023
**Validation**		
Molprobity score	1.59	1.45
Molprobity clashscore	6.65	5.68
Rotamer outliers (%)	1.57	1.24
C_β deviations (%)	0.00	0.00
Ramachandran plot		
Favored (%)	97.64	97.67
Allowed (%)	2.36	2.33
Outliers (%)	0.00	0.00

Table S3. Cryo-EM data collection, 3D reconstruction, refinement, and validation statistics (part 3)

Complex	CAK-CDK1-cyclin B1	CAK-CDK11
Microscope	Glacios	Krios
Stage type	Autoloader	Autoloader
Voltage (kV)	200	300
Detector	Falcon 4i	K3 BioQuantum
Acquisition mode	Counting	Counting
Physical pixel size (Å)	0.7	0.52
Defocus range (µm)	0.5-1.9	0.7-2.0
Electron exposure (e^-/A^2)	60	70
**Reconstruction**	**EMD-54971**	**EMD-52758**
Software	RELION 5.0	RELION 5.0
Particles used	68,765	31,743
Box size (pixels)	170 x 170 x 170	180 x 180 x 180
Final pixel size (Å)	1.4	1.02
Accuracy rotations (°)	1.311	1.964
Accuracy translations (Å)	0.428	0.556
Map resolution (Å)	3.4	3.5
Map resolution range (Å)	3.3-4.5	3.2-5.0
Sphericity	0.864	0.851
Map sharpening B-factor ($Å^2$)	-40	-10
**Coordinate refinement**		
Software	PHENIX	PHENIX
Refinement algorithm	REAL SPACE	REAL SPACE
Resolution cutoff (Å)	3.4	3.5
$FSC_{model\text{-}vs\text{-}map}$=0.5 (Å)	3.5	3.6
**Model**	**PDB-9SKQ**	**PDB-9I9I**
Number of residues	1,205	908
Protein	1,201	902
Ligand	4	5
Water	0	1
B-factors overall	64.57	68.83
Protein	64.51	68.70
Ligand	74.55	85.10
Water	N/A	66.97
R.M.S. deviations		
Bond lengths (Å)	0.002	0.004
Bond angles (°)	0.597	0.960
**Validation**		
Molprobity score	1.77	1.67
Molprobity clashscore	9.04	9.59
Rotamer outliers (%)	0.28	1.63
C_β deviations (%)	0.00	0.00
Ramachandran plot		
Favored (%)	95.85	98.40
Allowed (%)	3.72	1.48
Outliers (%)	0.42	0.11

Table S4. DNA sequences of cloning primers and g-blocks used in this study.

Oligonucleotide designation	Sequence
Cyclin A2 cloning primer (forward)	TAC TTC CAA TCC AAT GCA ATG TTG GGC AAC TCT GCG CCG
Cyclin A2 cloning primer (reverse)	TTA TCC ACT TCC AAT GTT ATT ACA GAT TTA GTG TCT CTG GTG GG
CDK11 cloning primer (forward)	TAC TTC CAA TCC AAT GCA ATG TCT GAG GAT GAA GAA CGT G
CDK11 cloning primer (reverse)	TTA TCC ACT TCC AAT GTT ATT AAA ACT TAA GTG AGA AAC CGG G
CDK7 C-lobe linearisation primer (forward)	TTC GAA ACT CTG GGC ACC CCT AC
CDK7 C-lobe linearisation primer (reverse)	CAC CTG GTG GGT GTA AGC CCT
CDK7 N-lobe linearisation primer (forward)	GAA CTG AGC CAC CCT AAC ATC ATC
CDK7 N-lobe linearisation primer (reverse)	CTT GTA CAC GGT AGC GAA CTG TCC
$CDK7^{L219R}$ g-block	GG GCT TAC ACC CAC CAG GTG GTC ACT CGC TGG TAC AGG GCT CCT GAA CTG CTG TTC GGC GCC AGA ATG TAC GGC GTG GGA GTG GAC ATG TGG GCT GTG GGA TGC ATC CTG GCC GAG CTG CTG CTG AGG GTC CCA TTC CTG CCT GGT GAC TCC GAC AGG GAC CAG CTG ACC AGA ATC TTC GAA ACT CTG GGC ACC CC
$CDK7^{C3A}$ g-block	GG GCT TAC ACC CAC CAG GTG GTC ACT GCT TGG TAC AGG GCT CCT GAA CTG CTG TTC GGC GCC AGA ATG TAC GGC GTG GGA GTG GAC ATG TGG GCT GTG GGA TGC ATC CTG GCC GAG CTG CTG CTG AGG GTC CCA TTC CTG CCT GGT GAC GCT GAC CTG GAC CAG CTG GCT AGA ATC TTC GAA ACT CTG GGC ACC CC
$CDK7^{N2A}$ g-block	AG TTC GCT ACC GTG TAC AAG GCC CGC GAC AAG AAC ACT AAC CAG ATC GTC GCT ATC AAG AAG ATC AAG CTG GGT GCT GCT TCC GAG GCT AAG GAC GGC ATC AAC AGG ACC GCC CTG AGA GAG ATC AAG CTG CTG CAG GAA CTG AGC CAC CCT AAC AT
$CDK7^{N3A}$ g-block	AG TTC GCT ACC GTG TAC AAG GCC CGC GAC AAG AAC ACT AAC CAG ATC GTC GCT ATC AAG AAG ATC GCT CTG GGT GCT GCT TCC GAG GCT AAG GAC GGC ATC AAC AGG ACC GCC CTG AGA GAG ATC AAG CTG CTG CAG GAA CTG AGC CAC CCT AAC AT
$CDK2^{D145N}$ mutagenesis primer (forward)	CTA GCA AAC TTT GGA CTA GCC AGA
$CDK2^{D145N}$ mutagenesis primer (reverse)	TCC AAA GTT TGC TAG CTT GAT GG
CDK2 linearisation primer (forward)	GTG GTG TGG CCA GGA GTT ACT T
CDK2 linearisation primer (reverse)	TCC AGA TGT CCA CAG CTG TGG A
$CDK2^{I209R}$ g-block	CA GCT GTG GAC ATC TGG AGC CTG GGC TGC ATC TTT GCT GAG ATG GTG ACT CGC CGG GCC CTA TTC CCT GGA GAT TCT GAG CGT GAC CAG CTC TTC CGG ATC TTT CGG ACT CTG GGG ACC CCA GAT GAG GTG GTG TGG CCA GGA GTT

Data S1. Mass-spectrometry analysis of CDK2 phosphopeptides with and without incubation with CAK.
